# First Optimization of Novel, Potent, Selective PDE11A4 Inhibitors for Age-Related Cognitive Decline

**DOI:** 10.1021/acs.jmedchem.3c01088

**Published:** 2023-10-20

**Authors:** Shams ul Mahmood, Mariana Lozano Gonzalez, Sreedhar Tummalapalli, Jeremy Eberhard, Judy Ly, Charles S. Hoffman, Michy P. Kelly, John Gordon, Dennis Colussi, Wayne Childers, David P. Rotella

**Affiliations:** † Department of Chemistry & Biochemistry, Montclair State University, Montclair, New Jersey 07043, United States; ‡ Biology Department, Boston College, Chestnut Hill, Massachusetts 02467, United States; § Department of Anatomy & Neurobiology, School of Medicine, University of Maryland, Baltimore, Maryland 21201, United States; ∥ Moulder Center for Drug Discovery Research, Temple University, Philadelphia, Pennsylvania 19140, United States; ⊥ Sokol Institute of Pharmaceutical Life Sciences, Montclair State University, Montclair, New Jersey 07043, United States

## Abstract

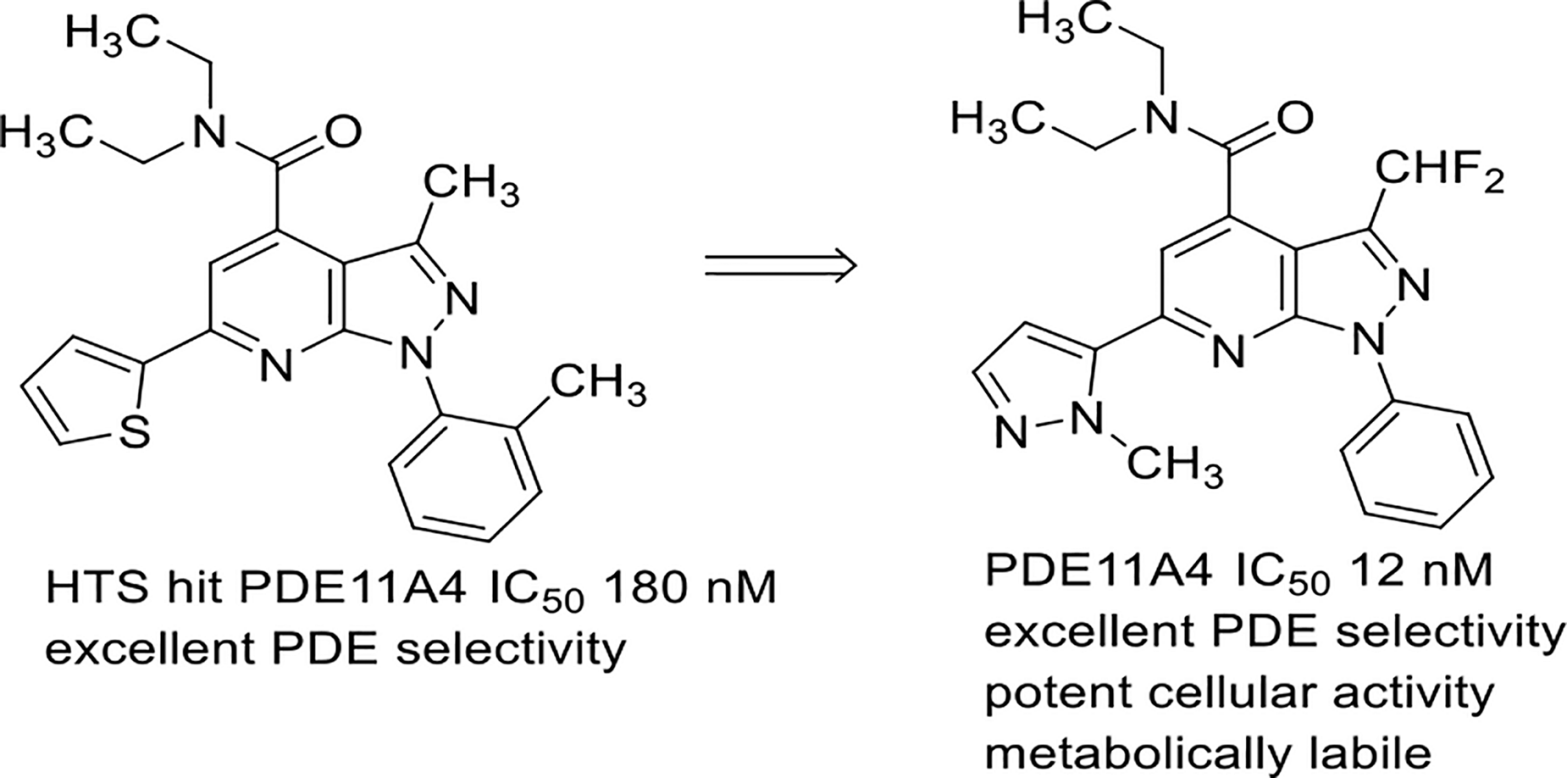

Phosphodiesterase 11A4 (PDE11A4) is a dual-acting cyclic nucleotide hydrolase expressed in neurons in the CA1, subiculum, amygdalostriatal transition area and amygdalohippocampal area of the extended hippocampal formation. PDE11A4 is the only PDE enzyme to emanate solely from hippocampal formation, a key brain region for the formation of long-term memory. PDE11A4 expression increases in the hippocampal formation of both humans and rodents as they age. Interestingly, PDE11A knockout mice do not show age-related deficits in associative memory and show no gross histopathology. This suggests that inhibition of PDE11A4 might serve as a therapeutic option for age-related cognitive decline. A novel, yeast-based high throughput screen previously identified moderately potent, selective PDE11A4 inhibitors, and this work describes initial efforts that improved potency more than 10-fold and improved some pharmaceutical properties of one of these scaffolds, leading to selective, cell-penetrant PDE11A4 inhibitors, one of which is 10-fold more potent compared to tadalafil in cell-based activity.

## Introduction

Phosphodiesterase 11A is a member of the superfamily of intracellular cyclic nucleotide hydrolases. The enzyme was originally cloned in 2000 and exists as a single gene with four isoforms.^1^ The longest isoform, PDE11A4, is ∼95% homologous across mouse, rat, and human.^2,3^ Tissue-specific distribution and function of other isoforms are discussed by others, and these isoforms are not present in the CNS.^2^ In the brain, PDE11A4 is strongly expressed in the neurons of the ventral hippocampal formation (VHIPP; a.k.a. anterior hippocampus in primates), with much lower levels of expression in the dorsal hippocampus as well as the adjacent amygdalohippocampal region and in some mice the nearby amygdalostriatal transition area.^4−6^ Outside of the brain, PDE11A4 expression was reliably measured in the spinal cord and dorsal root ganglion (i.e., present in wild-type but not *Pde11a* knockout mice), with no reliable PDE11A4 expression observed in 20 peripheral organs.^7,8^ This makes PDE11A4 unique because in brain, it is the only PDE to be expressed preferentially in the VHIPP, a structure critical for associative long-term memories.^5,9,10^ This makes PDE11A an attractive drug target because it stands to selectively restore aberrant cyclic nucleotide signaling in a brain region affected by various disease states without directly affecting signaling in other brain regions or peripheral organs. Indeed, *Pde11a* KO mice appear normal on a wide range of sensory, motor and anxiety/depression-related behaviors, show no gross peripheral histopathology at least up to 1 year of age (later ages not assessed), and reproduce normally.^5,18^

Interestingly, PDE11A4 expression in the hippocampus increases across the lifespan of both humans and rodents.^6^ This age-related increase in PDE11A4 is consistent with the literature, showing decreases in cyclic adenosine monophosphate (cAMP) and cyclic guanosine monophosphate (cGMP) in the aged and demented hippocampus (rodents and humans), particularly when there is a history of traumatic brain injury (TBI).^6,12−14^ In vitro and rodent studies show that age-related increases in PDE11A4 expression are driven by increased phosphorylation of the N-terminal regulatory domain at S117 and S124.^6^ Rodent studies also show these age-related increases in PDE11A4 expression drive age-related cognitive decline of social associative memories due to increased presence of the protein in the aged brain as opposed to a prolonged effect on the development of the brain.^6^ This is consistent with the fact that PDE11A4 regulates signals important for memory consolidation including glutamatergic and calcium/calmodulin-dependent kinase II (CamKII) signaling as well as protein synthesis.^4,5,9,15^ Together, these results suggest that a PDE11A4 inhibitor may hold promise for reversing some aspects of age-related cognitive decline.

**Figure 1:**
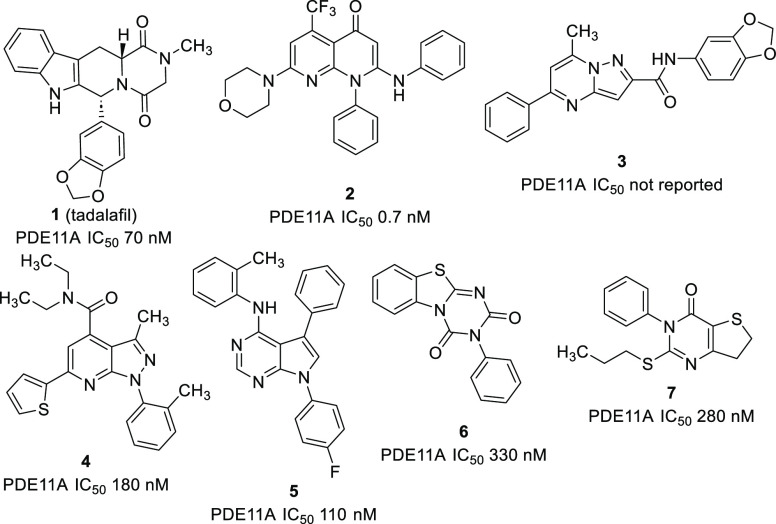
Examples of known PDE11A inhibitors.

Many PDEs have been the subject of drug discovery efforts;^11,15−17^ however, little attention has been devoted to PDE11. Tadalafil (**1**, Figure 1), an approved PDE5 inhibitor, is also known to inhibit PDE11A.^18^ Site-directed mutagenesis experiments using **1** suggest an important hydrogen bond between a glutamine residue in the active site and cyclic nucleotide substrates.^19^ There was a presentation of naphthyridine-based PDE11 inhibitors (**2**) as insulin secretagogues^20^ and a patent application claiming pyrazolopyrimidines (**3**) as PDE11 inhibitors.^21^ Ceyhan and colleagues described a yeast-based high throughput assay of approximately 200,000 compounds that identified a number of chemotypes.^22^ Briefly, this assay employed a yeast strain that expressed human PDE11A4 to permit the use of exogenous cGMP to activate PKA. Subsequently, a construct that expresses PDE11A4 at higher levels allowed for screens using human soluble adenylyl cyclase to produce intracellular cAMP. The yeast strains express orotidine monophosphate decarboxylase, whose activity is required for growth on medium lacking uracil and prevents growth on medium containing the pyrimidine analogue 5-fluoroorotic acid (5FOA).^23^ PDE11A4 activity in these yeast cells promotes colony formation on plates lacking uracil but prevents growth in the 5FOA medium. There is a correlation between the ED_50_ in this assay and IC_50_ values calculated in biochemical assays. This high throughput method is supplemented by an assay to evaluate yeast growth of selected compounds where activity is measured by a zone of inhibition on an agar plate.^23,24^ In this assay, aqueous solubility plays an essential role in efficacy. To be active in both assays, compounds must be cell permeable and have sufficient aqueous solubility, features essential for PDE11A inhibition and drug candidates in general. Four different chemotypes (**4**–**7**) that demonstrated exceptional PDE selectivity (at least 100×) and submicromolar PDE11A potency are shown in Figure 1. Based on the data in this report, we investigated the in vitro absorption, distribution, metabolism, elimination (ADME) properties of **4**–**7** to provide a more complete profile that would be used to prioritize them for optimization.

As shown in Table 1, each of these hits has positive and negative attributes. Tricycle **6** has good aqueous solubility and generally favorable metabolic stability in human and mouse microsomes, with a favorable CYP profile. However, it has fewer obvious vectors for optimization, and a screen of commercially available analogues furnished flat structure–activity data. Pyrrolopyrimidine **5** is highly lipophilic with poor aqueous solubility, a clean CYP profile, and good metabolic stability with three sites for optimization. Thienopyrimidinone **7** is metabolically unstable, and analysis of commercially available propylthio analogues was significantly less potent (>1 μM), suggesting this site would have limited value in optimization. Pyrazolopyridine **4** showed rapid oxidative metabolism with good aqueous solubility, is not a substrate for the p-glycoprotein pump with a favorable CYP profile, and has at least four sites that can be investigated. From a physical property viewpoint, the unfavorable cLogP (5.8, ChemDraw) and potential metabolic liability of the thiophene ring are clearly features to be addressed in optimization studies, and for these reasons, we chose **4** as a starting point for our work.

**Table 1:** Screening Hits In Vitro ADME Parameters

cpd	pH 7.4 PBS solub (μM)	mouse liver microsomal *t*_1/2_ (min)	Hu liver microsomal *t*_1/2_ (min)	IC_50_ (μM) Hu CYP3A4	IC_50_ (μM) Hu CYP2D6	IC_50_ (μM) Hu CYP2C9	MDCK efflux ratio
4	54	<2	<2	3	>10	>10	0.75
5	3	45	31	>10	>10	>10	NT
6	150	18	>60	1100	>10	>10	NT
7	25	<2	12	>10	>10	>10	NT

This paper describes the first report of optimization of a PDE11A4 inhibitor from a screening hit. Structure–activity is explored at four different positions to provide guidance on a significant improvement in potency, maintaining PDE selectivity, and improving selected pharmaceutical properties of **4**. It includes evaluation of selected compounds in a neuronal cell-based assay as a preliminary proof of concept for this enzyme as a drug target.

## Results

**Scheme 1:**
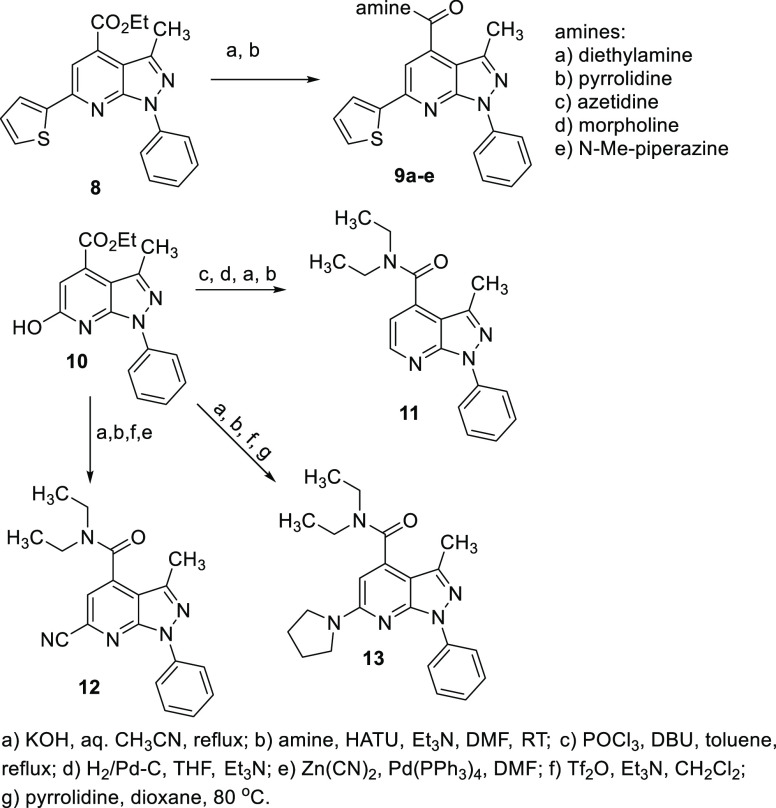
Synthesis of Amide Derivatives and Thiophene Alternatives

The C-6 heterocycle and amide substituents in **4** are the most straightforward sites to reduce lipophilicity and improve metabolic stability. To explore amide structure–activity relationships (SARs), we prepared thiophenyl-substituted pyrazolopyridine ester **8** using known chemistry (Scheme 1).^25^ Amides **9a**–**e** were then prepared by standard coupling methods. We also wanted to establish the contribution of the C-6 heterocycle and synthesized des-thiophenyl analogue **11** by chlorination of **10** with phosphorus oxychloride, followed by hydrogenolysis. To explore alternatives to an aromatic heterocycle, cyanopyridine **12** was obtained from **10** by selective ester hydrolysis, followed by amide and triflate formation and then palladium-(0)-mediated substitution using Zn(CN)_2_.^26^ Using the 2-chloropyridine intermediate derived from **10**, displacement with pyrrolidine provided **13**.

**Scheme 2:**
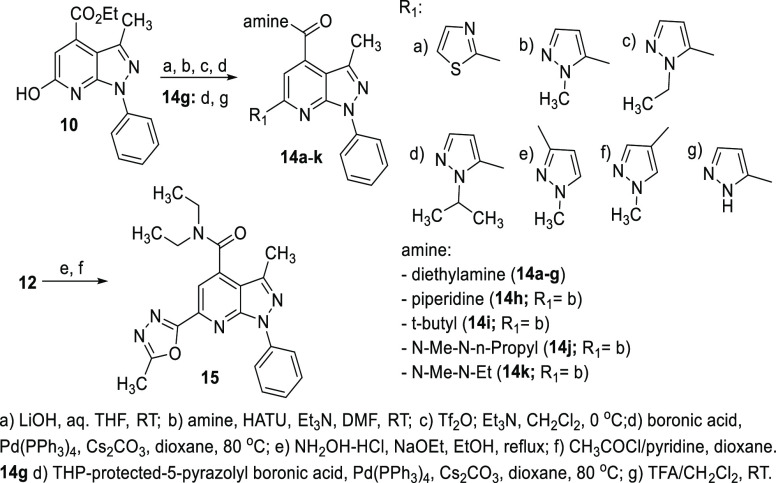
Synthesis of Heterocyclic PDE11A4 Inhibitors

Heterocyclic replacements to improve clogP and eliminate the potential metabolic liability of the thiophene ring were investigated using the diethylamide template. Suzuki coupling of the triflate derived from **10** was carried out (Scheme 2) using thiazole and regioisomeric pyrazole heterocycles, along with conversion of the nitrile in **12** to the corresponding oxadiazole **15**. Three *N*-alkyl variations of 5-substituted pyrazole **14b**–**d** and the unsubstituted pyrazole analogue **14g** were prepared to provide SAR at this position. Amide SAR was expanded beyond diethylamine, holding the C-6 heterocycle constant, to furnish **14h**–**k** (Scheme 2).

**Scheme 3:**
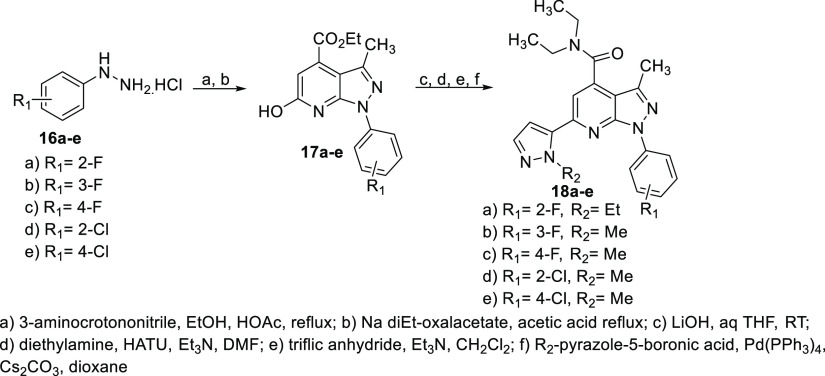
Synthesis of Halophenyl PDE11A4 Inhibitors

**Scheme 4:**
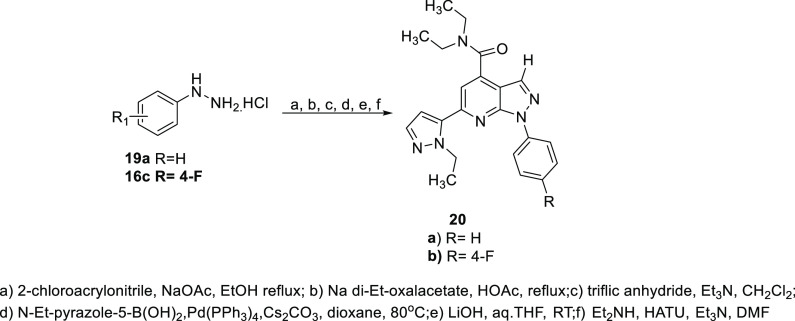
Synthesis of Desmethylpyrazolopyridine PDE11A4 Inhibitors

Monohalogenated phenyl-substituted analogues (**18a**–**e**) were prepared from the appropriate phenyl hydrazines **16a**–**e** using the reported route to **17a**–**e**^25^ and then as described above to targets **18a**–**e** (Scheme 3). Pyrazolopyridine examples **20a**–**b** lacking the C-3 methyl were synthesized in a similar manner beginning with 2-chloroacrylonitrile as shown in Scheme 4.^27^

**Scheme 5:**
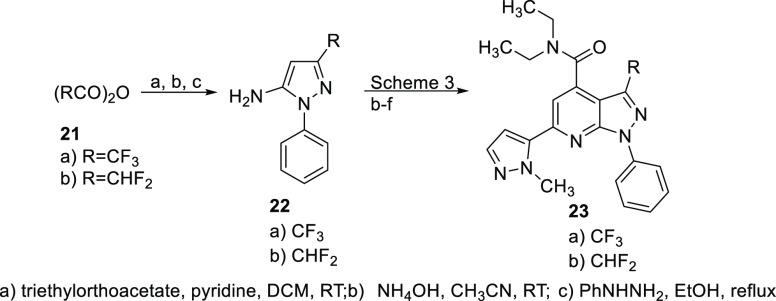
Synthesis of Di- and Trifluoromethyl PDE11A4 Inhibitors

**Scheme 6:**
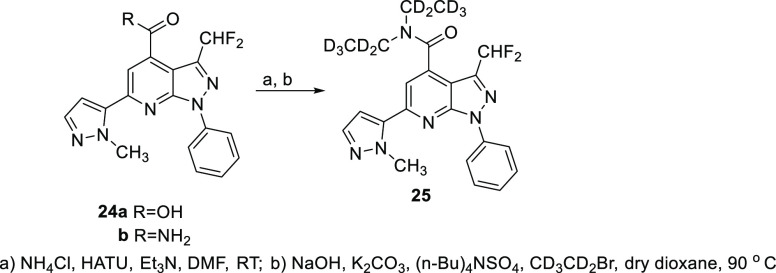
Synthesis of Perdeuterated Diethyl Amide

To provide additional SAR points on the pyrazolopyridine scaffold, we also prepared trifluoro- and difluoromethylpyrazolopyrimidines as outlined in Scheme 5 using chemistry identical to that shown in Scheme 3. The appropriately substituted aminopyrazole precursors **22a** and **22b** were synthesized as shown in Scheme 5. These were then converted to **23a** and **23b** as shown in Scheme 3. Based on metabolite identification data (vide infra) that the diethyl amide is a site for oxidative metabolism, we prepared a deuterated diethyl amide analogue of **23b** as shown in Scheme 6. Acid **24a**, obtained by hydrolysis of the appropriate ester precursor as described in Scheme 3, was converted to amide **24b** and then alkylated with *d*_5_-bromoethane under phase-transfer conditions to afford **25**.

The enzymes employed for biochemical assays are commercially available human PDEs. Initially all compounds were screened for inhibition of PDE11A4 at 50 and 500 nM to determine if IC_50_ determination was needed. In general, derivatives with approximately 80% inhibition at 500 nM progressed to IC_50_ determination. The examples studied to date all demonstrate equal inhibition of the PDE11A4-mediated hydrolysis of cAMP and cGMP. The IC_50_ values reported in Table 2 reflect cAMP-based data.

**Table 2:**
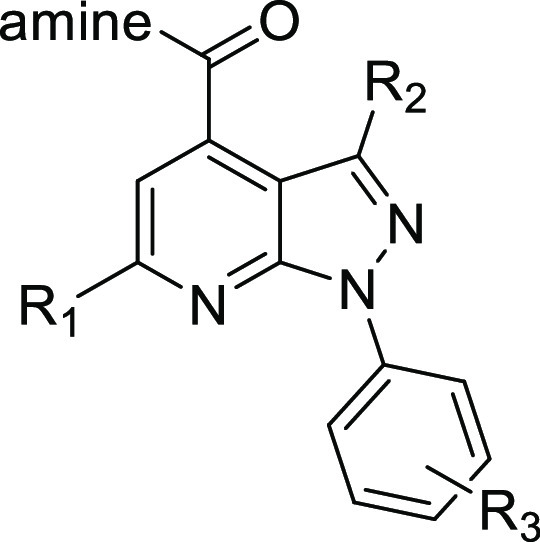
PDE11A4 Inhibition^a^

cpd	amine	R_1_	R_2_	R_3_	% inhib @ 500 nM	IC_50_ (nM)
9a	diethyl	2-thiophenyl	CH_3_	H		580
9b	pyrrolidinyl	2-thiophenyl	CH_3_	H	0	
9c	azetidinyl	2-thiophenyl	CH_3_	H	0	
9d	morpholinyl	2-thiophenyl	CH_3_	H	0	
9e	N-Me-piperazinyl	2-thiophenyl	CH_3_	H	0	
11	diethyl	H	CH_3_	H	0	
12	diethyl	CN	CH_3_	H	0	
13	diethyl	pyrrolidinyl	CH_3_	H	0	
14a	diethyl	2-thiazolyl	CH_3_	H		890
14b	diethyl	N-Me-5-pyrazolyl	CH_3_	H		53
14c	diethyl	N-Et-5-pyrazolyl	CH_3_	H		61
14d	diethyl	N-iPr-5-pyrazolyl	CH_3_	H	50	
14e	diethyl	N-Me-3-pyrazolyl	CH_3_	H	19	
14f	diethyl	N-Me-4-pyrazolyl	CH_3_	H	66	
14g	diethyl	NH-5-pyrazolyl	CH_3_	H	21	
14h	Piperidinyl	N-Me-5-pyrazolyl	CH_3_	H		4800
14i	t-Bu	N-Me-5-pyrazolyl	CH_3_	H	22	
14j	Me-n-propyl	N-Me-5-pyrazolyl	CH_3_	H		630
14k	Me-Et	N-Me-5-pyrazolyl	CH_3_	H		500
15	diethyl	2-Me-3,4-oxadiazolyl	CH_3_	H	0	
18a	diethyl	N-Et-5-pyrazolyl	CH_3_	2-F		62
18b	diethyl	N-Me-5-pyrazolyl	CH_3_	3-F	65	
18c	diethyl	N-Me-5-pyrazolyl	CH_3_	4-F		91
18d	diethyl	N-Me-5-pyrazolyl	CH_3_	2-Cl		51
18e	diethyl	N-Me-5-pyrazolyl	CH_3_	4-Cl	51	
20a	diethyl	N-Et-5-pyrazolyl	H	H		270
20b	diethyl	N-Et-5-pyrazolyl	H	4-F	31	
23a	diethyl	N-Et-5-pyrazolyl	CF_3_	H		81
23b	diethyl	N-Me-5-pyrazolyl	CHF_2_	H		12
25	CD_3_CD_2_	N-Me-5-pyrazolyl	CHF2	H		16
1						25

a IC_50_ values represent average of three independent determinations.

As shown in Table 2, the C-6 heterocycle plays an important role in PDE11A4 inhibition. Removal as in desthiophenyl **11** and substitution of cyano (**12**), pyrrolidine (**13**), and 2-methyloxadiazole **15** all furnish inactive analogues. Among the aromatic heterocycles evaluated, 5-substituted pyrazoles are superior to 3- and 4-substituted regioisomers (cf. **14b** vs **14e**, **14f**) and 2-thiazolyl analogue**14a**. Methyl- and ethyl-substituted 5-pyrazolyl **14b/c** are superior to the thiophenyl derivative **9a**. The NH analogue **14g** is less potent compared to **14b/c** as is the isopropyl derivative **14d.** This suggests a size-limited hydrophobic pocket for the nitrogen substituent that includes a regiochemical component based on the less potent activity of **14e** and **14f**.

Amide structure–activity is comparatively restricted, e.g., diethyl amide **9a** versus the inactive cyclic derivatives, **9b** (pyrrolidinyl) and **9c** (azetidinyl). One cyclic amide **14h** (piperidinyl) and a secondary amide (**14i**) with a bulky *t*-butyl group are weakly active (IC_50_ = 4800 nM and 22% at 500 nM, respectively). This binding pocket also appears to be lipophilic as the morpholino and *N*-methylpiperazinyl derivatives **9d** and **9e** are inactive. Interestingly, N-ethyl-*N*-methyl amide **14k** and *N*-methyl-N-propyl **14j** are ∼10-fold less potent compared to diethyl homologue **14b**.

Substitution on the phenyl ring is not necessary, evidenced by the moderate activity of **9a.** Halogenation at the ortho position (**18a** and **18d**) was explored to examine alternatives to the ortho-methyl group in **4**. These substitutions furnish derivatives with similar potency compared to **14b/c.** Other positions have variable effects on PDE11A4 potency; 4-fluoro (**18c**) is similar to its unsubstituted parent **14b**, while 3-fluoro (**18b**) is less potent compared to the 2- and 4- regioisomers or the unsubstituted parent. The 4-chloro analogue **18e** is less potent compared to either unsubstituted phenyl or 2- and 4-fluoro analogues.

A methyl group on the pyrazolopyridine scaffold is important for activity in this amide series, as evidenced by **20a** being 5-fold less potent compared to **14c**. It is notable in des-methyl analogue **20b**; 4-fluoro substitution on the phenyl ring leads to a significant decrease in activity compared to scaffold methylated analogue **18c**. Trifluoro- and difluoromethyl pyrazolopyrimidine derivatives **23a** and **23b** exhibited noteworthy differences in PDE11A4 potency. The trifluoromethyl analogue **23a** showed no real change in potency compared to methyl analogue **14c**. It was notable to observe that difluoromethyl **23b** was approximately 4- to 5-fold more potent compared to **14b/c**, with a PDE11A4 IC_50_ of 12 nM. As expected, deuterated **25** is equipotent to **23b**.

Selected examples of potent compounds (IC_50_ ∼ ≤ 100 nM) were initially evaluated for PDE selectivity versus human PDEs 3, 4, 5, 6, and 10. These were identified to establish activity against PDEs that are associated with known adverse events (PDE3,^11^ 4,^11^ 6,^11^ 10^28^) and because PDE5 is most closely related structurally to PDE11A4.^29^ The analogues (**14b, 14c, 18a, 18d**, and **23b**) are all diethyl amide derivatives with different single changes to provide matched pair evaluation. All compounds were tested in this panel at 50 and 500 nM because these concentrations were used in the initial PDE11A4 screening. Data are reported as percent inhibition at 500 nM in Table 3, and although not shown, in those instances where measurable inhibition was seen, there was a corresponding decrease at 50 nM.

**Table 3:** PDE Selectivity^a^

cpd	PDE3A	PDE4D3	PDE5A	PDE6C	PDE10A
14b	0	1.1	9.7	13.5	13.8
14c	0.8	6	36	20	18
18a	3	8	61	30	2
18d	0	0	48	12	5
23b	2	8	20	7	8

a % inhibition at 500 nM, average of three independent determinations.

Pyrazolyl-substituted examples **14b** and **14c** with different alkyl groups (methyl and ethyl, respectively) on the pyrazole show good selectivity against the PDEs in this panel, with **14c** showing a modest increase in PDE5 inhibition, and **14b** gives a profile similar to **4**. 2-Fluoro or 2-chloro substitution (**18a** and **d**, respectively) on the phenyl ring shows a small increase in PDE5 inhibition compared to **14b/c** and otherwise retain the selectivity displayed by unsubstituted analogues **14b/c**. Difluoromethyl **23b** retains the high level of PDE selectivity displayed by **14b.** To explore PDE selectivity more completely, the simplest and most potent compounds, **14b** and **23b,** were evaluated at 1 and 10 μM versus PDEs 1, 2, 7, 8, and 9. These data (percent inhibition at 1 and 10 μM) are shown in Table 4. It is evident both of these compounds are selective for PDE11A4 versus other phosphodiesterase enzymes. The PDE selectivity for these analogues extends to isoforms of PDEs 1, 3, 4, 7, and 10 (Supporting Information).

**Table 4:** Expanded PDE Selectivity Screening of **14b, 23b**^a^

cpd	PDE1A1	PDE2A1	PDE7B	PDE8A1	PDE9A2
**14b** (1 μM)	1	27	0	1	2
**14b** (10 μM)	27	45	3	7	1
**23b** (1 μM)	3	12	2	1	1
**23b** (10 μM)	31	26	51	11	2

a % inhibition at 1 and 10 micromolar, average of two independent determinations.

**Figure 2:**
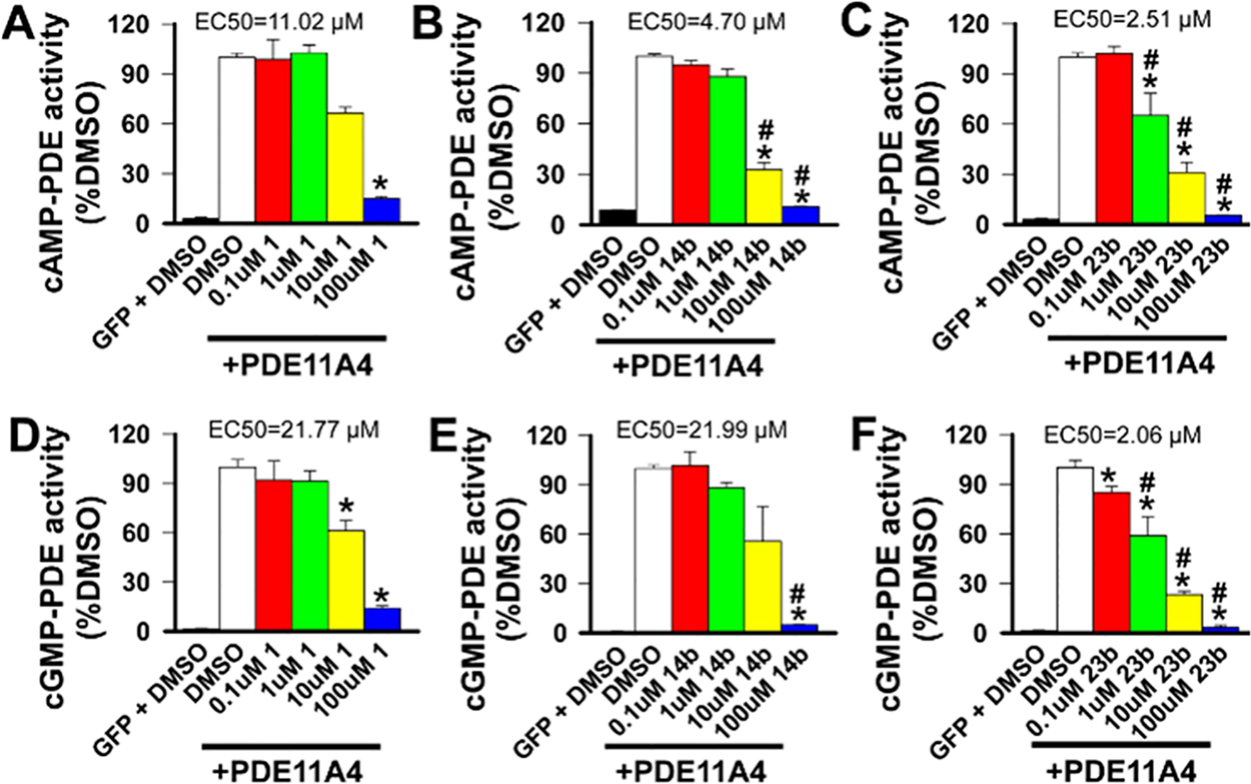
Efficacy of PDE11A inhibitors in a cell model of aging-like PDE11A4 protein abnormalities. (A) **1**, (B) **14b**, and (C) **23b** all reduce PDE11A4 cAMP hydrolytic activity, but **23b** appears more potent as it was the only compound with robust inhibition noted at 1 μM (note that **1** and **23b** were simultaneously cultured and processed). The same pattern was observed for the ability of (D) **1**, (E) **14b**, and (F) **23b** to inhibit PDE11A4 cGMP hydrolytic activity, again with **23b** exhibiting greater potency. Comparison of 100 μM dose groups across compounds suggests that **14b** and **23b** are both more efficacious than **1**, with both showing stronger inhibition of PDE11A4 cAMP and cGMP hydrolytic activity. *vs DMSO + PDE11A4 within experiment, *P* < 0.05–0.001; #vs **1** at the same concentration, *P* < 0.05–0.001. Data graphed mean ± SEM.

Next, we tested the therapeutic potential of selected PDE11A4 inhibitors in a cell-based model that mimics the age-related abnormalities in PDE11A4 expression and compartmentalization that are observed in the hippocampus.^30^ HT-22 hippocampal cells that do not express PDE11A4 were transfected to express either green fluorescent protein (GFP) as a negative control or human PDE11A4, with the difference between vehicle treated groups (i.e., DMSO) representing the PDE11A4-mediated catalytic activity (*n* = 4 different biological replicates per treatment group). Using tadalafil (**1**) as a positive control at 0.1, 1, 10, and 100 μM, a concentration-dependent and statistically significant (*P* ≤ 0.05) reduction versus control-treated cells in cAMP- and cGMP-PDE11A4 activity was observed (Table 5). Statistical analysis of data is provided in Figure 2 and in the Experimental Section). Diethylamide **14b** similarly reduced both cyclic nucleotides in a statistically significant, concentration-dependent manner (*P* ≤ 0.05). Notably, difluoromethyl analogue **23b** furnished a greater effect at the three highest concentrations tested compared to **1**, leading to a significant improvement in EC_50_ for both cyclic nucleotides (*P* ≤ 0.05). These cellular data provide initial in vitro support in an appropriate cell type for the expected effects of PDE11A4 inhibition. These effects are not due to an effect on protein expression or cell viability at any of the tested concentrations (see the Supporting Information for full details).

**Table 5:** PDE11A4 Inhibitors **1, 14b** and **23b** Cell-Based Efficacy^a^

cpd	EC_50_ (μM) cAMP	EC_50_ (μM) cGMP
1	11	22
14b	4.7	22
23b	2.5	2.1

a EC_50_ values represent the average of four independent replicates per treatment group. See the Experimental Section for statistical analysis.

As noted in Table 1, pyrazolopyrimidine **4** underwent rapid oxidative metabolism in human and mouse liver microsomes. Replacement of the thiophene ring with an N-alkyl-5-pyrazole did not improve metabolic stability (Table 6, **14b** and **14c**); however this modification did increase aqueous solubility (135 and 120 μM, respectively, versus 54 μM) and cLogP (3.74 and 4.08, respectively). This substitution also altered the CYP profile compared to **4**, reducing inhibition of 3A4 (cf. **4** vs **14b**), and increasing 2D6 inhibition. It is interesting to note the improved 2D6 selectivity of **14c** compared to **14b** accompanied by a slight increase in CYP2C9 inhibition. Replacement of the thiophene ring with *N*-methylpyrazole in **14b** maintained the favorable MDCK profile with an efflux ratio of 0.85.

**Table 6:** In Vitro ADME Parameters for Selected PDE11A4 Inhibitors

cpd	pH 7.4 PBS solub (μM)	mouse liver microsomal *t*_1/2_ (min	human liver microsomal *t*_1/2_ (min	IC_50_ (μM) Hu CYP3A4	IC_50_ (μM) Hu CYP2D6	IC_50_ (μM) Hu CYP2C9
14b	135	2.3	3.2	10	1.4	4.4
14c	120	1.9	4.2	>10	>10	1.5
23b	42	3.6	4.3	5.8	>10	5.6
25	39	5.2	8	7.7	>10	>10

Preliminary metabolite identification was carried out to investigate site(s) for metabolism in **14b**. Based on LCMS/MS data of metabolites derived from microsomal incubation, the diethyl amide was highlighted as a primary site for oxidative transformation. This analysis did not reveal any oxidative metabolism on either the pyrazole or phenyl substitutents. Accordingly, we chose to evaluate deuteroethyl analogue **25.** As shown in Table 6, **25** shows little improvement in microsomal stability. The primary site of metabolism in **25** remains the deuterated alkyl groups on the amide, evidenced by a loss of 18 mass units (CD_3_) in the primary metabolite. Subsequent metabolism of compound **25** occurred in this region of the compound.

Difluoromethyl amide **23b,** like **4** and **14b**, does not show efflux potential in MDCK-MDR1 cell culture, with an efflux ratio of 0.73. This change results in a decrease in aqueous solubility compared to that of **14b** to less than 50 μM. There is no change in 2C9 inhibition, an improved 2D6 profile, and a small increase in CYP3A4 inhibition. Deuteration of the alkyl groups improved the CYP profile of **25**, resulting in a significant decrease in the level of CYP2C9 inhibition and a small decrease in the level of CYP3A4 inhibition. As expected, other properties remained unchanged.

## Discussion and Conclusions

Prior to this research, there was a single report of optimization of PDE11A inhibitors in a poster presentation.^20^ This article details structure–activity exploration of four different points in a novel pyrazolopyridine scaffold to furnish PDE11A4 inhibitors with improved potency and aqueous solubility compared to an HTS hit. The lipophilic and metabolically labile thiophene ring was replaced by certain *N*-alkyl 5-pyrazoles (**14b/c**) to improve potency and reduce lipophilicity. Removal of the methyl group from the pyrazolopyridine scaffold reduced the PDE11 potency. Further examination of this position revealed that a difluoromethyl moiety provides a measurable improvement in enzymatic activity compared to methyl or trifluoromethyl. We are now actively investigating an expanded set of analogues that incorporate the difluoromethyl substituent on the pyrazolopyridine template to explore the generality of this observation. Halogen substitution on the pendant phenyl ring has variable effects depending on the position and halogen. Ortho substitution with both fluorine and chlorine is equivalent and no better than hydrogen. However, a 4-fluoro analogue is more potent than a 4-chloro derivative. Thus, previously unknown SAR was demonstrated at four sites in the hit **4** resulting in **23b**, a highly selective PDE11A4 inhibitor with more than a 10-fold improvement in enzymatic potency compared to **4**.

Selectivity in this series versus other PDEs was dependent on the nature and location of the substituents, and two examples (**14b** and **23b**) show high selectivity for PDE11A4. This selectivity includes isoforms of PDEs 1, 4, and 7. Cell-based activity was demonstrated with two representative examples, and one of these (**23b**) was 10-fold more potent compared to tadalafil (**1**), a known PDE11A inhibitor. The cell-based experiment confirms the enzymatic data that this group of compounds is an equally effective inhibitor of both cyclic nucleotide substrates. This data set provides in vitro proof of principle that PDE11A4 inhibitors are efficacious in a neuronal cell line. A deuterated analogue (**25**) of the metabolically labile diethyl amide proved to be an unsuccessful solution to the microsomal instability observed with **4** and **14b**. We are continuing to explore other possibilities to address this limitation.

Ongoing structure-activity in this series is addressing further improvement in PDE11A4 potency and metabolic stability with the aim of identifying potentially orally bioavailable candidates to test the therapeutic hypothesis that inhibition of PDE11A4 is a therapeutic target for age-related cognitive decline. Progress toward these goals will be reported in due course.

## Experimental Section

### Compound Characterization

All reagents and solvents were used as received from commercial suppliers. All reactions were carried out under a nitrogen atmosphere unless otherwise stated. Compounds were analyzed using a CEM mini LC system with a Restek-C18 5 μm column (150 mm × 4.6 mm, 80% acetonitrile/water isocratic gradient over 6 min with UV detection at 254 nm). Thin-layer chromatography was performed on silica gel G plates with UV detection. All of the reported yields are for isolated products, and compounds were purified by automated flash chromatography (Teledyne Isco Rf200 + ). Proton NMR spectra were obtained at 400 MHz in CDCl_3_ unless otherwise stated. All final compounds except **18b** and **18e** had HPLC purities of at least 95% based on ^1^H NMR and HPLC analyses. Compounds **18b** and **18e** were 94 and 91% pure, respectively.

### Synthetic Procedures

#### Amides **9a–e**

Pyrazolopyridine ester **8** was synthesized as described.^25^ Ester hydrolysis was carried out using two equiv of LiOH in 25% aqueous THF at room temperature overnight. Following evaporation of THF, the pH was adjusted to 2 with 1 N HCl by pH paper to deposit a solid that was collected by filtration, washed with cold water, and dried to provide an off-white solid that was used without further purification.

The carboxylic acid (1 equiv) was dissolved in DMF at room temperature, and 3 equiv of triethylamine was added followed by HATU (2 equiv) and an appropriate amine (2 equiv). The reaction mixture was stirred at room temperature overnight, poured into ice water, and extracted with three portions of ethyl acetate. The combined organic layer was washed with two portions of 1 N HCl and brine, dried, and concentrated by rotary evaporation. Purification by silica gel chromatography eluting with hexanes and ethyl acetate provided pure products.

##### 9a

Yield 61%, ^1^H NMR (400 MHz, CDCl_3_) δ 8.37–8.35 (d, *J* = 8 Hz, 2H), 7.72–7.71 (d, *J* = 3.6 Hz, 1H), 7.55–7.51 (t, *J* = 7.6 Hz, 2H), 7.47–7.46 (d, *J* = 5.2 Hz, 1H), 7.41 (s, 1H), 7.29–7.27 (t, *J* = 7.4 Hz, 1H), 7.14 (m, 1H), 3.73 (br, 2H), 3.27–3.22 (q, *J* = 7.2 Hz, 2H), 2.56 (s, 3H), 1.38–1.35 (t, *J* = 6.8 Hz, 3H), 1.11–1.07 (t, *J* = 6.8 Hz, 3H).

##### 9b

Yield 58%, ^1^H NMR (400 MHz, CDCl_3_) δ 8.36–8.34 (d, *J* = 7.6 Hz, 2H), 7.71–7.70 (d, *J* = 2.8 Hz, 1H), 7.54–7.50 (t, *J* = 7.6 Hz, 2H), 7.46 (s, 2H), 7.31–7.27 (t, *J* = 8 Hz, 1H), 7.14–7.12 (m, 1H), 3.78–3.75 (t, *J* = 6.8 Hz, 2H), 3.24–3.22 (t, *J* = 6.8 Hz, 2H), 2.54 (s, 3H), 2.07–2.00 (quintet, *J* = 6.8 Hz, 2H), 1.96–1.89 (quintet, *J* = 6.8 Hz, 2H).

##### 9c

Yield 69%, ^1^H NMR (400 MHz, CDCl_3_) δ 8.36–8.34 (d, *J* = 8.4 Hz, 2H), 7.72–7.71 (d, *J* = 3.6 Hz, 1H), 7.54–7.50 (t, *J* = 7.2 Hz, 2H), 7.46 (s, 2H), 7.31–7.25 (t, *J* = 7.6 Hz, 1H), 7.15–7.13 (t, *J* = 3.6 Hz, 1H), 4.34–4.30 (t, *J* = 7.6 Hz, 2H), 4.02–3.98 (t, *J* = 7.6 Hz, 2H), 2.65 (s, 3H), 2.44–2.36 (quintet, *J* = 7.6 Hz, 2H).

##### 9d

Yield 74%, ^1^H NMR (400 MHz, CDCl_3_) δ 8.35–8.33 (d, *J* = 8.4 Hz, 2H), 7.72–7.71 (d, *J* = 3.6 Hz, 1H), 7.55–7.51 (t, *J* = 7.6 Hz, 2H), 7.48–7.47 (d, *J* = 5.2 Hz, 1H), 7.41 (s, 1H), 7.32–7.28 (t, *J* = 7.6 Hz, 1H), 7.16–7.13 (t, *J* = 4 Hz, 1H), 3.93–3.92 (apparent d, *J* = 3.2 Hz, 2H), 3.86–3.85 (apparent d, *J* = 4 Hz, 2H), 3.61 (br s, 2H),3.33 (br s, 2H), 2.58 (s, 3H).

##### 9e

Yield 63%, ^1^H NMR (400 MHz, CDCl_3_) δ 8.35–8.33 (d, *J* = 8.4 Hz, 2H), 7.72–7.71 (d, *J* = 3.6 Hz, 1H), 7.55–7.51 (t, *J* = 7.2 Hz, 2H), 7.48–7.46 (d, *J* = 4.8 Hz, 1H), 7.41 (s, 1H), 7.31–7.28 (t, *J* = 7.2 Hz, 1H), 7.15–7.13 (t, *J* = 3.6 Hz, 1H), 4.03 (br, 2H), 3.44 (br, 2H), 2.72 (br, 2H), 2.56 (s, 3H), 2.43 (br, 5H).

#### 11

Intermediate **10** was synthesized as described.^26^ 200 mg of **10** (0.67 mmol) was dissolved in 3 mL of toluene, and DBU (0.74 mmol, 0.11 mL) was added, followed slowly by a solution of POCl_3_ (6.7 mmol, 0.062 mL) in 7 mL of toluene. The resulting orange solution was heated to reflux for 4 h, then cooled to room temperature, and quenched by careful addition of saturated NaHCO_3_ solution. The aqueous layer was extracted with three portions of ethyl acetate, and the combined organic extracts were washed with brine, dried, and used without further purification. The crude material (∼100 mg) was dissolved in 10 mL of THF to which 2 mL of triethylamine was added, followed by 30 mg of 10% Pd–C. The suspension was placed on a Parr shaker under 45 psi hydrogen overnight. After removal of the catalyst by filtration through Celite and careful washing with ethanol, the filtrate was concentrated by rotary evaporation and purified by silica gel chromatography eluting with hexanes/ethyl acetate to furnish 62 mg (0.22 mmol, 69% yield) as a glassy solid. This ester was hydrolyzed and then converted to the corresponding diethyl amide as described above to furnish **11** as an off-white solid (57% yield).

^1^H NMR (400 MHz, CDCl_3_) δ 8.62–8.61 (d, *J* = 4.6 Hz, 1H), 8.19–8.17 (d, *J* = 8.5 Hz, 2H), 7.54–7.02 (t, *J* = 7.6 Hz, 2H), 7.30–7.28 (t, *J* = 7.4 Hz, 1H), 7.04–7.03 (d, *J* = 4.5 Hz, 1H), 3.83 (br, 2H), 3.21–3.19 (q, *J* = 6.7 Hz, 2H), 2.58 (s, 3H), 1.37–1.33 (t, *J* = 7.2 Hz, 3H), 1.10–1.06 (t, *J* = 7.1 Hz, 3H).

#### 12

Ester **10** (1.0 g, 3.4 mmol) was dissolved in 40 mL of dichloromethane and cooled in an ice bath. Pyridine (54 mg, 6.8 mmol, 0.54 mL) was added, followed slowly by triflic anhydride (1.15 g, 4.0 mmol, 0.68 mL). The reaction was stirred at ice bath temperature for 3 h, then diluted with 20 mL of dichloromethane, and washed with two portions each of 1 N HCl and brine. The organic extract was dried and concentrated, and the crude material was used without further purification.

Crude triflate (100 mg, 0.23 mmol) was dissolved in 5 mL of anhydrous DMF under nitrogen. Pd(PPh_3_)_4_ (26 mg, 0.023 mmol) was added, followed by 54 mg of Zn(CN)_2_ (0.46 mmol). The reaction was stirred at 80 °C for 4 h, then cooled, and poured into 30 mL of distilled water. The mixture was extracted with three portions of ethyl acetate, and the collected organic extracts were washed with brine, dried, concentrated, and purified by flash chromatography eluting with hexanes/ethyl acetate to furnish 65 mg (0.21 mmol, 92% yield) of the cyano ester as a bright yellow solid.

^1^H NMR (400 MHz, CDCl_3_) δ 8.26–8.23 (m, 2H), 7.61–7.57 (m, 2H), 7.44–7.41 (m, 2H), 3.84 (br, 2H), 3.26–3.24 (q, *J* = 7.2 Hz, 2H), 2.65 (s, 3H), 1.43–1.39 (t, *J* = 7.2 Hz, 3H), 1.18–1.15 (t, *J* = 7.2 Hz, 3H).

The cyano ester intermediate was dissolved in ethanol to which 1.0 N aqueous NaOH solution (2 equiv) was added. The solution was stirred at room temperature overnight and then concentrated to provide an aqueous residue whose pH was adjusted to 2 using 1 N HCl (pH paper). The mixture was extracted with three portions of ethyl acetate, and the collected organic extracts were washed twice with brine, dried, concentrated, and dissolved in 3 mL of DMF at room temperature. Diethylamine and HATU (2 equiv of each) were added. The reaction was stirred overnight at room temperature, poured into 20 mL of water, and extracted with three portions of ethyl acetate. The collected organic extract was washed twice with 1 N HCl and brine, dried, concentrated, and purified by silica gel chromatography eluting with hexanes/ethyl acetate to furnish a 45% net yield.

^1^H NMR (400 MHz, CDCl_3_) δ 8.41–8.38 (m, 2H), 7.53–7.49 (m, 2H), 7.27–7.25 (t, *J* = 7.4 Hz, 1H), 6.25 (s, 1H), 3.86 (br, 1H), 3.63 (br s, 4H), 3.34 (br, 1H), 3.30–3.28 (q, *J* = 7 Hz, 2H), 2.50 (s, 3H), 2.12–2.09 (m, 4H), 1.39–1.36 (t, *J* = 7 Hz, 3H), 1.15–1.12 (t, *J* = 7 Hz, 3H).

#### 13

Ester **10** (300 mg, 1.01 mmol) was hydrolyzed and converted to diethyl amide as described in the synthesis of amides **9a**–**e**. The resulting pyridone diethyl amide was reacted with triflic anhydride as described for **12** to provide a crude triflate. 50 mg (0.11 mmol) of this triflate was dissolved in 3 mL of dioxane to which 23 mg (0.33 mmol) of pyrrolidine was added. The reaction was heated to 80 °C overnight, then diluted with 25 mL of ethyl acetate, washed with two portions of water and brine, dried, concentrated, and purified by flash chromatography eluting with hexanes/ethyl acetate to furnish 28 mg (0.076 mmol) 69% yield.

^1^H NMR (400 MHz, CDCl_3_) δ 8.41–8.38 (m, 2H), 7.53–7.49 (m, 2H), 7.27–7.25 (t, *J* = 7.4 Hz, 1H), 6.25 (s, 1H), 3.86 (br, 1H), 3.63 (br s, 4H), 3.34 (br, 1H), 3.30–3.28 (q, *J* = 7 Hz, 2H), 2.50 (s, 3H), 2.12–2.09 (m, 4H), 1.39–1.36 (t, *J* = 7 Hz, 3H), 1.15–1.12 (t, *J* = 7 Hz, 3H).

### General Procedure for Suzuki Coupling

The appropriate pyrazolopyridine triflate (either an ester or an amide) was dissolved in dioxane. The desired boronic acid (2 equiv) and Cs_2_CO_3_ (3 equiv) were added to the solution under nitrogen. After flushing with nitrogen for five min, 10 mol % of Pd (PPh_3_)_4_ was added, followed by N_2_ flush for 5 min. The reaction mixture was stirred at 80 °C for overnight. Upon completion of reaction, the reaction mixture was poured into ice cold water and extracted with three portions of ethyl acetate, washed with water and brine, dried, and concentrated to afford a crude product. The crude product was purified by flash chromatography eluting with hexanes/ethyl acetate.

#### 14a

71% yield, ^1^H NMR (400 MHz, CDCl_3_) δ 8.36–8.34 (d, *J* = 8 Hz, 2H), 8.02 (s, 1H), 7.96–7.95 (d, *J* = 2.8 Hz, 1H), 7.57–7.50 (m, 3H), 7.34–7.30 (t, *J* = 7.2 Hz, 1H), 3.69–3.68 (br, 2H), 3.28–3.26 (q, *J* = 6.4 Hz, 2H), 2.65 (s, 3H), 1.38–1.34 (t, *J* = 6.8 Hz, 3H), 1.13–1.09 (t, *J* = 7.2 Hz, 3H).

#### 14b

66% yield, ^1^H NMR (400 MHz, CDCl_3_) δ 8.20–8.18 (d, *J* = 8.4 Hz, 2H), 7.53–7.49 (m, 3H), 7.35 (s, 1H), 7.33–7.29 (t, *J* = 7.2 Hz, 1H), 6.71 (s, 1H), 4.33 (s, 3H), 3.69 (br, 2H), 3.28–3.22 (q, *J* = 7.2 Hz, 2H), 2.58 (s, 3H), 1.38–1.34 (t, *J* = 7.2 Hz, 3H), 1.13–1.09 (t, *J* = 6.8 Hz, 3H).

#### 14c

74% yield, ^1^H NMR (400 MHz, CDCl_3_) δ 8.22–8.20 (dd, *J* = 8.7 Hz, 1 Hz, 2H), 7.62–7.61 (d, *J* = 2.1 Hz, 1H), 7.58–7.54 (t, *J* = 7.6 Hz, 2H), 7.42 (s, 1H), 7.39–7.37 (t, *J* = 7.3 Hz, 1H), 6.77 (d, *J* = 2 Hz, 1H), 4.85–4.83 (q, *J* = 7 Hz, 2H), 3.84 (br, 2H), 3.32–3.30 (q, *J* = 7 Hz, 2H), 2.64 (s, 3H), 1.57–1.54 (t, *J* = 7 Hz, 3H), 1.44–1.40 (t, *J* = 7 Hz, 3H), 1.18–1.15 (t, *J* = 7 Hz, 3H).

#### 14d

61% yield,^1^H NMR (400 MHz, CDCl_3_) δ 8.29–8.27 (dd, *J* = 8.8 Hz, 0.8 Hz, 2H), 7.66 (d, *J* = 2.0 Hz, 1H), 7.58–7.54 (t, *J* = 7.6 Hz, 2H), 7.39–7.35 (m, 2H), 6.70–6.69 (d, *J* = 2 Hz, 1H), 5.78–5.74 (sept, *J* = 6.4 Hz, 1H), 3.85 (br, 2H), 3.33–3.31 (q, *J* = 7.2 Hz, 2H), 2.65 (s, 3H), 1.64–1.62 (d, *J* = 6.4 Hz, 6H), 1.44–1.41 (t, *J* = 7.2 Hz, 3H), 1.19–1.16 (t, *J* = 7.2 Hz, 3H).

#### 14e

78% yield, ^1^H NMR (400 MHz, CDCl_3_) δ 8.36–8.34 (d, *J* = 8.4 Hz, 2H), 7.82 (s, 1H), 7.54–7.50 (t, *J* = 7.6 Hz, 2H), 7.44 (s, 1H), 7.30–7.28 (d, *J* = 7.2 Hz, 1H), 7.06 (s, 1H), 4.09 (s, 3H), 3.67 (br, 2H), 3.27–3.25 (q, *J* = 7.8 Hz, 2H), 2.57 (s, 3H), 1.37–1.33 (t, *J* = 7.2 Hz, 3H), 1.11–1.07 (t, *J* = 7.2 Hz, 3H).

#### 14f

70% yield, ^1^H NMR (400 MHz, CDCl_3_) δ 8.36–8.34 (dd, *J* = 8.7 Hz, 1 Hz, 2H), 8.11–8.09 (d, *J* = 9.9 Hz, 2H), 7.60–7.56 (t, *J* = 8.4 Hz, 2H), 7.35 (m, 1H), 7.26 (s, 1H), 4.05 (s, 3H), 3.83 (br, 2H), 3.31–3.29 (q, *J* = 7 Hz, 2H), 2.60 (s, 3H), 1.43–1.40 (t, *J* = 7 Hz, 3H), 1.16–1.13 (t, *J* = 7 Hz, 3H).

#### 14h

59% yield, ^1^H NMR (400 MHz, CDCl_3_) δ 8.2–8.18 (m, 2H), 7.55–7.50 (m, 3H), 7.35–7.26 (m, 2H), 6.74–6.73 (d, *J* = 2 Hz, 1H), 4.34 (s, 3H), 3.86–3.85 (d, *J* = 3.8 Hz, 2H), 3.29–3.28 (d, *J* = 3.8 Hz, 7.2 Hz, 2H), 2.60 (s, 3H), 1.75 (s, 4H), 1.51 (d, *J* = 1.6, 2H).

#### 14i

71% yield, ^1^H NMR (400 MHz, CDCl_3_) δ 8.16–8.14 (d, *J* = 7.6 Hz, 2H), 7.54–7.48 (m, 3H), 7.42 (s, 1H) 7.33–7.22 (m, 1H), 6.75 (s, 1H), 5.90 (s, 1H), 4.28 (s, 3H), 2.69 (s, 3H), 1.56 (s, 9H).

#### 14j

67% yield, ^1^H NMR (400 MHz, CDCl_3_) δ 8.20–8.18 (dd, *J* = 7.2 Hz, 2 Hz, 2H), 7.53–7.49 (t, *J* = 8 Hz, 3H), 7.36–7.35 (d, *J* = 3.6 Hz, 2H), 7.33–7.29 (t, *J* = 7.6 Hz, 1H), 6.74–6.71 (d, *J* = 11 Hz, 1H), 4.34–4.33 (d, *J* = 4.4 Hz, 3H), 3.61 (br, 1H), 3.21–3.16 (q, *J* = 5.2 Hz, 3H), 2.91 (s, 2H), 2.57–2.55 (d, *J* = 7.2 Hz, 3H), 1.80–1.78 (q, *J* = 7.6 Hz, 1H), 1.59–1.57 (q, *J* = 7.2 Hz, 1H), 1.07–1.04 (t, *J* = 7.2 Hz, 2H), 0.80–0.76 (t, *J* = 7.6 Hz, 2H).

#### 14k

63% yield, ^1^H NMR (400 MHz, CDCl_3_) δ 8.20–8.18 (d, *J* = 8.4 Hz, 2H), 7.54–7.49 (t, *J* = 8 Hz, 3H), 7.37 (s, 1H), 7.33–7.21 (t, *J* = 7.2 Hz, 1H), 6.74–6.72 (d, *J* = 8.4 Hz, 1H), 4.34 (s, 3H), 3.72 (br s, 1H), 3.30–3.25 (q, *J* = 6.8 Hz, 1H), 3.21 (s, rotamer 2 CH_3_), 2.90 (s rotamer 1, CH_3_), 2.57–2.56 (d, *J* = 5.6 Hz, 3H), 1.36–1.33 (q, *J* = 7.6 Hz, rotamer 2, CH_3_), 1.15–1.11 (q, *J* = 7.2 Hz, rotamer 1 CH_3_).

#### 14g

Suzuki coupling with THP-protected pyrazole-5-boronic acid was carried out as indicated. The product (100 mg, 0.21 mmol) was dissolved in 5 mL of dichloromethane at room temperature. 0.5 mL of trifluoroacetic acid was added, and the reaction was stirred at room temperature for 4 h. The reaction was diluted with 20 mL of dichloromethane; then, it was washed with two portions of saturated sodium bicarbonate solution and brine, dried, concentrated, and purified by silica gel chromatography eluting with 5% methanol in dichloromethane to furnish 56 mg (0.14 mmol) 69% yield.

^1^H NMR (400 MHz, CDCl_3_) δ 8.32–8.08 (m, 2H), 7.91 (br, 1H), 7.68–7.66 (m, 2H), 7.58–7.54 (m, 2H), 7.37–7.33 (m, 1H), 6.97 (s, 1H), 3.84 (br, 2H), 3.31–3.27 (q, *J* = 7.2 Hz, 2H), 2.62 (s, 3H), 1.43–1.40 (t, *J* = 7.2 Hz, 3H), 1.16–1.12 (t, *J* = 7.2 Hz, 3H).

#### 15

Cyano amide **12** (200 mg, 0.60 mmol) was dissolved in ethanol (10 mL) at room temperature. Hydroxylamine hydrochloride (46 mg, 0.66 mmol) was added, followed by 46 mg (0.66 mmol) of sodium ethoxide. The reaction mixture was heated to reflux overnight. The reaction mixture was poured into ice water and extracted with three portions of ethyl acetate. The collected organic extracts were washed with two portions of brine, dried, concentrated, and purified by flash chromatography eluting with hexanes/ethyl acetate to furnish 180 mg of aldoxime that was used for the next step. This material was dissolved in dioxane, and pyridine (1.2 equiv) was added at room temperature, followed by acetyl chloride (1.2 equiv) dropwise. The reaction mixture was heated to reflux overnight. Ice water was added, and the mixture was extracted with three portions of ethyl acetate. The collected organic extracts were washed with two portions of brine, dried, concentrated, and purified by flash chromatography eluting with hexanes/ethyl acetate to provide 60 mg (0.15 mmol) 25% net for two steps.

^1^H NMR (400 MHz, CDCl_3_) δ 8.36–8.34 (m, 2H), 7.93 (s, 1H), 7.61–7.57 (m, 2H), 7.38–7.31 (m, 1H), 3.74 (br, 2H), 3.32–3.27 (q, *J* = 7. Hz, 2H), 2.77 (s, 3H), 2.66 (s, 3H), 1.43–1.40 (t, *J* = 7.1 Hz, 3H), 1.18–1.14 (t, *J* = 7.2 Hz, 3H).

### Halogen-Substituted Pyrazolopyridines **18a–e**

An appropriate fluoro- or chloro-substituted phenyl hydrazine was dissolved in acetic acid, followed by addition of sodium diethyloxaloacetate (1.2 equiv). The reaction mixture was heated to reflux overnight, and acetic acid was removed by rotary evaporation. The residue was dissolved in ethyl acetate, washed twice with water and brine, dried, concentrated, and purified by flash chromatography eluting with hexanes/ethyl acetate to provide the desired halophenyl pyridone ester. The ester was hydrolyzed, followed by amide formation, as described above. Triflate formation was carried out as described above, followed by Suzuki coupling providing the target compounds **18a**–**e**.

#### 18a

59% yield ^**1**^H NMR (400 MHz, CDCl_3_) δ 7.67–7.65 (m, 1H), 7.54 (d, *J* = 1.8, 1H), 7.46 (m, 1H), 7.40 (s, 1H), 7.35–7.31 (m, 2H), 6.72 (d, *J* = 1.8 Hz, 1H), 4.70–4.65 (q, *J* = 6.9 Hz, 2H), 3.72 (br, 2H), 3.34–3.28 (q, *J* = 7 Hz, 2H), 2.62 (s, 3H), 1.41–1.38 (t, *J* = 7.1 Hz, 3H), 1.37–1.33 (t, *J* = 7.1 Hz, 3H), 1.17–1.14 (t, *J* = 7 Hz, 3H).

#### 18b

69% yield ^1^H NMR (400 MHz, CDCl_3_) δ 8.14–8.11 (d, *J* = 8.4 Hz, 2H), 7.57 (s, 1H), 7.57–7.45 (q, *J* = 8 Hz, 1H), 7.42 (s, 1H), 7.04–7.00 (t, *J* = 8.4 Hz, 1H), 6.77 (s, 1H), 4.39 (s, 3H), 3.85 (br, 2H), 3.30–3.25 (d, *J* = 6.8 Hz, 2H), 2.60 (s, 3H), 1.41–1.38 (t, *J* = 7.2 Hz, 3H), 1.16–1.12 (t, *J* = 6.8 Hz, 3H).

#### 18c

64% yield ^1^H NMR (400 MHz, CDCl_3_) δ 8.17–8.14 (dd, *J* = 8.6 Hz, 4.7 Hz, 2H), 7.51 (s, 1H), 7.38 (s, 1H), 7.20–7.16 (t, *J* = 8.5 Hz, 2H), 6.73 (s, 1H), 4.31 (s, 3H, 3.84 (br, 2H), 3.26–3.24 (d, *J* = 6.9 Hz, 2H), 2.57 (s, 3H), 1.37–1.34 (t, *J* = 7 Hz, 3H), 1.12–1.09 (t, *J* = 7 Hz, 3H).

#### 18d

60% yield ^1^H NMR (400 MHz, CDCl_3_) δ 7.65–7.62 (m, 1H), 7.58–7.55 (m, 1H), 7.52 (d, *J* = 2 Hz, 1H), 7.49–7.46 (m, 2H), 7.39 (s, 1H), 6.74–6.73 (d, *J* = 2 Hz, 1H), 4.19 (s, 3H), 3.74 (br, 2H), 3.33–3.31 (q, *J* = 7.2 Hz, 2H), 2.63 (s, 3H), 1.42–1.39 (t, *J* = 7.2 Hz, 3H), 1.19–1.15 (t, *J* = 7 Hz, 3H).

#### 18e

71% yield ^1^H NMR (400 MHz, CDCl_3_) δ 8.26–8.24 (m, 2H), 7.62–7.61 (d, *J* = 2.2 Hz, 1H), 7.55–7.52 (m, 2H), 7.42 (s, 1H), 6.79 (d, *J* = 2 Hz, 1H), 4.39 (s, 3H), 3.84 (br, 2H), 3.32–3.30 (q, *J* = 7 Hz, 2H), 2.63 (s, 3H), 1.44–1.40 (t, *J* = 7 Hz, 3H), 1.18–1.15 (t, *J* = 7 Hz, 3H).

#### 20a, 20b

To a mixture of the appropriate phenyl hydrazine hydrochloride salt (1 equiv) and 2-chloroacrylonitrile (1 equiv) in ethanol was added sodium acetate (2 equiv) and refluxed overnight. The reaction mixture was poured into ice cold water and extracted with three portions of ethyl acetate, washed with water and brine, dried, and concentrated. The desired amino pyrazole was purified by column using hexane-ethyl acetate. This amino pyrazole was condensed with sodium diethyl oxaloacetate as described above, followed by amide formation and Suzuki reaction to furnish the target compounds.

##### 20a

^1^H NMR (400 MHz, CDCl_3_) δ 8.23–8.21 (m, 3H), 7.60 (d, *J* = 1.9 Hz, 1H), 7.58–7.54 (m, 2H), 7.53 (s, 1H), 7.41–7.39 (t, *J* = 7.3 Hz, 1H), 6.78 (d, *J* = 2.0 Hz, 1H), 4.86–4.80 (q, *J* = 7 Hz, 2H), 3.73–3.71 (q, *J* = 7 Hz, 2H), 3.34–3.33 (q, *J* = 7 Hz, 2H), 1.56–1.53 (t, *J* = 7.2 Hz, 3H), 1.42–1.38 (t, *J* = 7.1 Hz, 3H), 1.19–1.16 (t, *J* = 7.1 Hz, 3H).

##### 20b

^1^H NMR (400 MHz, CDCl_3_) δ 8.20 (s, 1H), 8.19–8.16 (m, 2H), 7.60 (d, *J* = 2.4 Hz, 1H), 7.53 (s, 1H), 7.27–7.23 (m, 2H), 6.78–6.77 (d, *J* = 2.0 Hz, 1H), 4.82–4.77 (q, *J* = 7.2 Hz, 2H), 3.73–3.71 (q, *J* = 6.8 Hz, 2H), 3.34–3.33 (q, *J* = 7.2 Hz, 2H), 1.55–1.51 (t, *J* = 7.2 Hz, 3H), 1.42–1.38 (t, *J* = 7.2 Hz, 3H), 1.19–1.16 (t, *J* = 6.8 Hz, 3H).

#### 22a/b

To a solution of triethyl orthoacetate (1 equiv) and pyridine (2.2 equiv) in dichloromethane, the corresponding fluoroacetic anhydride (2 equiv) was added dropwise at 0 °C. The reaction mixture was warmed to room temperature and stirred overnight, at which time it was poured into cold sodium bicarbonate solution. The organic phase was washed with the water and brine, then dried, and concentrated to furnish a yellow liquid that was used without further purification. NH_4_OH solution (9 mL) was added to a solution of this intermediate (∼42 mmol) in acetonitrile and stirred for 6 h at room temperature. The reaction mixture was concentrated and diluted with dichloromethane. This solution was washed with water and brine and then concentrated to get a yellow solid which was used for the next step without purification. This crude material was dissolved in ethanol, and phenyl hydrazine (1.2 equiv) was added. The reaction mixture was refluxed overnight. After completion of the reaction, the mixture was poured into ice cold water and extracted with three portions of ethyl acetate. The organic extract was washed with water and brine, dried, and concentrated to furnish a solid that was used without further purification.

#### 23a, b

Using amino pyrazoles **22a/b**, procedures identical to those reported earlier were used to provide target compounds. Yields reported are based on Suzuki coupling of intermediate amide-triflate.

##### 23a

53% yield, ^1^H NMR (400 MHz, CDCl_3_) δ 8.15–8.13 (m, 2H), 7.64–7.60 (m, 4H), 7.52–7.48 (m, 1H), 6.84–6.83 (d, *J* = 2.0 Hz, 1H), 4.82–4.80 (br, 2H), 3.97–3.47 (br, 2H), 3.32–3.26 (q, *J* = 7.1 Hz, 2H), 1.53–1.49 (t, *J* = 7.2 Hz, 3H), 1.41–1.37 (t, *J* = 7.2 Hz, 3H), 1.18–1.14 (t, *J* = 7.2 Hz, 3H).

##### 23b

52% yield, ^1^H NMR (400 MHz, CDCl_3_) δ 8.20–8.18 (m, 2H), 7.61–7.56 (m, 4H), 7.46–7.42 (m, 1H), 7.19–6.92 (t, *J* = 54 Hz), 6.83–6.82 (d, *J* = 2.0 Hz, 1H), 4.36 (s, 3H), 3.71 (br, 2H), 3.34–3.28 (q, *J* = 7.2 Hz, 2H), 1.40–1.37 (t, *J* = 7.2 Hz, 3H), 1.17–1.14 (t, *J* = 7.2 Hz, 3H).

#### 25, 24b

To a solution of **24a** (obtained as outlined above-see procedures for amides **9a**–**e**, 0.13 mmol) in DMF was added HATU (0.15 mmol) and NH_4_Cl (0.26 mmol). Triethylamine (0.65 mmol) was added dropwise and stirred at room temperature overnight under nitrogen. The reaction mixture was poured into ice water, and the yellow precipitate that formed was collected by filtration, washed with water, and dried to furnish the desired amide in 78% yield.

^1^H NMR (400 MHz, DMSO) δ 8.62 (s, 1H), 8.18–8.16 (m, 4H), 8.12 (s, 1H), 7.78–7.63 (m, 4H), 7.53–7.49 (m, 1H), 7.22–7.22 (d, *J* = 2 Hz, 1H), 4.29 (s, 3H).

##### 25

Dry dioxane (10 mL) was added to **24b** (0.12 mmol) under N_2_. To this mixture K_2_CO_3_ (0.17 mmol), tetrabutylammonium hydrogen sulfate (0.12 mmol), and ground NaOH (0.5 mmol) were added. The reaction mixture was heated to 40 °C, and *d*_5_-bromoethane (0.76 mmol) was added dropwise. The reaction mixture was stirred at 90 °C overnight. The reaction was quenched by adding ice and was extracted using 3 × 10 mL of ethyl acetate. The collected organic extract was washed with water and brine. After drying and concentration, the product was purified by silica gel chromatography eluting with a hexane/ethyl acetate system in 54% yield.

^1^H NMR (400 MHz, CDCl_3_) δ 8.21–8.19 (m, 2H), 7.63–7.57 (m, 4H), 7.48–7.44 (m, 1H), 7.19–6.92 (t, *J* = 54 Hz, 1H), 6.83–6.82 (d, *J* = 2.0 Hz, 1H), 4.38 (s, 3H). M + H 435.

### PDE11A4 Enzymatic Assays

In vitro enzyme assays were conducted via the Ba(OH)_2_ precipitation method of Wang et al. using recombinant human PDE3A, PDE4D3, PDE5A, PDE6C, PDE10A1, and PDE11A4 (BPS Bioscience)).^29^ Substrate concentrations used were 15 nM cGMP (PDE3A), 18 nM cAMP (PDE3A) 200 nM cAMP (PDE4D3), 500 nM cGMP (PDE5A), 1.7 μM cGMP (PDE6C), 30 nM cAMP (PDE10A), 1.3 μM cGMP (PDE10A), 100 nM cGMP (PDE11A4), and 240 nM cAMP (PDE11A4). Inhibitor concentrations using a 10 point curve that reduces enzyme activity by 50% (IC_50_) are presented as calculated using an online IC_50_ Calculator (AAT Bioquest). Inhibitor concentrations of 4, 13.7, 40, 123, 370, 1110, 3330, and 10,000 nM were used. The values reported are means of at least three independent experiments. Substrate concentrations were ∼0.1 × *K*_M_ for each enzyme; thus, IC_50_ values approximate the *K*_i_ values.

### Cell-Based Assay

HT-22 cells (sex undefined) were cultured and transfected as previously described.^30^ Cells were maintained in T-75 flasks in Dulbecco’s modified Eagle’s medium (DMEM) with sodium pyruvate (GIBCO, Gaithersburg, MD or Corning, Manassas, VA), 1% penicillin/streptomycin (P/S, GE Healthcare Life Sciences, Logan, UT), and 10% fetal bovine serum (FBS; Atlanta Biologicals), with incubators set to 37 °C/5% CO_2_. Cells were passaged at ∼70% confluency using TrypLE Express (GIBCO; Gaithersburg, MD). The day before transfection, cells were plated in 60 mm dishes with DMEM+FBS+P/S. The day of transfection, the media was replaced with Opti-MEM (GIBCO), and cells were transfected using 5 μL of lipofectamine 2000 (Invitrogen; Carlsbad, CA), 1.875 ug of plasmid DNA, and 5 mL of Opti-MEM as per the manufacturer’s protocol. ∼ 19 h post transfection, the Opti-MEM/lipofectamine solution was replaced with DMEM+FBS+P/S. Cells continued growing for 5 h in the supplemented media and then were pharmacologically treated (0.01, 0.1, 1.0., 10, and 100 mM) for 1 h. After 1 h, the media was removed, the cells were harvested in buffer (20 mM Tris-HCl and 10 mM MgCl_2_) and homogenized using a tissue sonicator (output control: 7.5, duty cycle: 70, continuous), and then the samples were held at 4 °C until processing. Both cAMP- and cGMP-PDE activity were measured as previously described.^31^ Samples were incubated with 35000–45000 counts per minute (CPMs) of [^3^H]-cAMP or [^3^H]-cGMP for 10 min. The reaction was then quenched with 0.1 M HCl and neutralized by using 0.1 M Tris. Snake venom was then added to the sample and incubated for 10 min at 37 °C. Samples were then run down DEAE A-25 Sephadex columns previously equilibrated in high salt buffer (20 mM Tris-HCl, 0.1% sodium azide, and 0.5 M NaCl) and low salt buffer (20 mM Tris-HCl and 0.1% sodium azide). After washing the columns four times with 0.5 mL of low salt buffer, the eluate was mixed with 4 mL of scintillation cocktail, and then CPMs were read on a Beckman-Coulter liquid scintillation counter. Two reactions not containing any sample lysate were also taken through the assay to assess the background, which was subtracted from the sample CPMs. CPMs were then normalized as a function of total protein levels, which were quantified using the DC protein assay kit (Bio-Rad, Hercules, CA) according to the manufacturer’s directions.

#### Cellular PDE11A4 Activity Data Analysis

All between-group analyses were performed using Sigmaplot v11.2. EC_50_ calculations were performed using the Quest Graph online calculator (https://www.aatbio.com/tools/ic50-calculator; accessed 08/07/23). This calculator models an experimental set using a four-parameter logistic regression using the following formula:
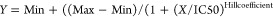


Each group contained four biological replicates, with one replicate per group processed together in a set. All data could not be analyzed together as (1) the experiments were not designed a priori to power analyses of complete dose responses between compounds and (2) a two-way repeated measure ANOVA failed normality (Shapiro-Wilk test) and equal variance (Levene’s test). Thus, treatment effects of an inhibitor (e.g., 0–100 μM of the same compound) were analyzed by repeated measure ANOVA (*F*) or repeated measure ANOVA on ranks (*X*^2^) when normality and/or equal variance failed (with sample sets paired for replicates processed in parallel). Group effects between experiments (e.g., 1 μM compound 1, 2 vs (3) were conducted by one-way ANOVA (*F*) or ANOVA on ranks (*H*) when normality and/or equal variance failed, and subsequent ANOVA *P*-values were adjusted for multiple comparisons using FDR-correction. In all cases, post hoc tests were conducted using the Student–Newman–Keuls method, and significance was defined as *P* < 0.05. Please note that Sigmaplot provides exact *P*-values for post hoc tests following a significant parametric ANOVA but only yes or no to “*P* < 0.05” for post hoc tests following a significant nonparametric ANOVA. Data are graphed mean ± standard error of the mean (SEM).

Statistical analysis of data from the HT22 cell-based assay to measure PDE11A4 activity (cAMP and cGMP levels):

**1:** cAMP (Figure 2A; *X*^2^ (5, *N* = 24)=16.86, *P* = 0.0048; post hoc: PDE11A4 + DMSO versus GFP + DMSO and PDE11A4 + 100 μM, *P* < 0.05 each) and cGMP-PDE11A4 (Figure 2D; *F*(5,15) = 50.03, *P* < 0.0001; post hoc: PDE11A4 + DMSO versus GFP + o μM, PDE11A4 + 10 μM, and PDE11A4 + 100 μM, *P* ≤ 0.002 each).
**14b:** cAMP-PDE11A4 enzymatic activity (Figure 2B; *X*^2^(5, *N* = 24)=19.0, *P* = 0.0019; post hoc: PDE11A4 + DMSO versus GFP + o μM, PDE11A4 + 10 μM,f and PDE11A4 + 100 μM, *P* < 0.05 each) and cGMP-PDE11A4 activity (*X*^2^(5, *N* = 24)=16.14, *P* = 0.0064; post hoc: PDE11A4 + DMSO versus GFP + DMSO and PDE11A4 + 100 μM, *P* < 0.05 each).
**23b**: cAMP-PDE11A4 activity (Figure 2C; *F*(5, 15)=62.36, *P* < 0.0001; post hoc: PDE11A4 + DMSO versus GFP + o μM, PDE11A4 + 1 μM, PDE11A4 + 10 μM, and PDE11A4 + 100 μM, *P* ≤ 0.0006 each) and cGMP-PDE11A4 activity noted at a log-fold lower concentration (Figure 2F; *X*^2^(5, *N* = 24) = 19.57, *P* = 0.0015; post hoc: PDE11A4 + DMSO versus GFP + o μM, PDE11A4 + 1 μM, PDE11A4 + 10 μM, and PDE11A4 + 100 μM, *P* < 0.05 each). One μM **23b** inhibited PDE11A4 to a greater extent than did 1 μM **1** (cAMP: *F*(2,9) = 4.95, FDR-P = 0.0355; cGMP: *F*(2,9) = 5.39, FDR-P = 0.029). A greater inhibition of PDE11A4 with 10 μM **23b** versus 10 μM **1** was also observed (cAMP: *F*(2,9) = 17.35, FDR-P = 0.0024; cGMP: H(2) = 7.27, FDR-P = 0.0239). At 100 μM, both **23b** and **14b** are more efficacious at inhibiting cAMP-PDE11A4 activity than 100 μM **1** in this in vitro model (Figure 2; H(2) = 9.85, FDR-P = 0.0003; post hoc: PDE11A4 + DMSO versus GFP + o μM, PDE11A4 + 10 μM,f and PDE11A4 + 100 μM, *P* < 0.05 each). The same is true for inhibition of cGMP-PDE11A4 (F(2,9) = 428.09, FDR-P = 0.0003; post hoc: **1** vs **14b** and **23b**, *P* = 0.004 each).

### In Vitro ADME Assays

#### Metabolic Stability

Microsomal stability was measured by incubating compounds at 37 °C in the presence of human or mouse liver microsomes and NADPH according to standard procedures.^32^ Aliquots were removed at five time points, quenched, and analyzed for remaining test compounds. Microsomal protein content was adjusted to give accurate rates of substrate consumption. Data were reported as compound clearance and compound half-life (*t*_1/2_). Analysis was performed by LC/MS/MS, and MSMS analyses used positive or negative electrospray or APCI ionization. Assay acceptance criteria are 20% for all standards and 25% for the LLOQ.

#### Aqueous Solubility

Thermodynamic aqueous solubility was measured by adding 2 mg of a solid test compound to 200 μL of deionized water in a filter plate. The plate was incubated for 72 h at room temperature, followed by vacuum filtration and analysis of the filtrate by UV or LCMS/MS following established protocols. Data were reported as the maximum concentration seen (mg/mL).

#### CYP Inhibition

Test compounds were assessed for their ability to inhibit the three major human cytochrome P450 enzymes, 3A4, 2D6, and 2C9. Expressed enzymes (obtained from insect supersomes) were used to minimize nonspecific binding and membrane partitioning issues.^33^ Briefly, recombinant CYP450 was incubated with an appropriate substrate in the presence and absence of NADPH at 37 °C. The 3A4 assay used midazolam as a substrate, and analysis was performed by LCMS/MS on a Waters Xevo TQ MS instrument (electrospray positive mode) coupled to a Waters Aquity UPLC. Propafenone was used as the internal standard. The 2D6 and 2C9 assays used fluorescent substrates [3-{2-(*N*,*N*,-diethyl-*N*-methylammonium) ethyl}-7-methoxy-4-methyl coumarin and 7-methoxy-4-(trifluoromethyl)-coumarin, respectively] and were analyzed on an Envision plate reader. IC_50_ values were determined using GraphPad’s Prism nonlinear curve fitting program.

#### MDCK-MDR1

MDCK cell monolayers (Absorption Systems, Malvern, PA) were grown to confluence on collagen-coated microporous membranes in 12-well assay plates. The assay buffer consisted of Hanks’ balanced salt solution containing 10 mM HEPES and 15 mM glucose at pH 7.4. The buffer in the receiver chamber contained 1% bovine serum albumin. Compounds were tested at a final concentration of 5 μM in the assay buffer. Cell monolayers were dosed on the apical side (A-B) or the basolateral side (B-A) and incubated at 37 °C with 5% CO_2_ in a humidified incubator. Samples were taken from the donor and receiver chambers at 120 min. Each determination was performed in duplicate. The flux of Lucifer yellow was also measured postexperimentally for each monolayer to ensure no damage was inflicted to the monolayer during the flux period. Samples were assayed on a Waters TQ LC/MS/MS instrument using positive or negative electrospray ionization.

## Supporting Information Available

Proton NMR and HPLC analyses, mass spectral data of **25**, expanded PDE selectivity of **14b** and **23b**, and protein expression and cell viability in the cell-based assay (PDF)
SMILES strings for all final compounds (CSV)

## ABBREVIATIONS

ADME: absorption, distribution, metabolism, elimination
cAMP: cyclic adenosine monophosphate
cGMP: cyclic guanosine monophosphate
CYP: cytochrome P450
MDCK1: Madin-Darby Canine kidney cells
PDE11A4: phosphodiesterase type 11A isoform 4
SAR: structure–activity relationships
