# Supplementary material for: First Optimization of Novel, Potent, Selective PDE11A4 Inhibitors for Age-Related Cognitive Decline
Source: J Med Chem. 2023 Oct 20;66(21):14597–608. doi: 10.1021/acs.jmedchem.3c01088 (PMC10641827; doi:10.1021/acs.jmedchem.3c01088)
Supplement: Supplementary file 1 — jm3c01088_si_001.pdf [file jm3c01088_si_001.pdf]

Supplementary Information

The First Optimization of Novel, Potent, Selective PDE11A4 Inhibitors for Age-Related Cognitive Decline

Shams ul Mahmood[1,5], Mariana Lozano Gonzalez[1,5+], Sreedhar Tummalapalli[1,5+], Jeremy Eberhard[2], Judy Ly[2], Charles S. Hoffman[2], Michy P. Kelly[3], John Gordon[4], Dennis Colussi[4], Wayne Childers[4], David P. Rotella*[1,5]

[1]Department of Chemistry & Biochemistry, Montclair State University, Montclair, NJ 07043, [2]Biology Department, Boston College, Chestnut Hill MA, 02467, [3]Department of Anatomy & Neurobiology, School of Medicine, University of Maryland, Baltimore MD 21201, [4]Moulder Center for Drug Discovery Research, Temple University, Philadelphia PA 19140. [5]Sokol Institute of Pharmaceutical Life Sciences, Montclair State University, Montclair NJ 07043

Index

1) Proton NMR pages S2-S17
2) HPLC pages S17-S32
3) Mass spectrum of compound **25** page S33
4) Expanded PDE selectivity of **14b** and **23b** pages S34
5) Protein expression and cell viability in cell-based assay pages S35-S36

Proton NMR and HPLC data of Final Compounds

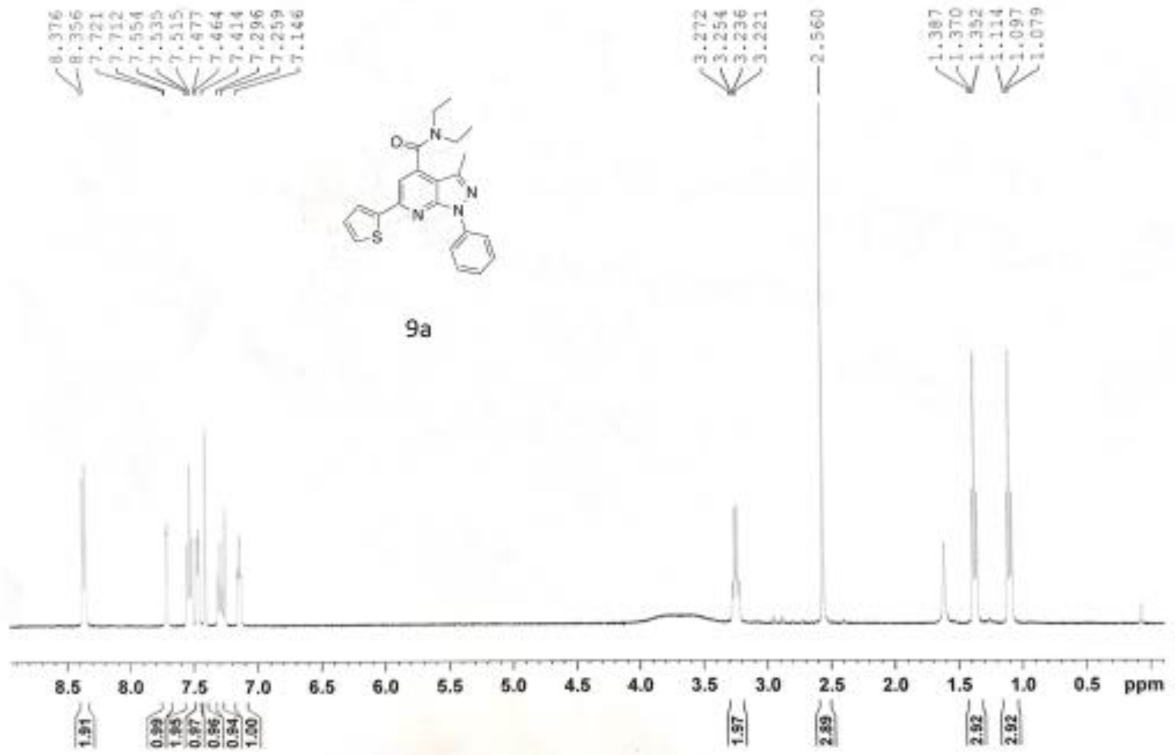

9a

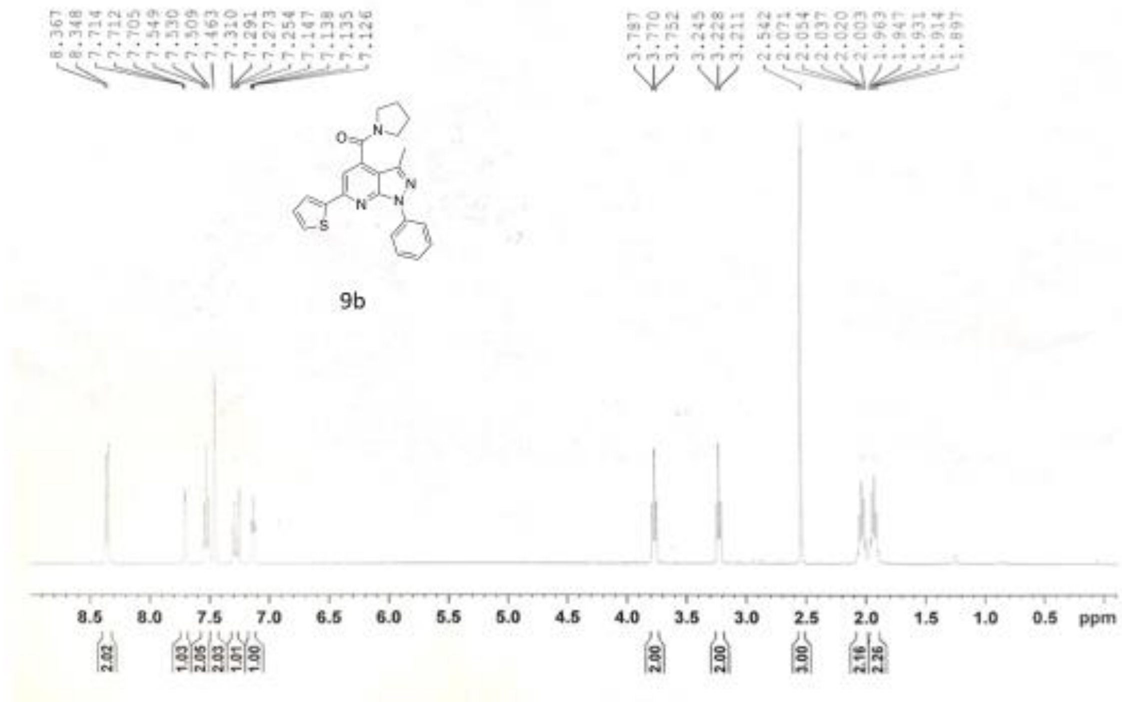

9b

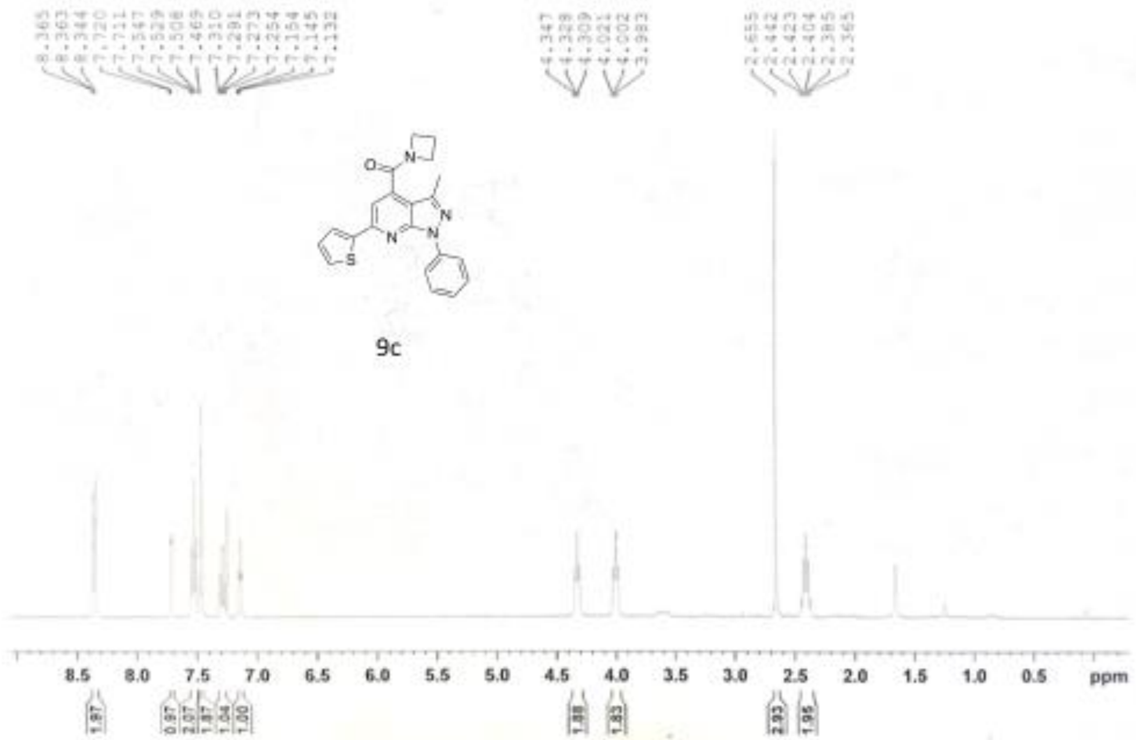
9c
ppm

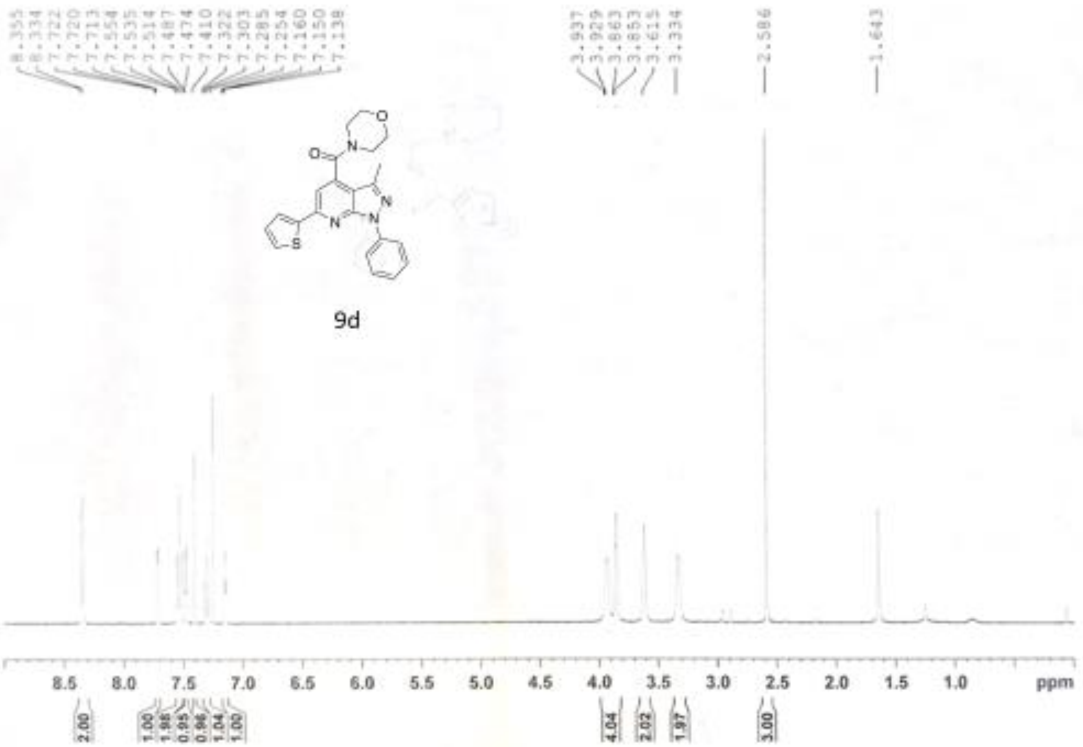
9d
ppm

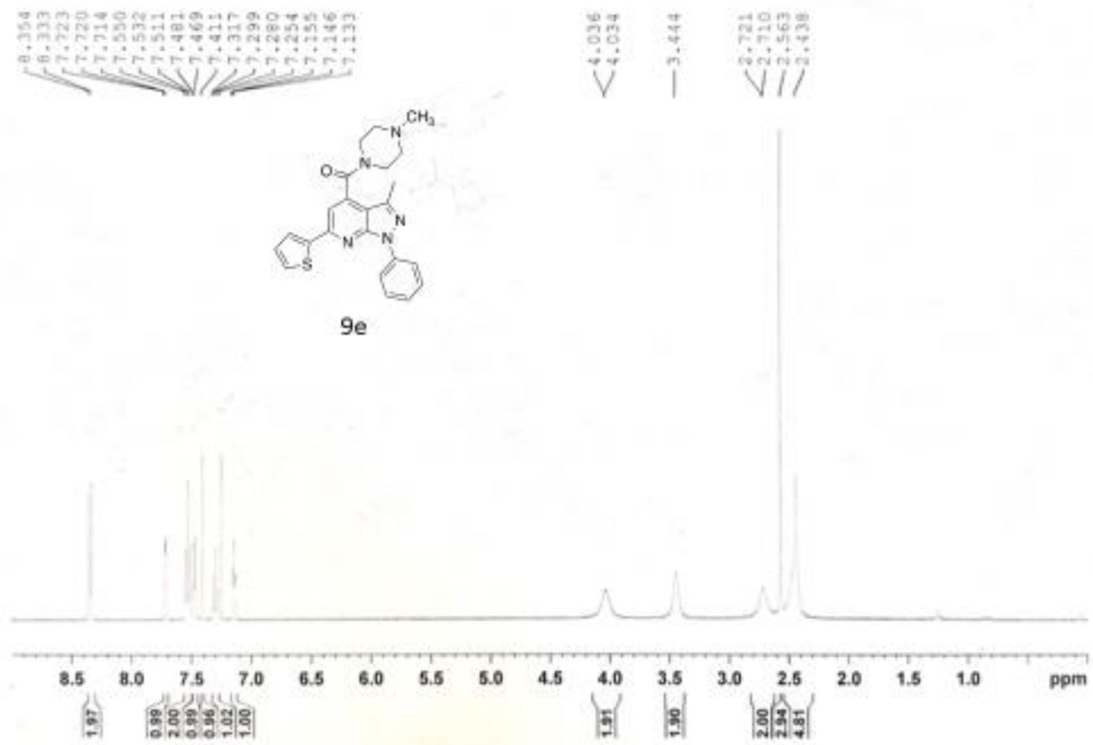
8.354 8.333 7.723 7.714 7.550 7.532 7.511 7.481 7.469 7.411 7.317 7.299 7.280 7.254 7.155 7.146 7.133
4.036 4.034
3.444
2.721 2.710 2.563 2.438
CH3
O
N
N
N
N
S
8.5 8.0 7.5 7.0 6.5 6.0 5.5 5.0 4.5 4.0 3.5 3.0 2.5 2.0 1.5 1.0 ppm
1.97 0.99 2.00 0.99 0.96 1.02 1.00 1.91 1.90 2.00 2.94 4.81

9e

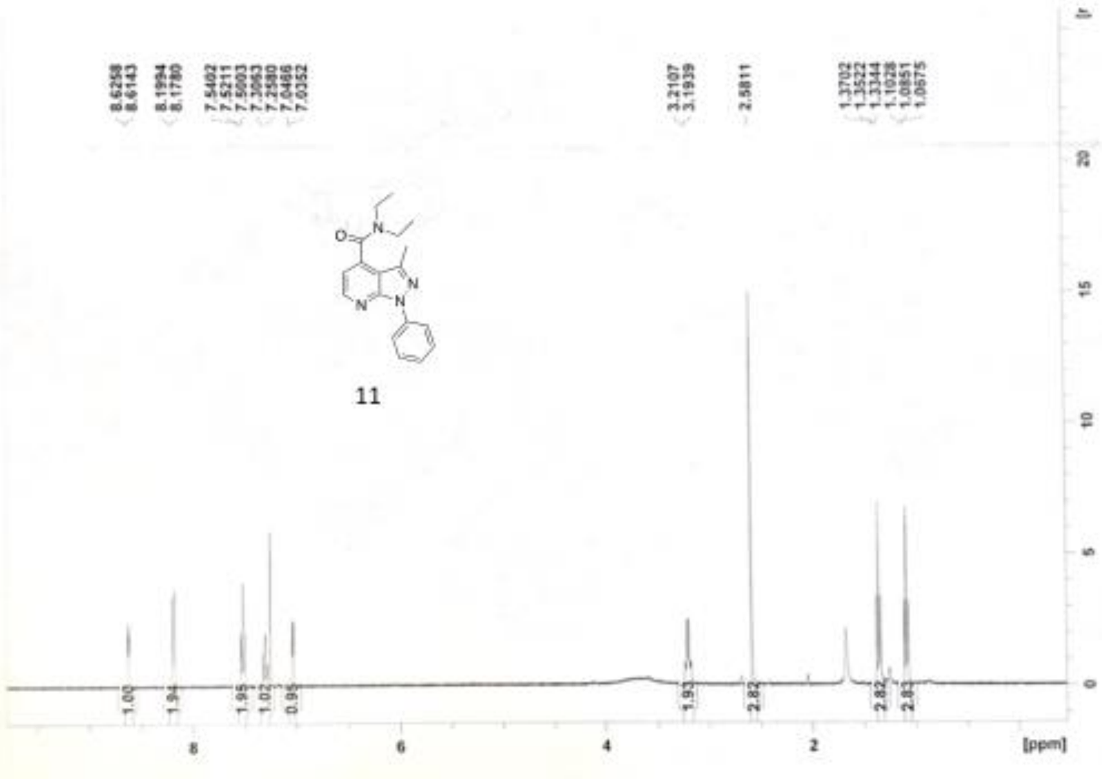
8.6258 8.6143 8.1994 8.1780 7.5402 7.5211 7.5003 7.3663 7.2580 7.0466 7.0352
3.2107 3.1939
2.5811
1.3702 1.3522 1.3344 1.1028 1.0851 1.0675
O
N
N
N
N
1.00 1.94 1.95 1.02 0.95 1.93 2.82 2.82 2.83
8 6 4 2 [ppm]
20 15 10 5 0

11

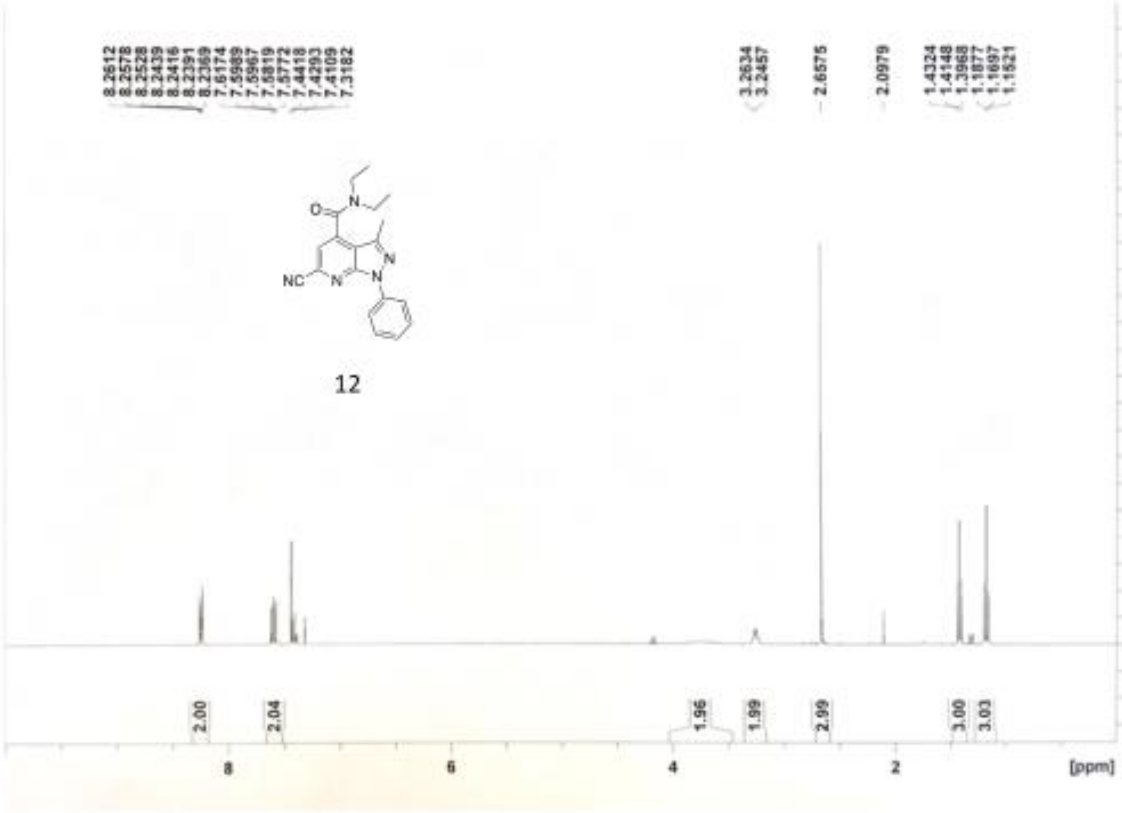
8.2612
8.2578
8.2528
8.2439
8.2416
8.2391
8.2369
7.6174
7.5989
7.5967
7.5819
7.5772
7.4418
7.4293
7.4109
7.3182
3.2634
3.2457
2.6575
2.0979
1.4324
1.4148
1.3968
1.1877
1.1697
1.1521
12
2.00
2.04
1.96
1.99
2.99
3.00
3.03
[ppm]

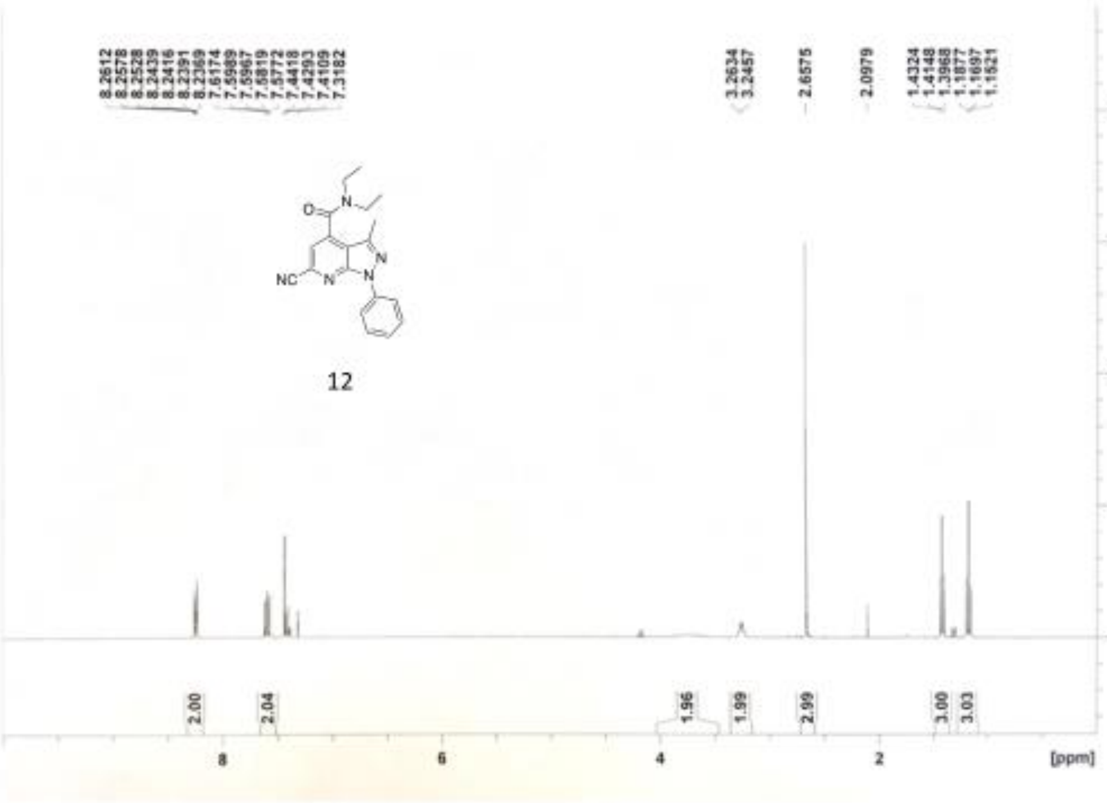
8.2612
8.2578
8.2528
8.2439
8.2416
8.2391
8.2369
7.6174
7.5989
7.5967
7.5819
7.5772
7.4418
7.4293
7.4109
7.3182
3.2634
3.2457
2.6575
2.0979
1.4324
1.4148
1.3968
1.1877
1.1697
1.1521
12
2.00
2.04
1.96
1.99
2.99
3.00
3.03
[ppm]

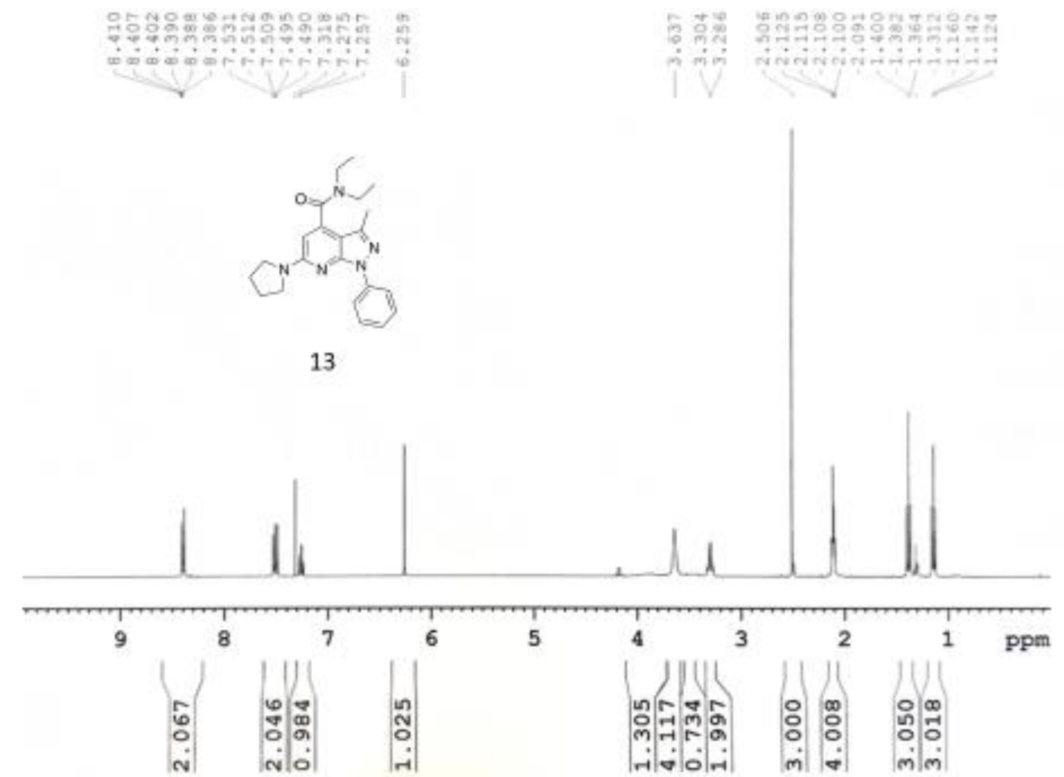
13
ppm

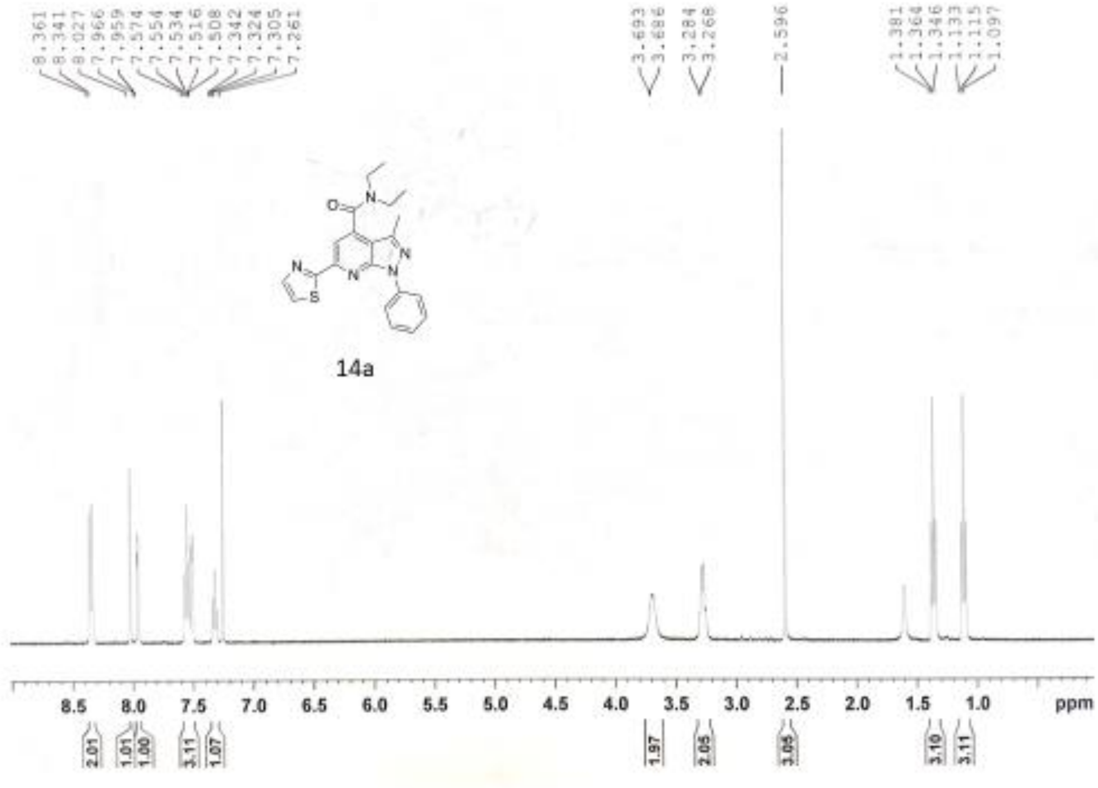
14a
ppm

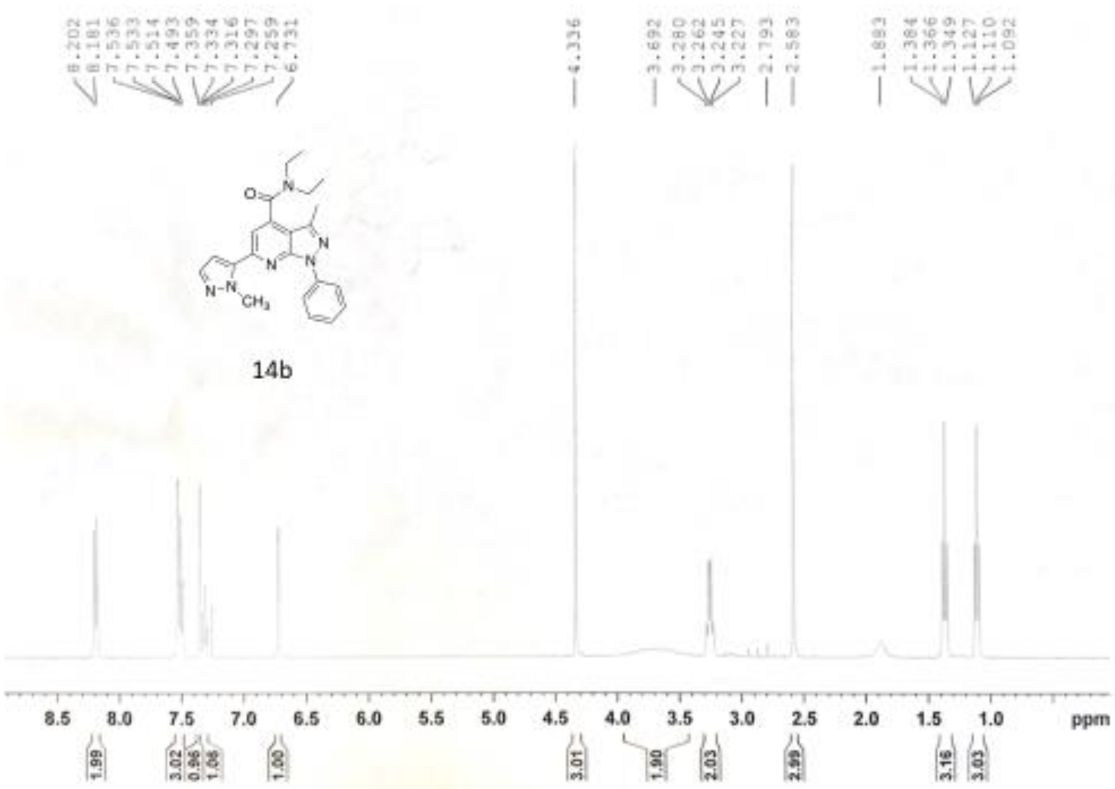
14b
ppm

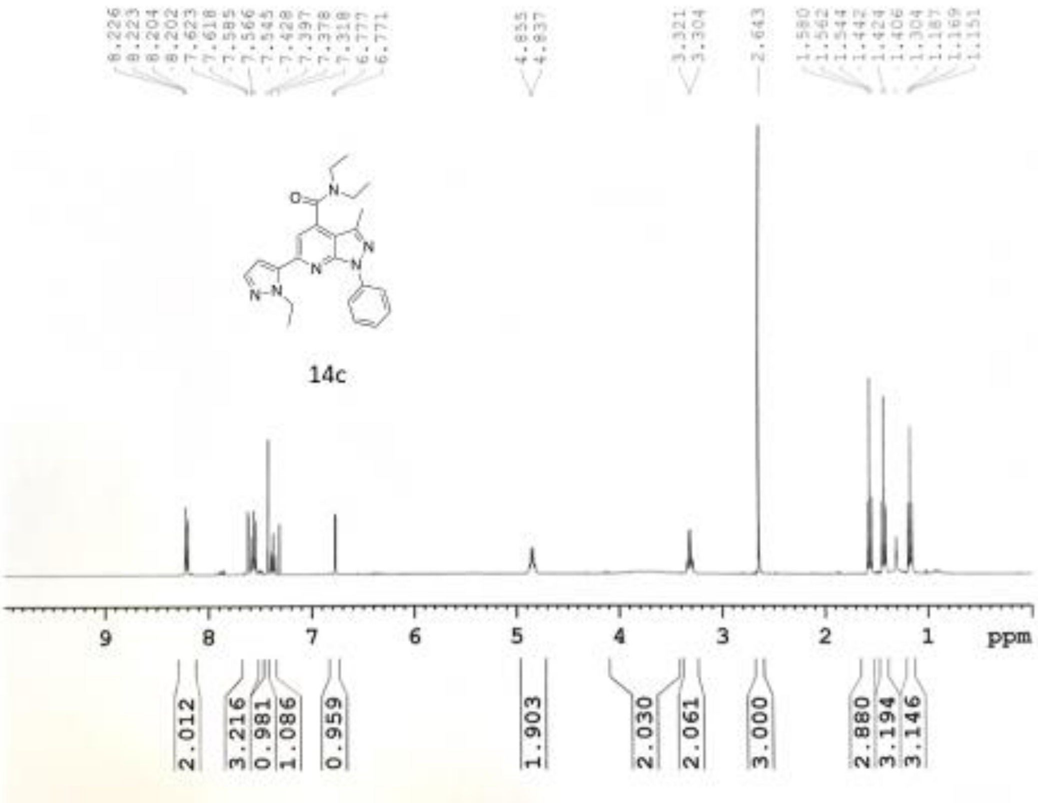
14c
ppm

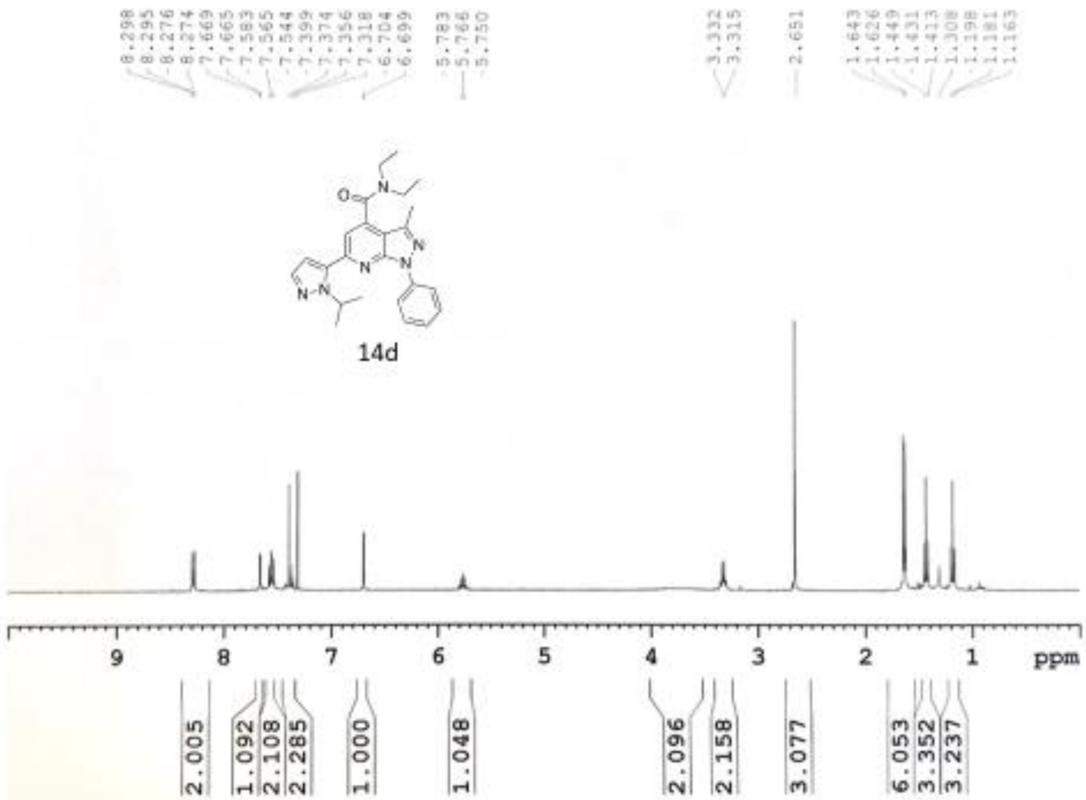
14d
ppm
2.005
1.092
2.108
2.285
1.000
1.048
2.096
2.158
3.077
6.053
3.352
3.237

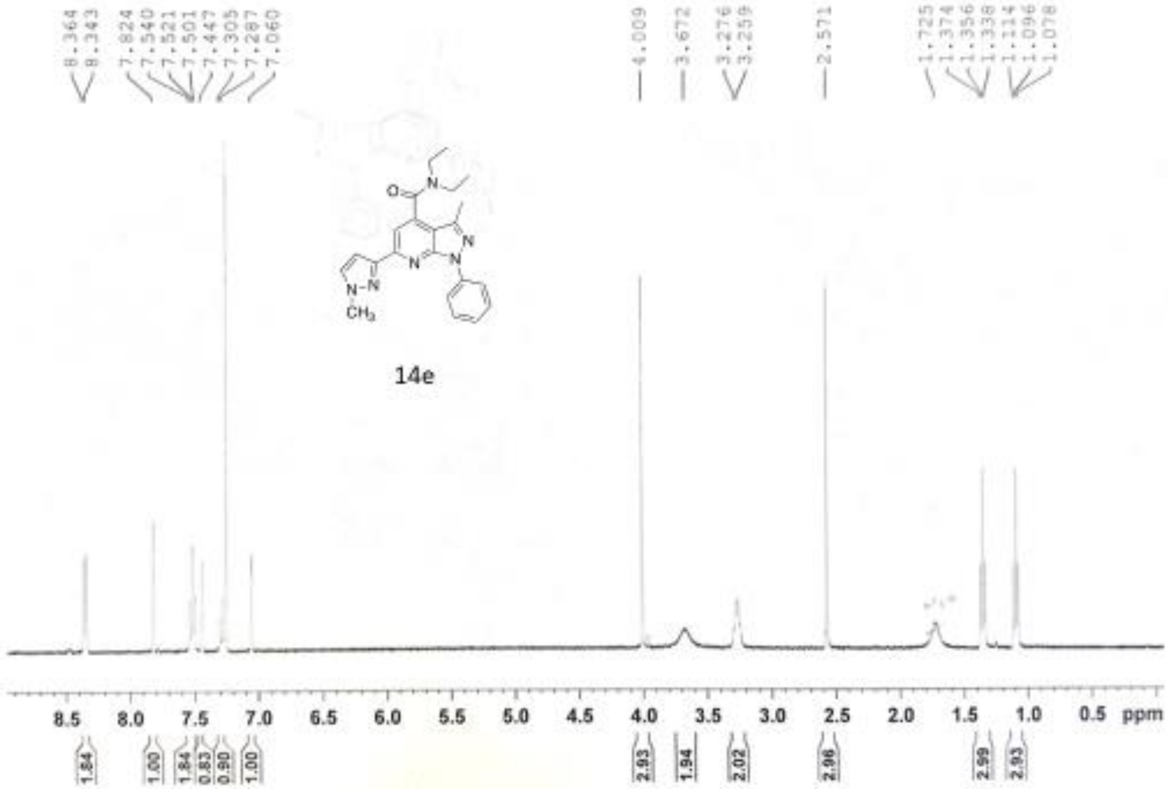
14e
ppm
1.84
1.00
1.84
0.83
0.90
1.00
2.93
1.94
2.02
2.96
2.99
2.93

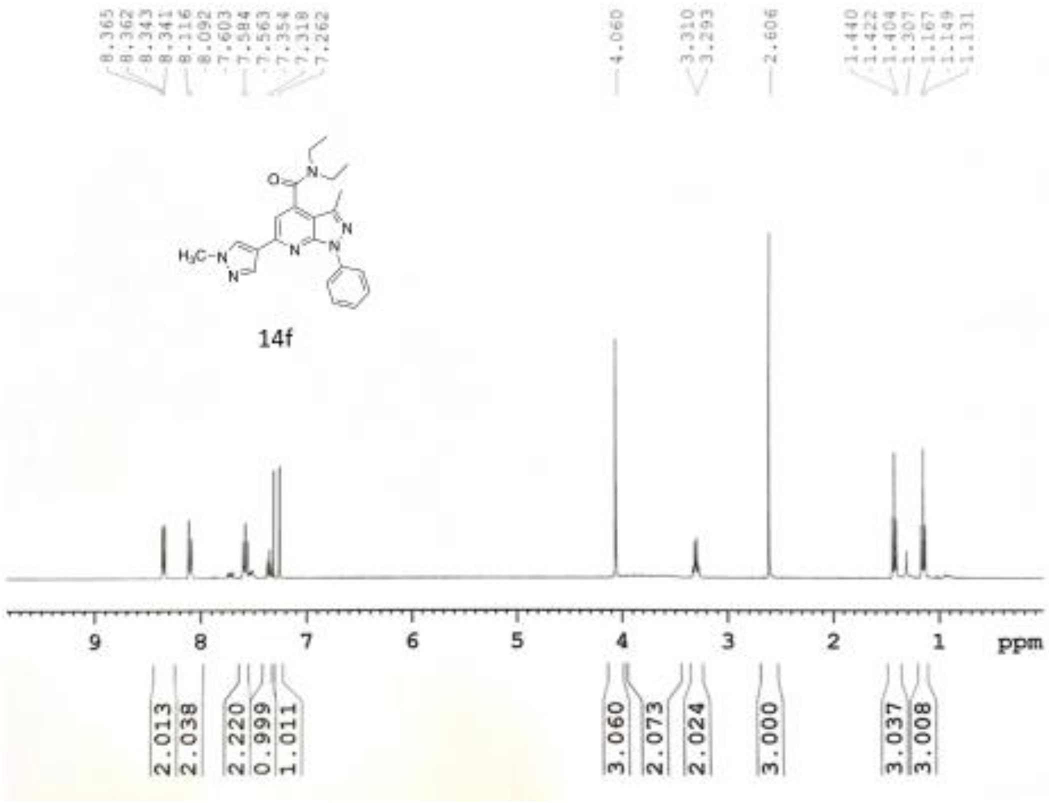
14f
ppm

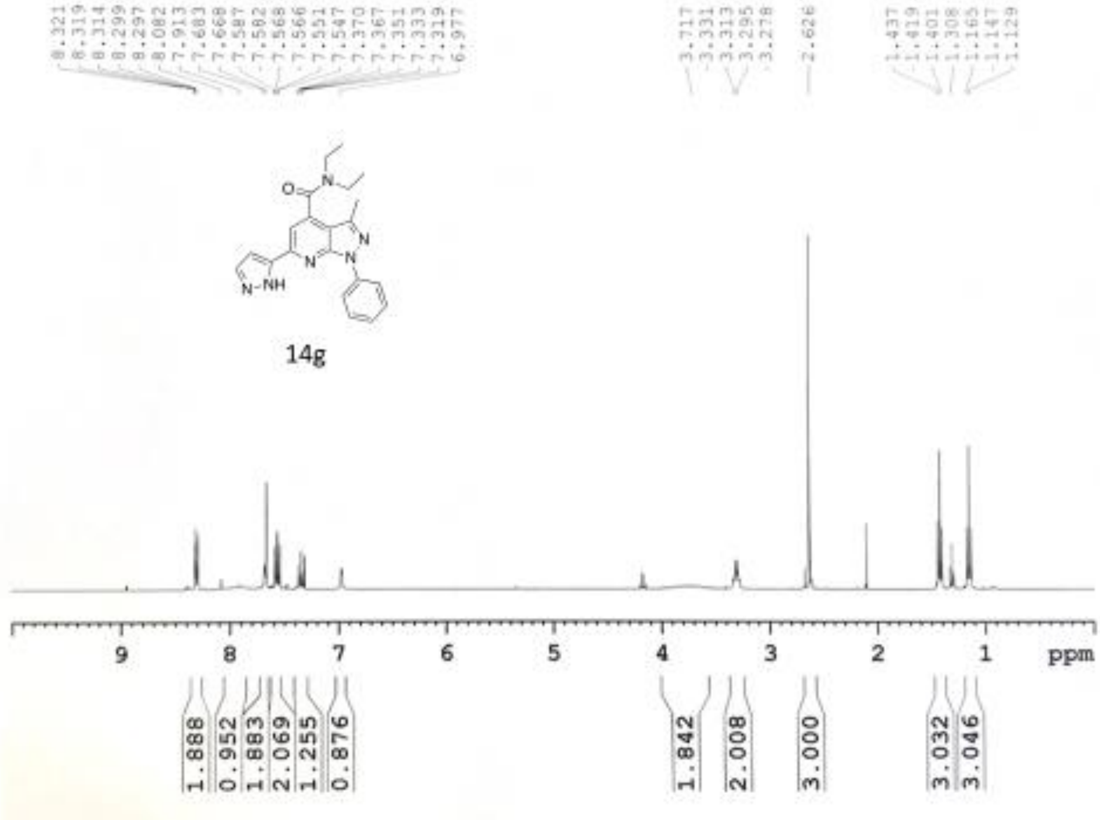
14g
ppm

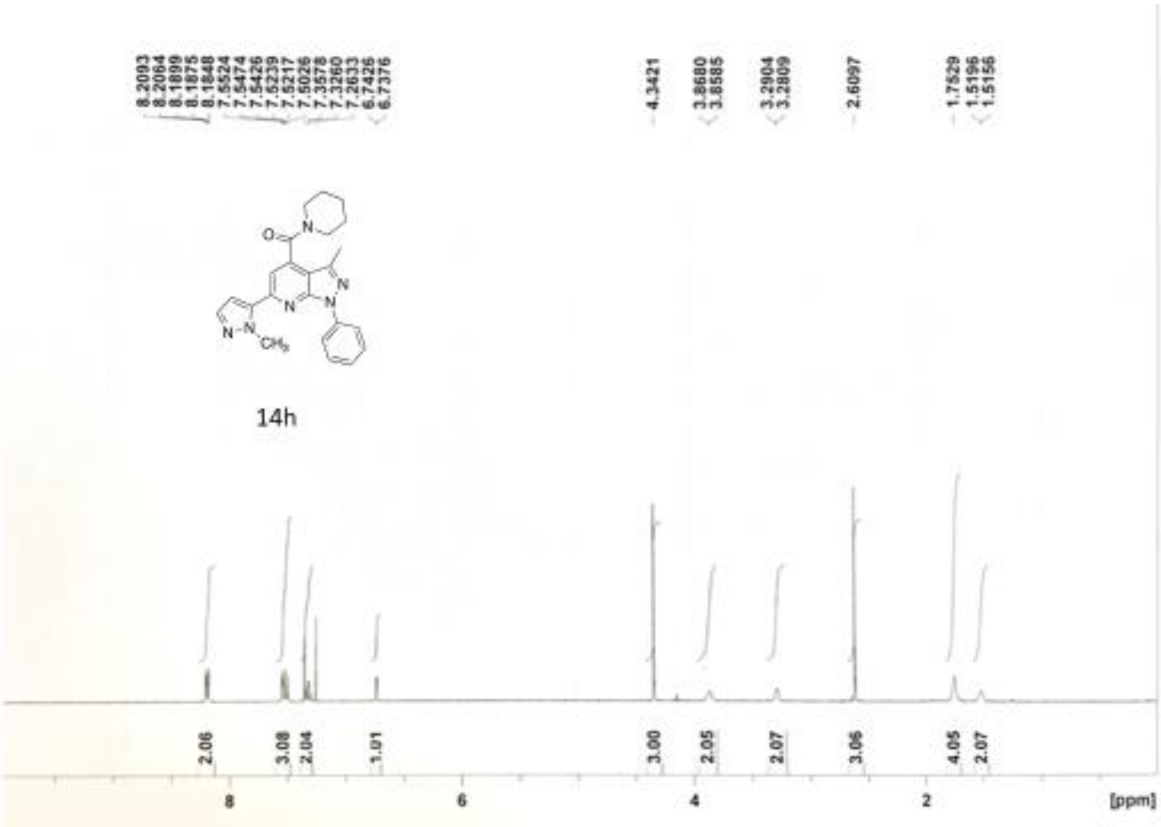
8.2093
8.2064
8.1899
8.1875
8.1848
7.5524
7.5474
7.5426
7.5239
7.5217
7.5026
7.3578
7.3260
7.2633
6.7426
6.7376
4.3421
3.8680
3.8585
3.2904
3.2809
2.6097
1.7529
1.5196
1.5156
N
O
N
N
N
N
N
CH3
14h
2.06
3.08
2.04
1.01
3.00
2.05
2.07
3.06
4.05
2.07
8
6
4
2
[ppm]

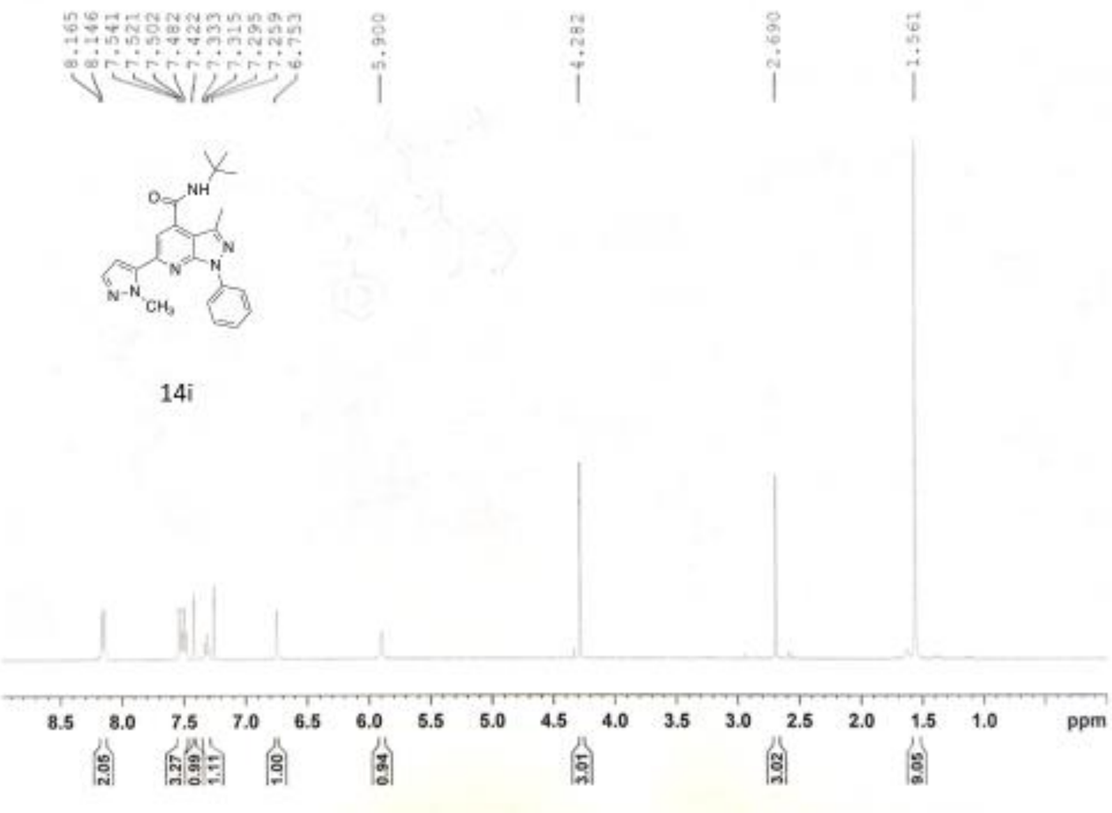
8.165
8.146
7.541
7.521
7.502
7.482
7.422
7.333
7.315
7.295
7.259
6.753
5.900
4.282
2.690
1.561
NH
O
N
N
N
N
CH3
14i
8.5
8.0
7.5
7.0
6.5
6.0
5.5
5.0
4.5
4.0
3.5
3.0
2.5
2.0
1.5
1.0
ppm
2.05
3.27
0.99
1.11
1.00
0.94
3.01
3.02
9.05

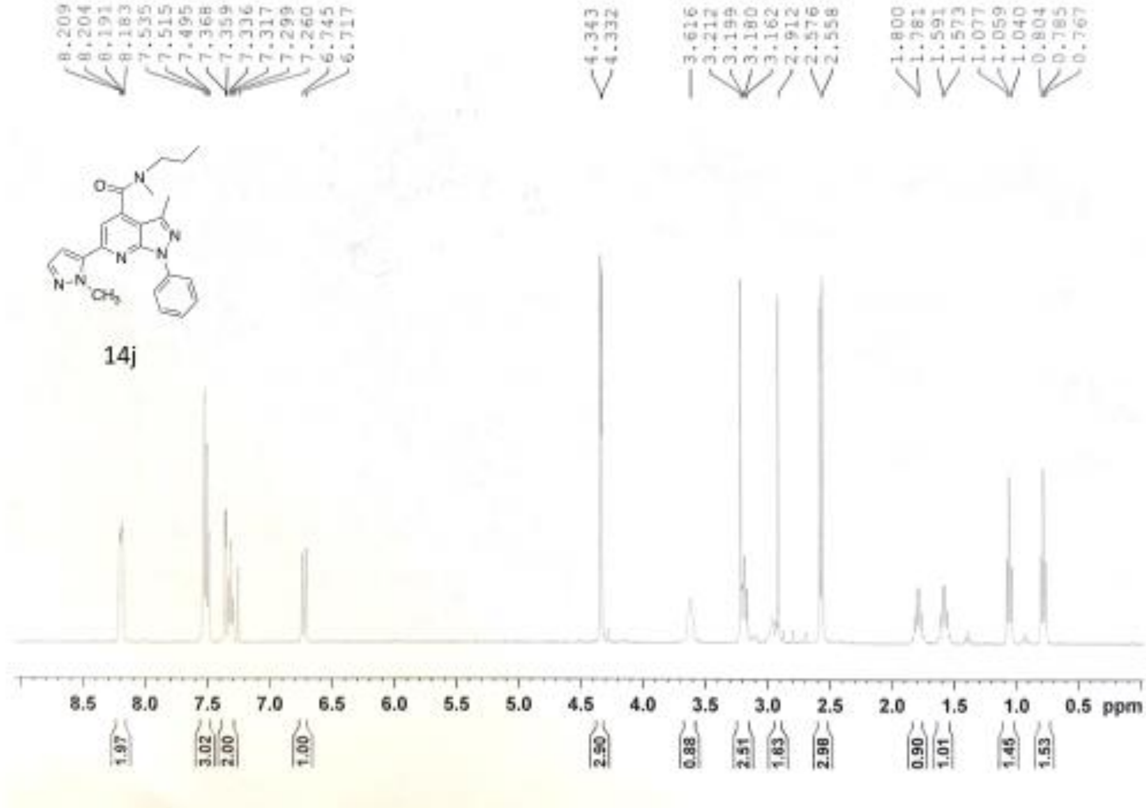
14j
ppm

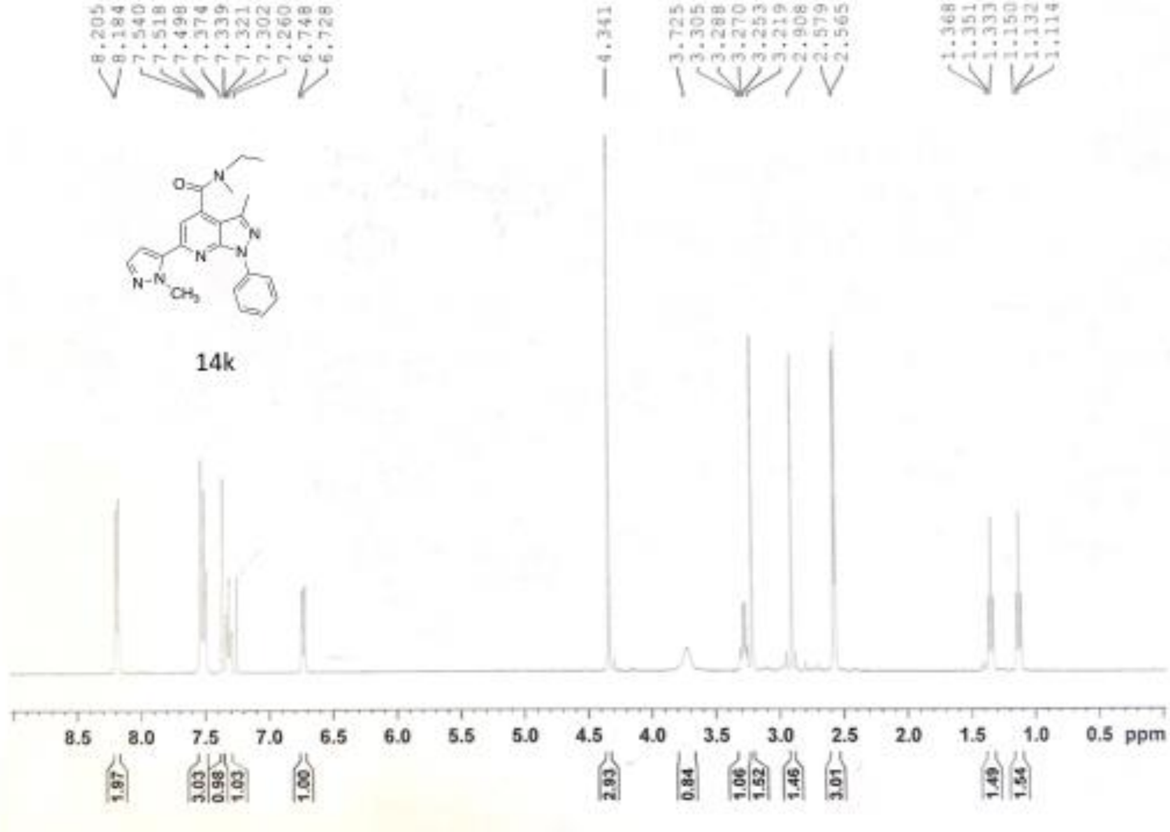
14k
ppm

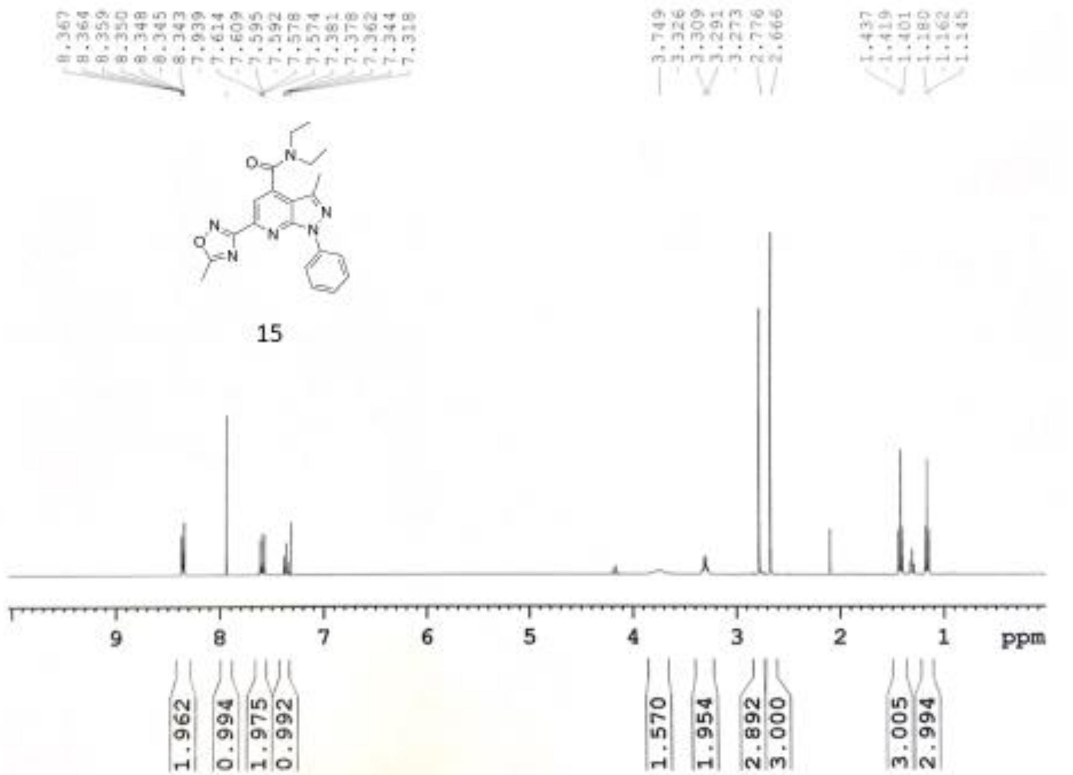
15
ppm
1.962
0.994
1.975
0.992
1.570
1.954
2.892
3.000
3.005
2.994

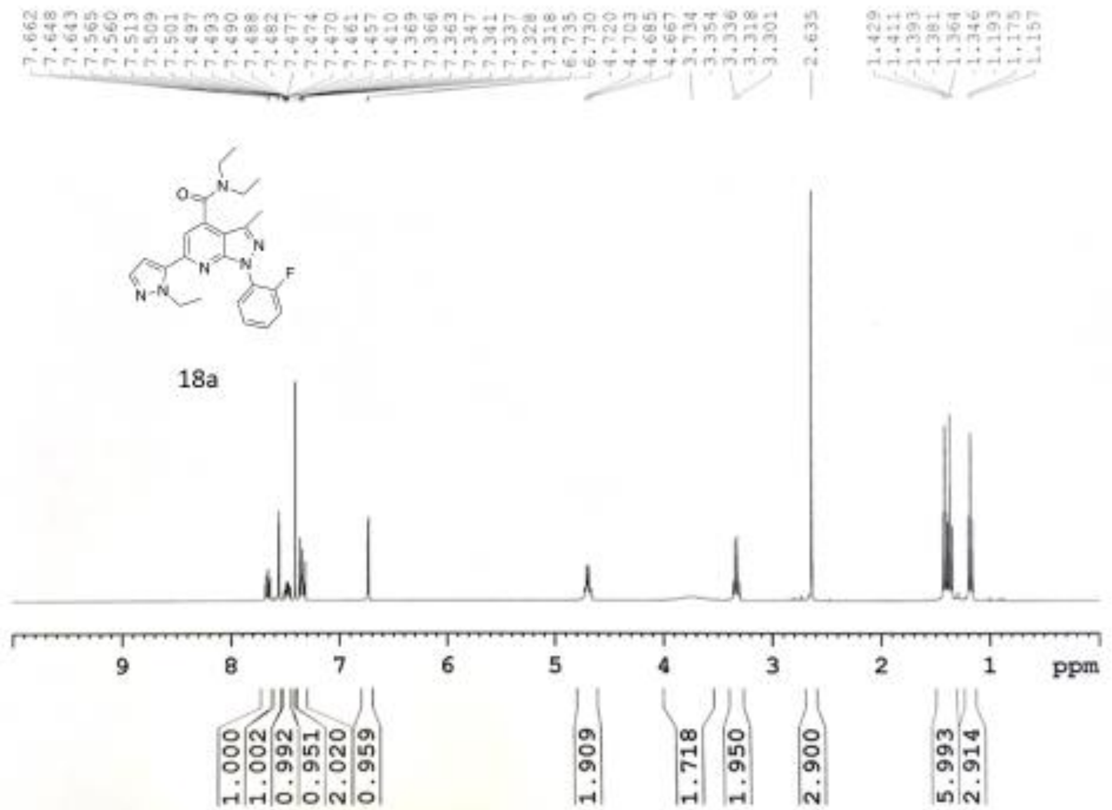
18a
ppm
1.000
1.002
0.992
0.951
2.020
0.959
1.909
1.718
1.950
2.900
5.993
2.914

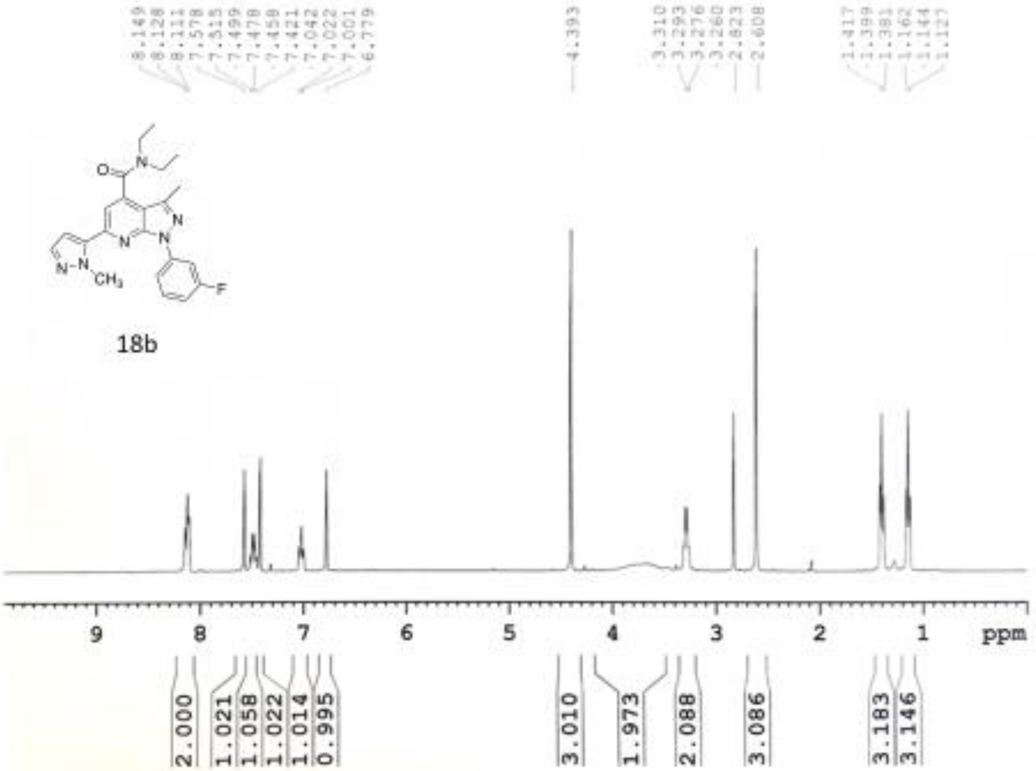
8.149
8.128
8.111
7.578
7.515
7.499
7.478
7.458
7.421
7.042
7.022
7.001
6.779
4.393
3.310
3.293
3.276
3.260
2.823
2.608
1.417
1.399
1.381
1.162
1.144
1.127
ppm
2.000
1.021
1.058
1.022
1.014
0.995
3.010
1.973
2.088
3.086
3.183
3.146

18b

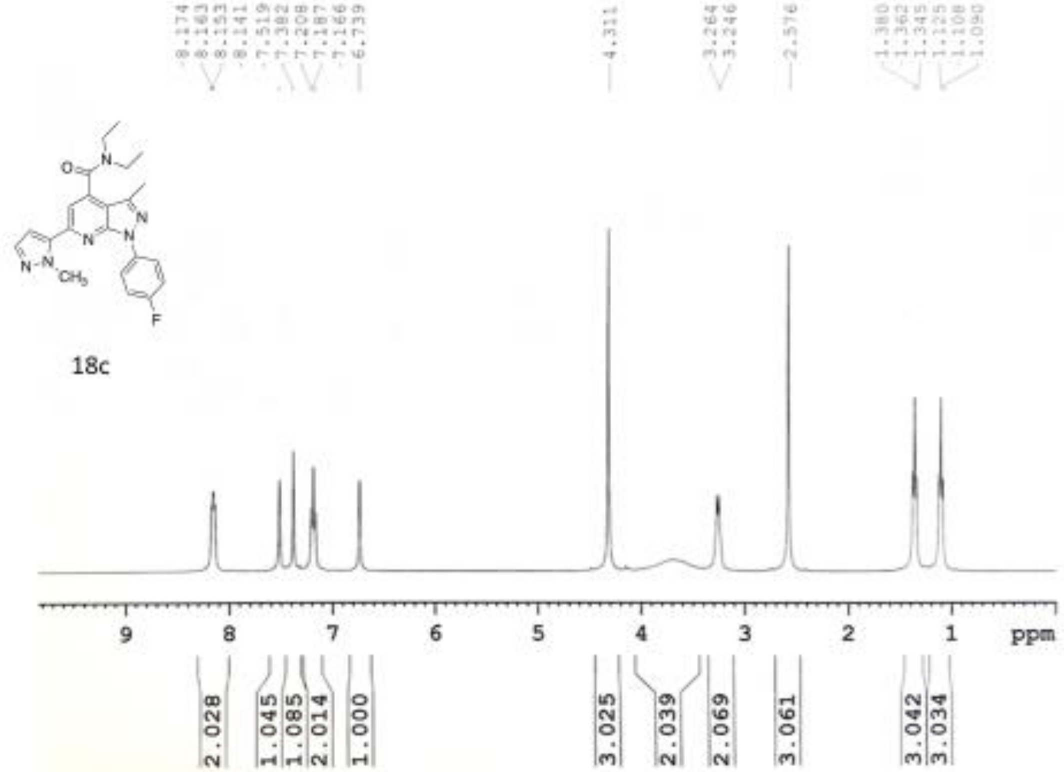
8.174
8.163
8.153
8.141
7.519
7.382
7.208
7.187
7.166
6.739
4.311
3.264
3.246
2.576
1.380
1.362
1.345
1.125
1.108
1.090
ppm
2.028
1.045
1.085
2.014
1.000
3.025
2.039
2.069
3.061
3.042
3.034

18c

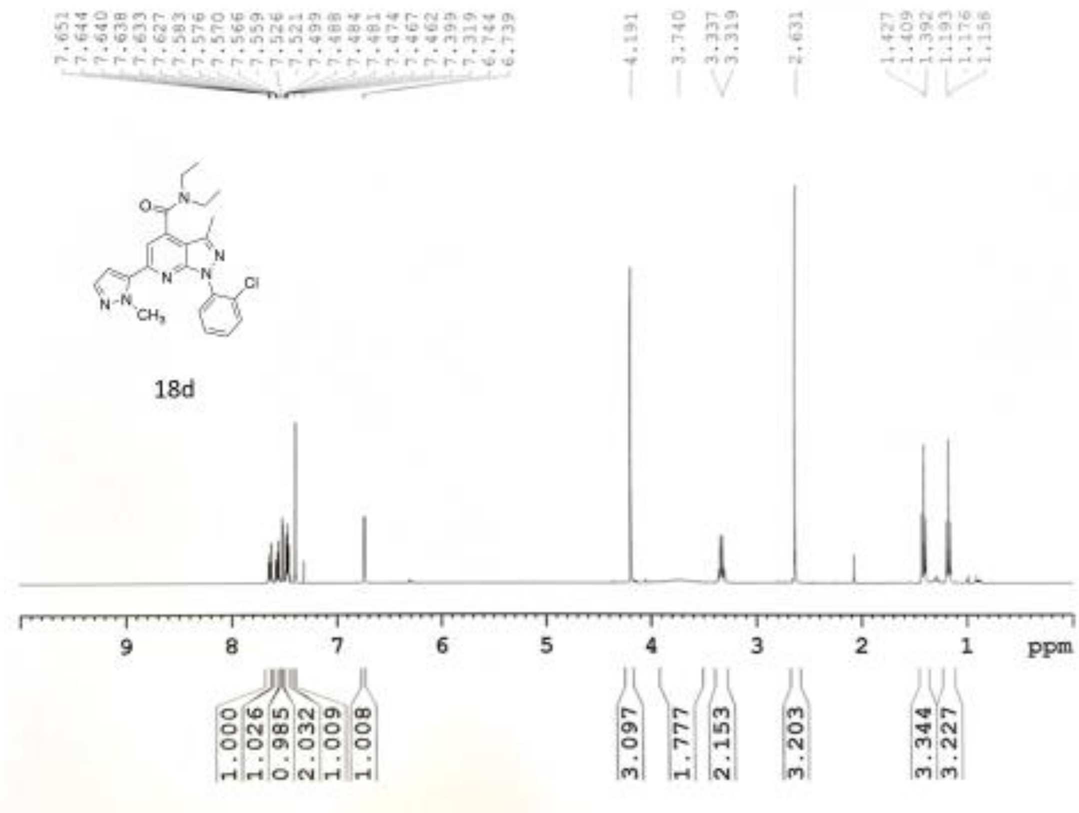
18d
ppm

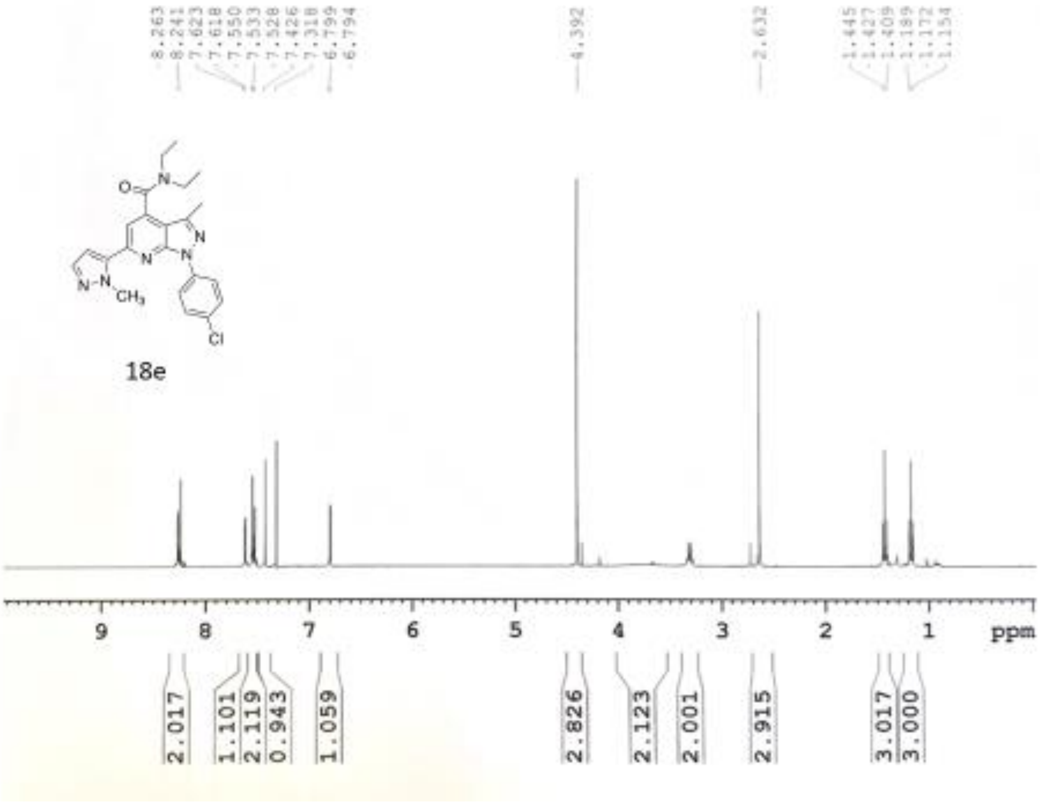
18e
ppm

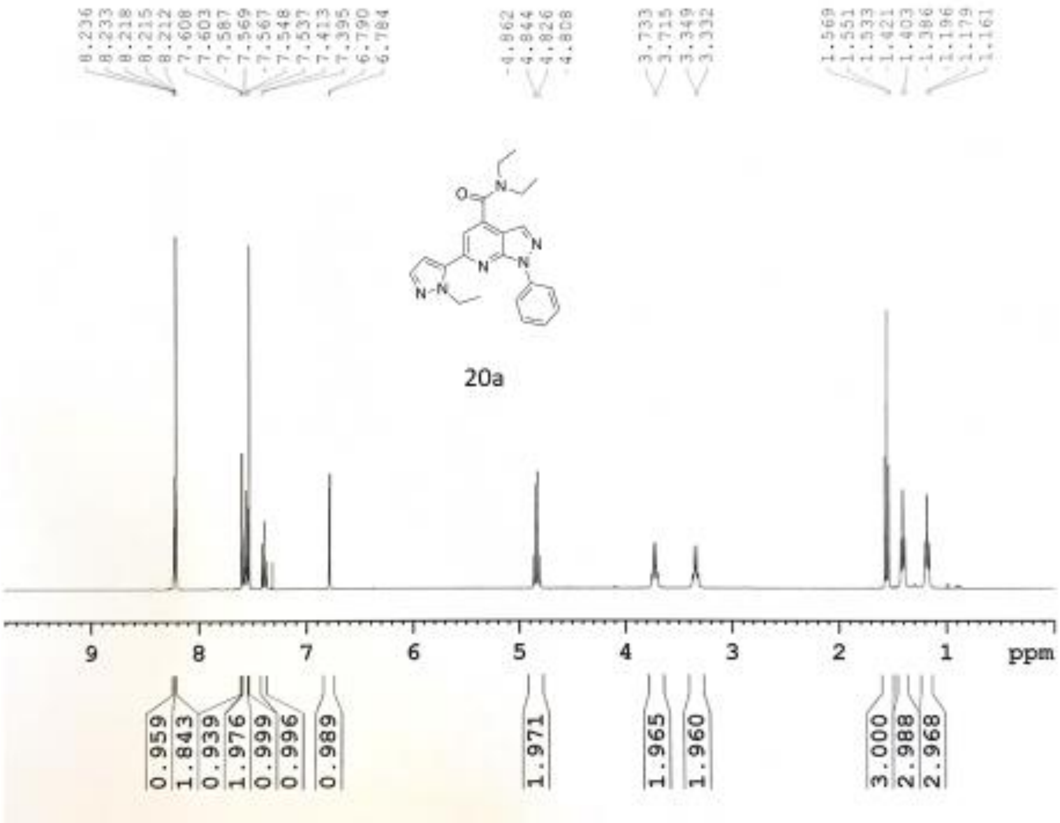

20a

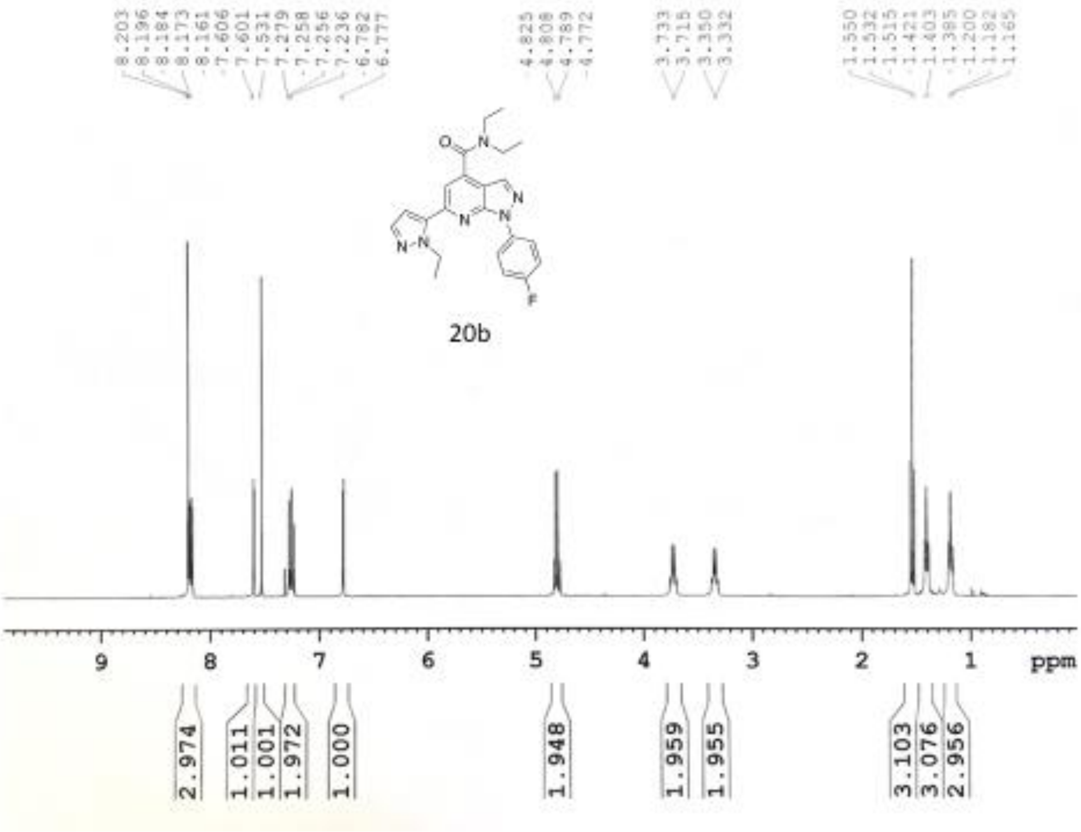

20b

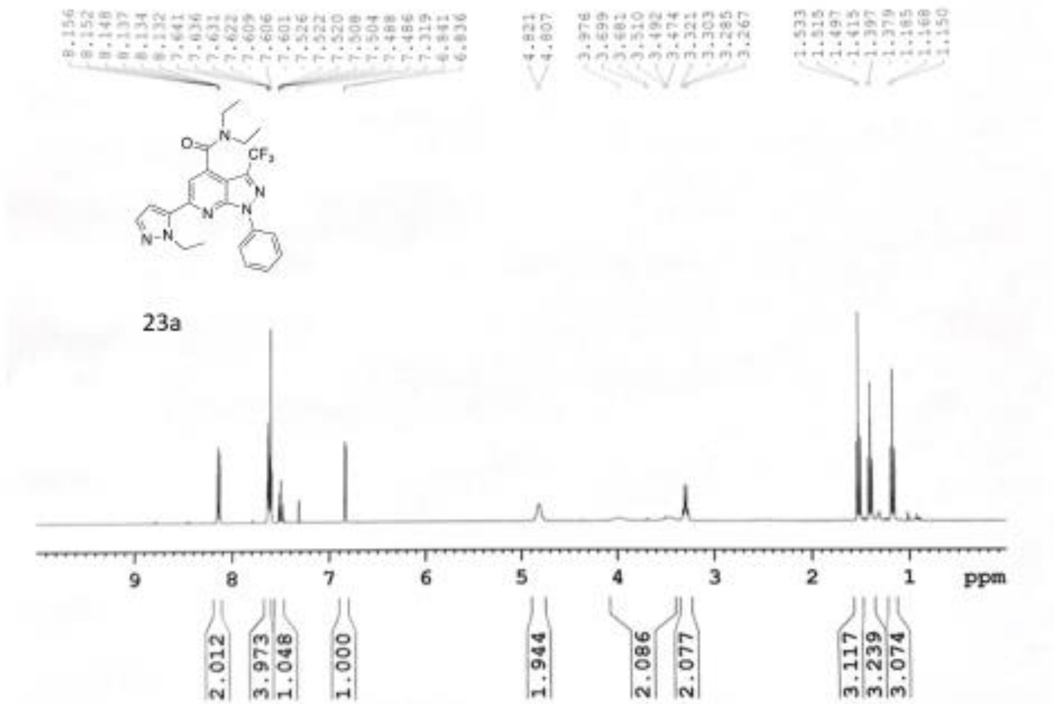

23a

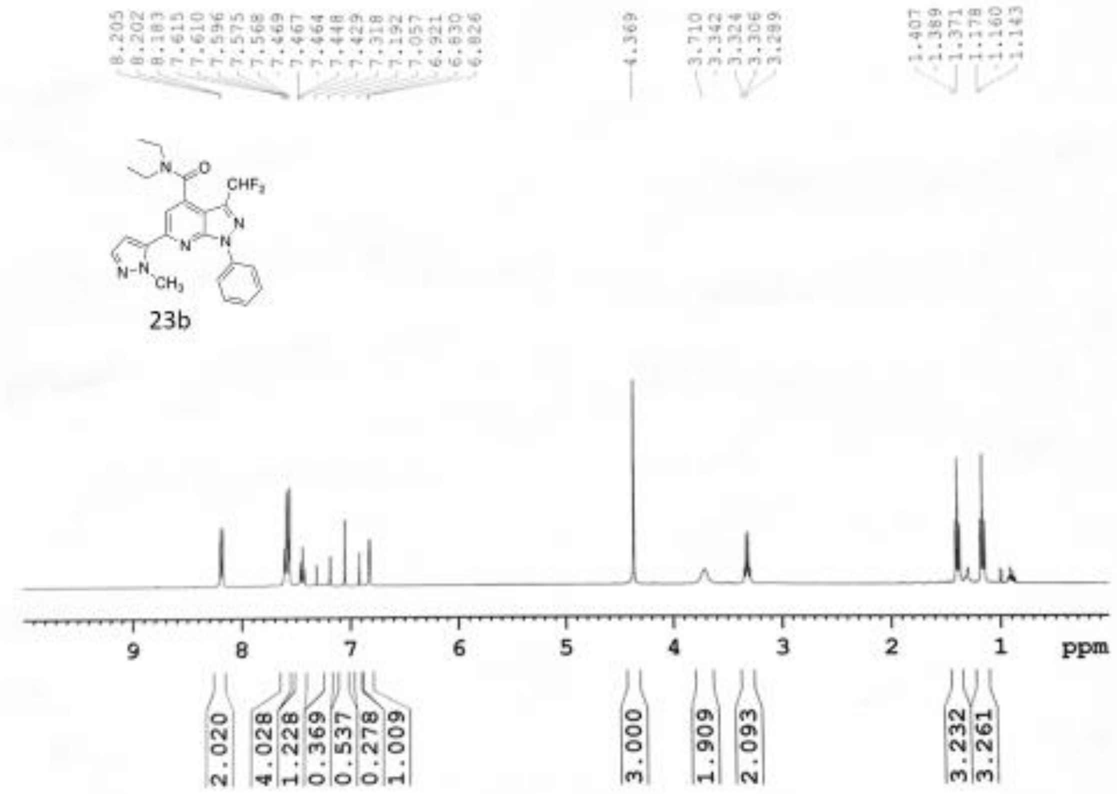

23b

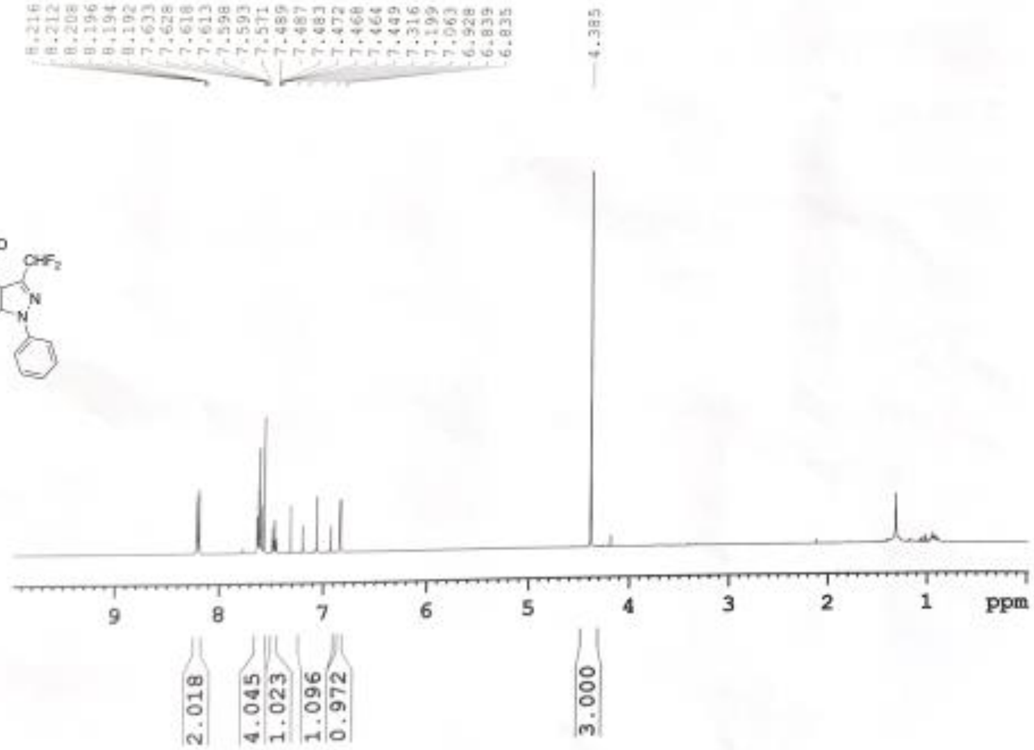

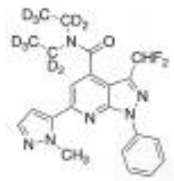

25

miniLC

Method: 6 MINUTE ISOCRATIC 80% acn
Batch: MLG
Sample: MSU-MLG-1-122
User: Administrator
S/N: 0118000000
Date: 11/16/2022 4:23 AM

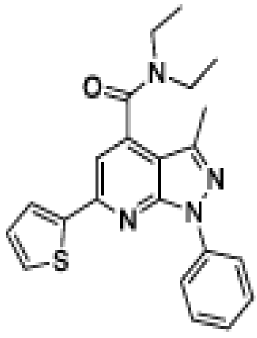

9a

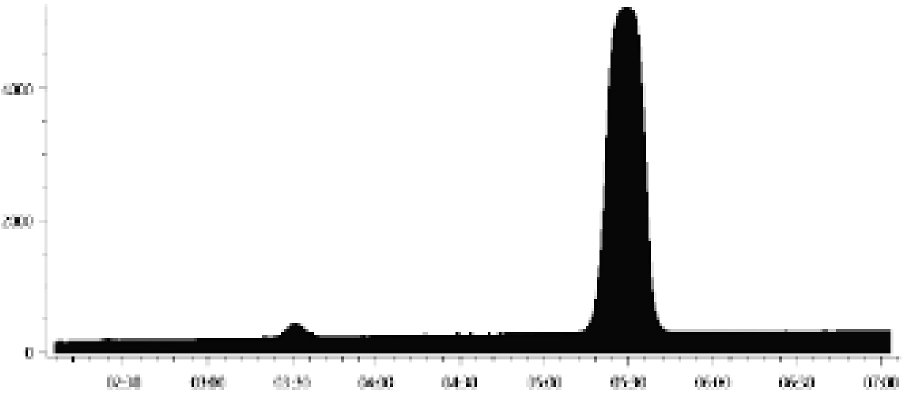

#	Compound	Retention Time	Area	% Area	Height
1		03:31.0	1320.8	1.8	189.14
2		05:29.5	72168.3	98.2	4937.01

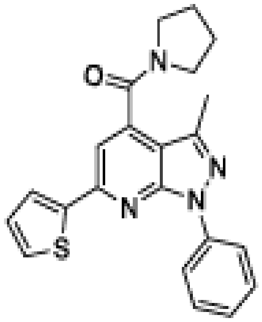

9b

miniLC

Method: 6 MINUTE ISOCRATIC 80% acn
Batch: RN
Sample: MSU-MLG-1-131
User: Administrator
S/N: 0118000000
Date: 11/16/2022 5:42 AM

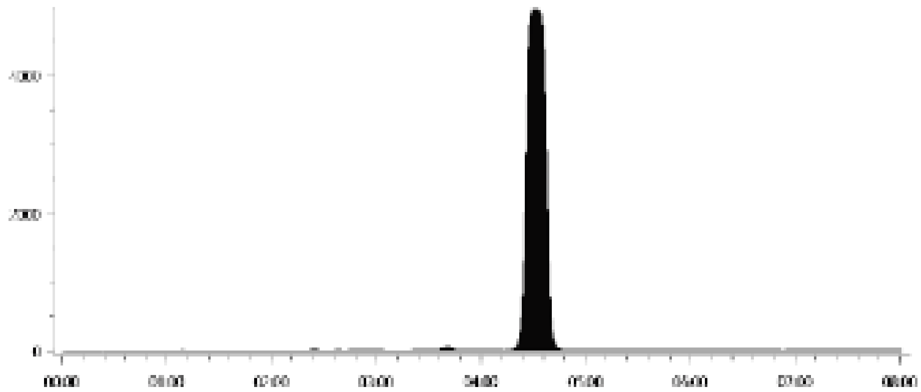

#	Compound	Retention Time	Area	% Area	Height
1		04:31.5	63515.5	100.0	4936.93

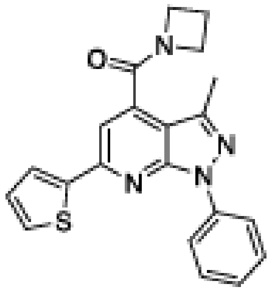

9c

miniLC

Method: 6 MINUTE ISOCRATIC 80% acn
Batch: LK
Sample: MSU-MLG-1-132
User: Guest
S/N: 0118000000
Date: 11/30/2022 4:39 AM

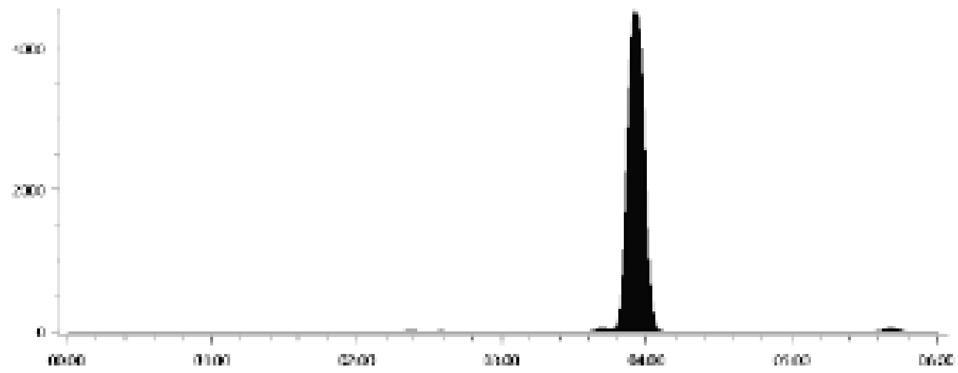

#	Compound	Retention Time	Area	% Area	Height
1		03:55.5	37985.3	98.6	4519.82
2		05:41.0	492.1	1.4	61.12

miniLC

Method: 6 MINUTE ISOCRATIC 80% acn
Batch: MLG
Sample: MSU-MLG-1-133
User: Administrator
S/N: 0118000000
Date: 11/16/2022 4:51 AM

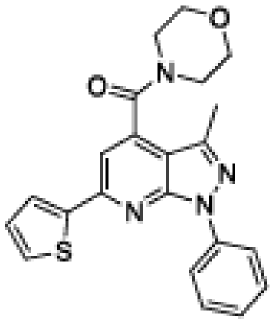

9d

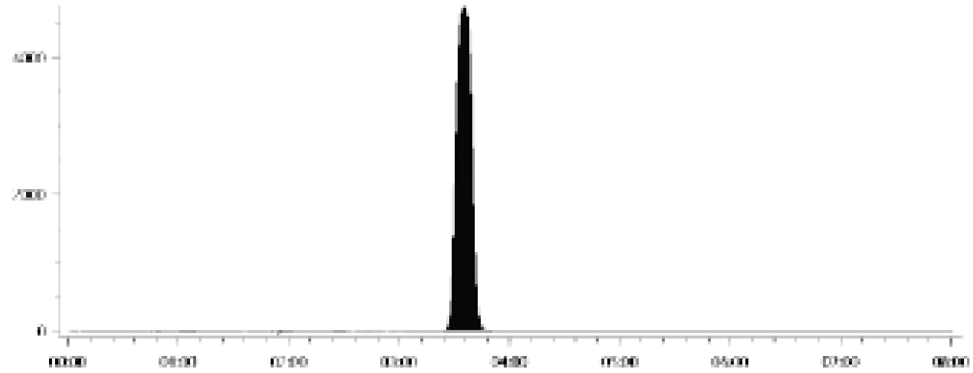

#	Compound	Retention Time	Area	% Area	Height
1		03:35.5	45196.0	100.0	4751.95

miniLC

Method: 6 MINUTE ISOCRATIC 80% acn
Batch: nnn
Sample: MSU-MLG-1-136
User: Administrator
S/N: 0118000000
Date: 11/16/2022 3:59 AM

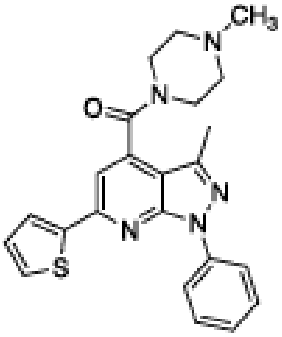

9e

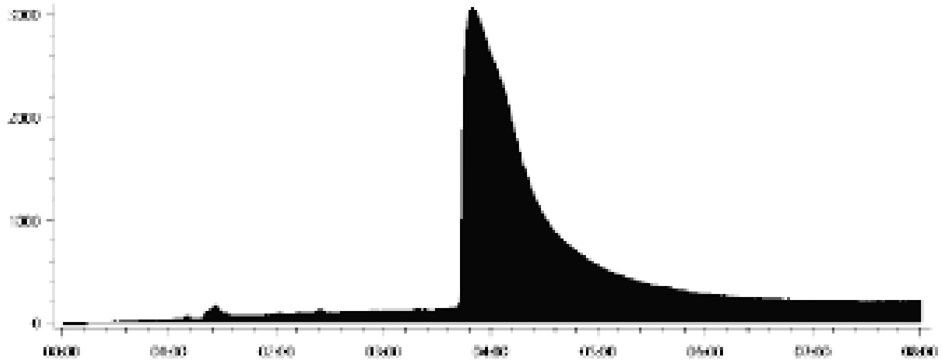

#	Compound	Retention Time	Area	% Area	Height
1		03:50.5	139138.0	100.0	2961.82

miniLC

Method: 6 MINUTE ISOCRATIC 80% acn
Batch: BN
Sample: MSU-MLG-1-121
User: Administrator
S/N: 0118000000
Date: 11/16/2022 5:32 AM

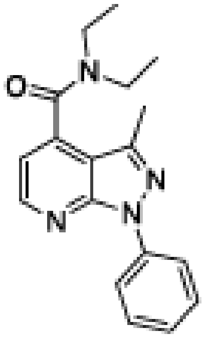

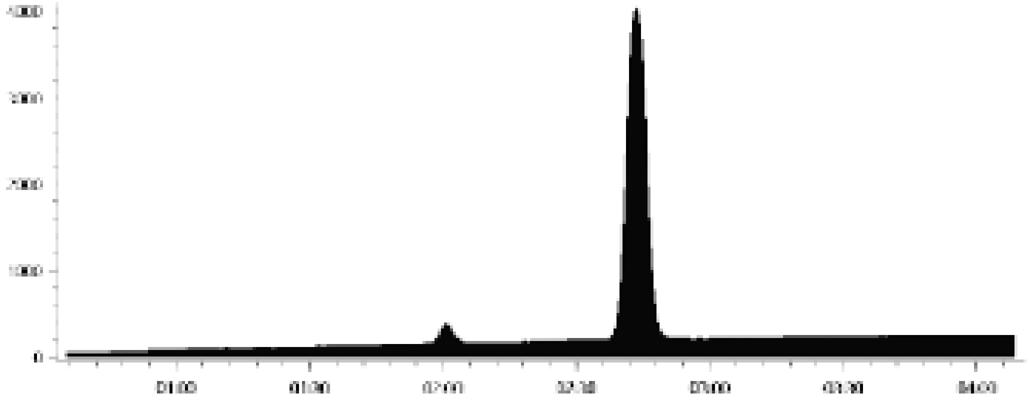

11

#	Compound	Retention Time	Area	% Area	Height
1		02:01.0	748.7	3.8	229.40
2		02:43.5	19088.3	96.2	3840.73

miniLC

Method: 6 MINUTE ISOCRATIC 80% acn
Batch: BNN
Sample: MSU-TSR-1-32
User: Guest
S/N: 0118000000
Date: 11/30/2022 12:52 AM

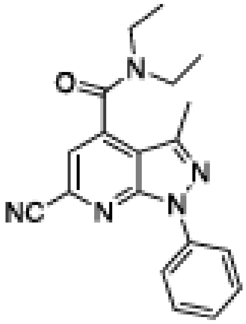

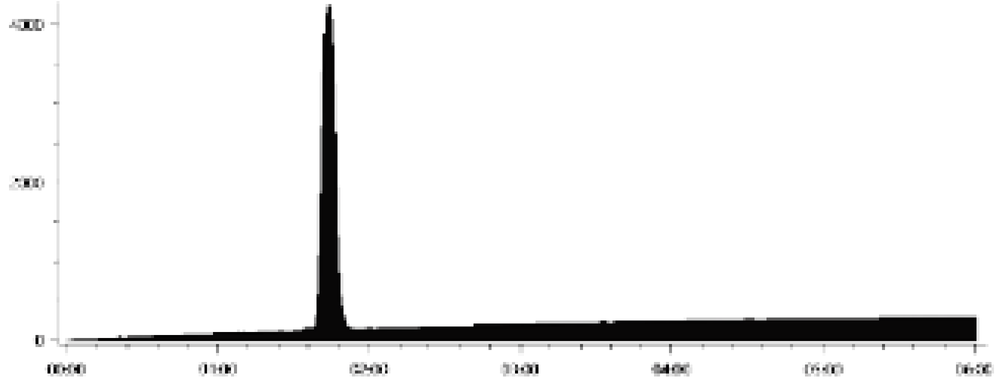

12

#	Compound	Retention Time	Area	% Area	Height
1		01:44.0	37339.5	100.0	4108.69

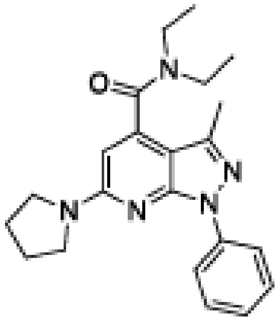

13

miniLC

Method: 6 MINUTE ISOCRATIC 80% acn
Batch: UY
Sample: MSU-TSR-1-62
User: Guest
S/N: 0118000000
Date: 11/30/2022 12:06 AM

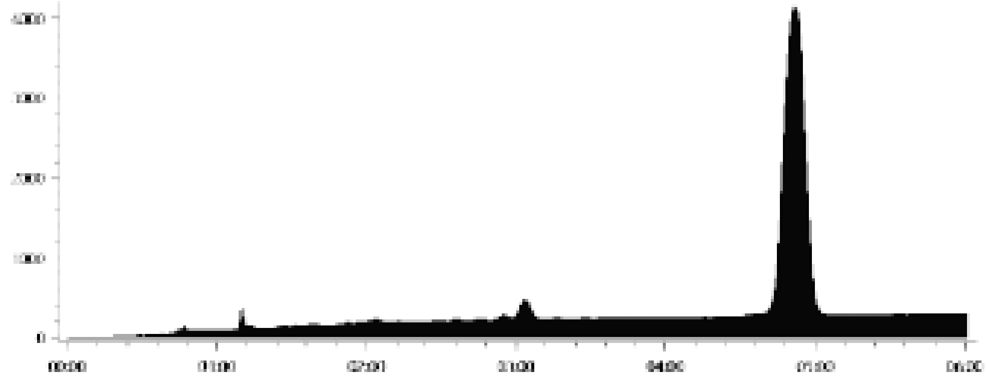

#	Compound	Retention Time	Area	% Area	Height
1		01:12.0	396.6	1.1	50.53
2		03:03.5	1016.7	2.7	231.62
3		04:52.0	35902.2	96.2	3874.36

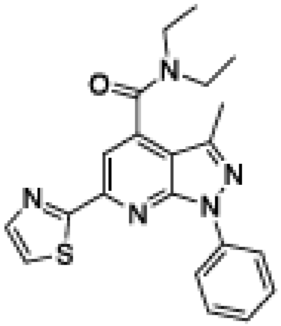

14a

miniLC

Method: 6 MINUTE ISOCRATIC 80% acn
Batch: MLG-SECOND
Sample: MSU-MLG-1-169
User: Administrator
S/N: 0118000000
Date: 11/16/2022 11:59 PM

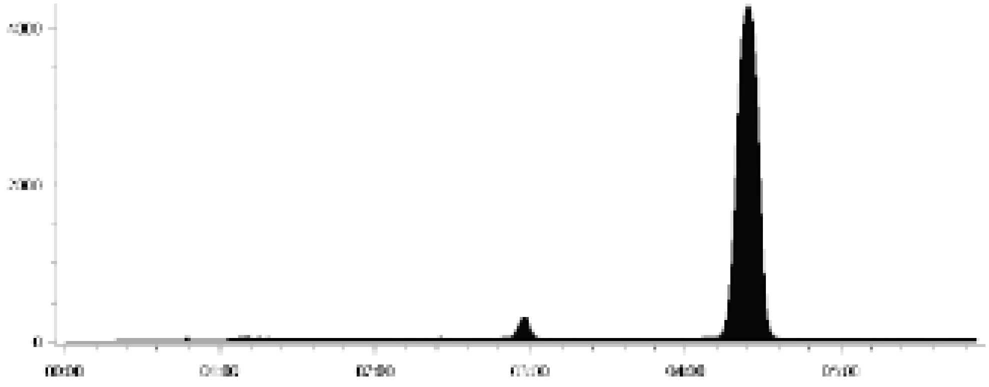

#	Compound	Retention Time	Area	% Area	Height
1		02:57.5	4367.1	3.3	285.64
2		04:24.0	39638.6	96.7	4243.10

miniLC

Method: 6 MINUTE ISOCRATIC 80% acn
Batch: LK
Sample: MSU-MLG-1-185
User: Guest
S/N: 0118000000
Date: 11/30/2022 4:47 AM

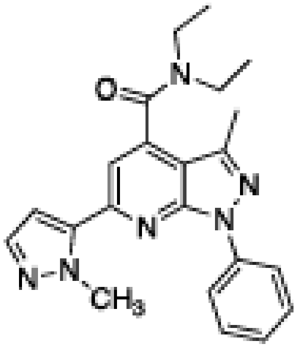

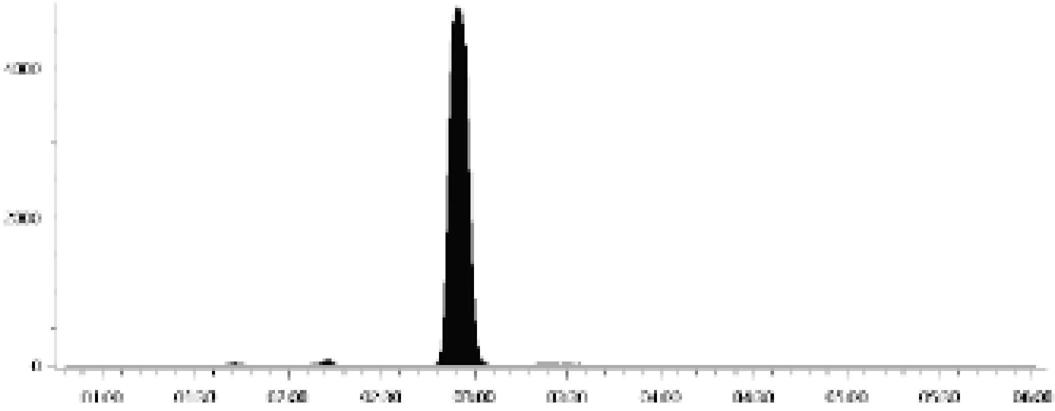

14b

#	Compound	Retention Time	Area	% Area	Height
1		02:12.5	347.6	1.0	79.46
2		02:55.0	32596.9	99.0	4797.49

miniLC

Method: 6 MINUTE ISOCRATIC 80% acn
Batch: LK
Sample: MSU-TSR-1-57
User: Guest
S/N: 0118000000
Date: 11/30/2022 4:24 AM

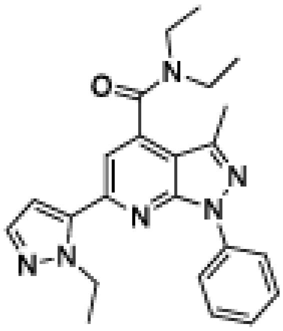

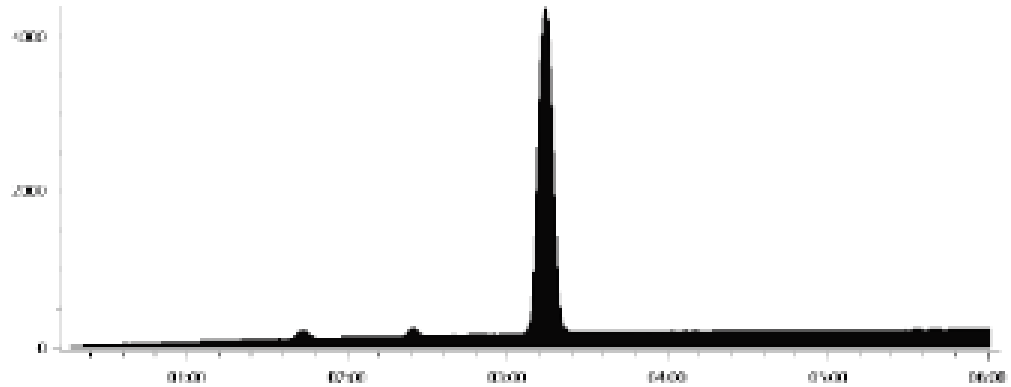

14c

#	Compound	Retention Time	Area	% Area	Height
1		01:44.0	472.8	1.7	95.39
2		02:24.5	337.0	1.2	68.37
3		03:14.5	27391.2	97.0	4140.54

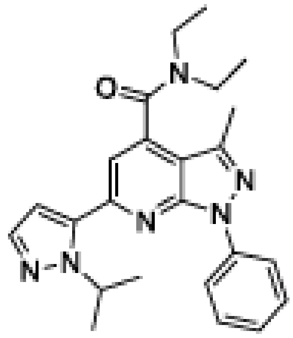

14d

miniLC

Method: 6 MINUTE ISOCRATIC 80% acn
Batch: KJ
Sample: MSU-TSR-1-54
User: Administrator
S/N: 0118000000
Date: 11/29/2022 1:04 AM

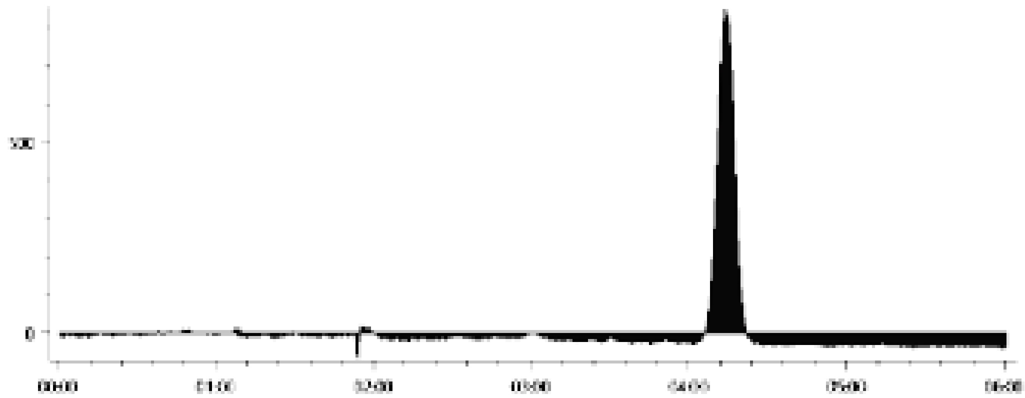

#	Compound	Retention Time	Area	% Area	Height
1		04:14.0	8133.1	100.0	863.94

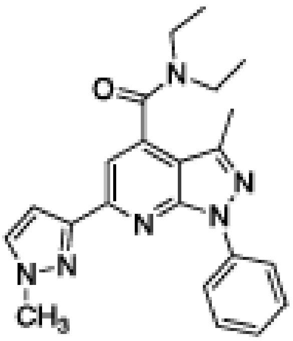

14e

miniLC

Method: 6 MINUTE ISOCRATIC 80% acn
Batch: LK
Sample: MSU-MLG-1-189
User: Guest
S/N: 0118000000
Date: 11/30/2022 4:31 AM

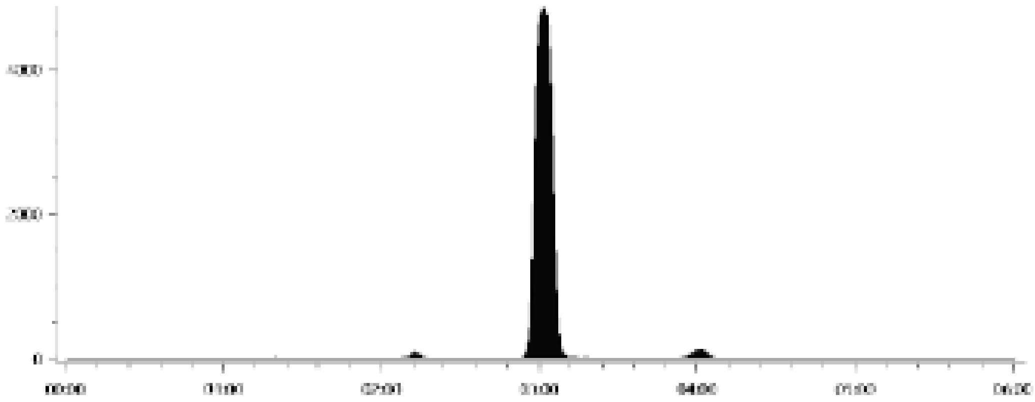

#	Compound	Retention Time	Area	% Area	Height
1		02:13.0	816.6	1.0	95.65
2		03:01.5	35370.5	96.7	4817.22
3		04:00.5	800.4	2.3	131.12

miniLC

Method: 6 MINUTE ISOCRATIC 80% acn
Batch: UY
Sample: MSU-FSH-1-55
User: Guest
S/N: 0118000000
Date: 11/30/2022 12:16 AM

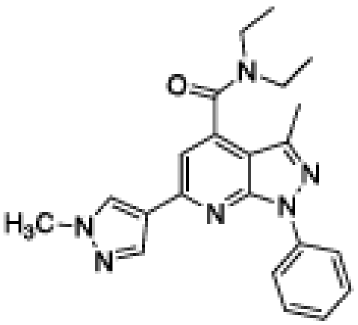

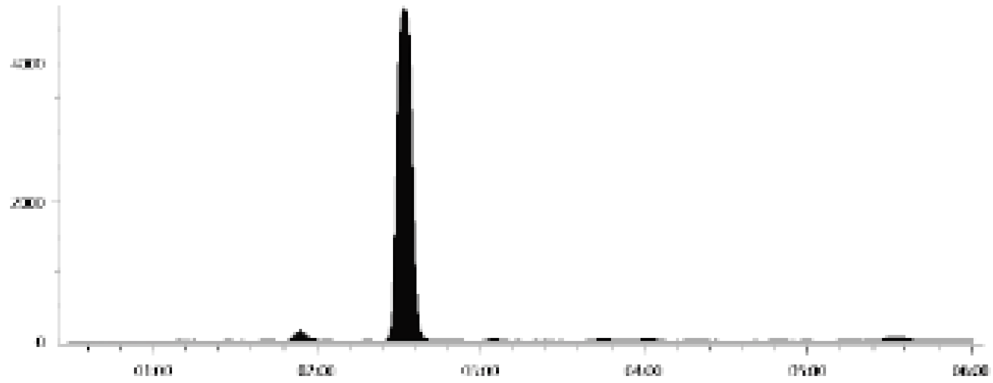

14f

#	Compound	Retention Time	Area	% Area	Height
1		01:54.5	545.1	1.8	125.34
2		02:32.0	30351.4	96.8	4796.92
3		05:32.5	426.0	1.4	51.36

miniLC

Method: 6 MINUTE ISOCRATIC 80% acn
Batch: K
Sample: MSU-SMG-2-41
User: Guest
S/N: 0118000000
Date: 11/12/2022 12:13 AM

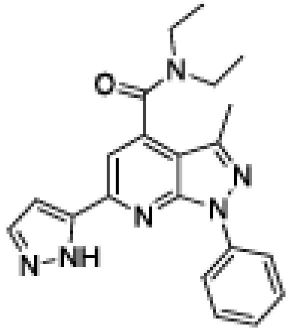

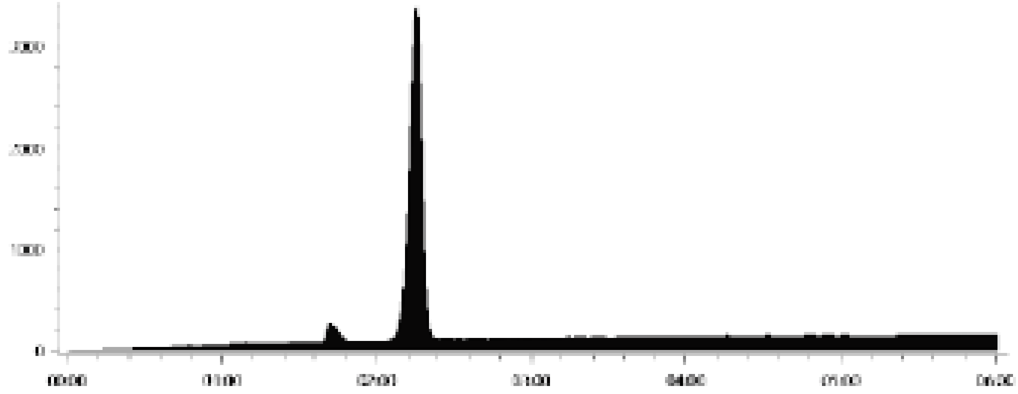

14g

#	Compound	Retention Time	Area	% Area	Height
1		01:43.0	784.0	3.7	194.81
2		02:15.5	19772.4	96.3	3252.82

miniLC

Method: 6 MINUTE ISOCRATIC 80% acn
Batch: UY
Sample: MSU-TSR-1-3
User: Guest
S/N: 0118000000
Date: 11/30/2022 12:31 AM

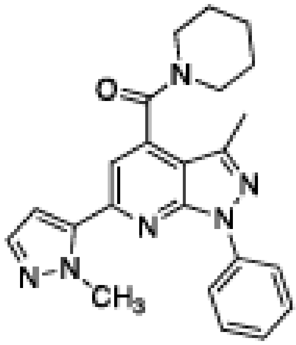

14h

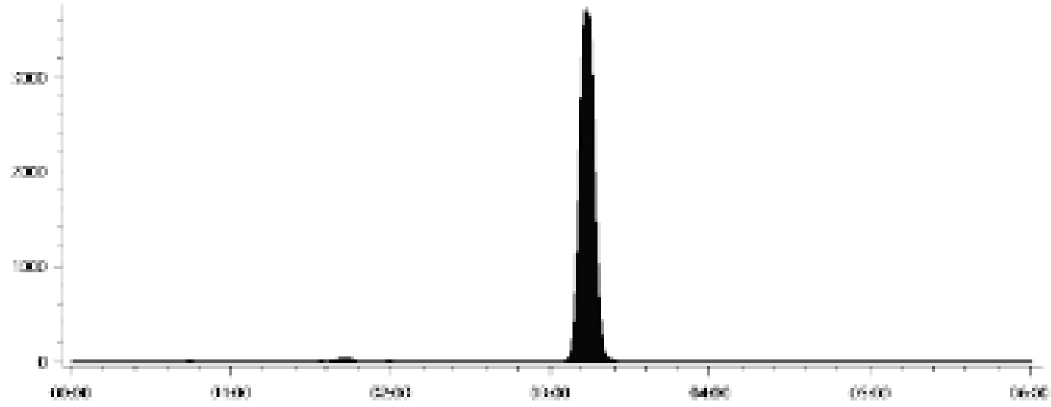

#	Compound	Retention Time	Area	% Area	Height
1		03:14.0	225[illegible]1.2	100.0	3724.79

miniLC

Method: 6 MINUTE ISOCRATIC 80% acn
Batch: JH
Sample: MSU-MLG-1-186
User: Administrator
S/N: 0118000000
Date: 11/18/2022 11:48 PM

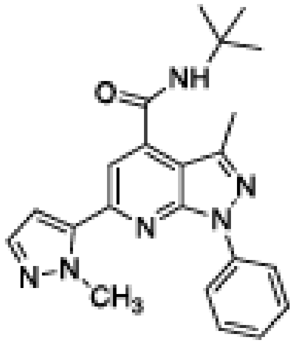

14i

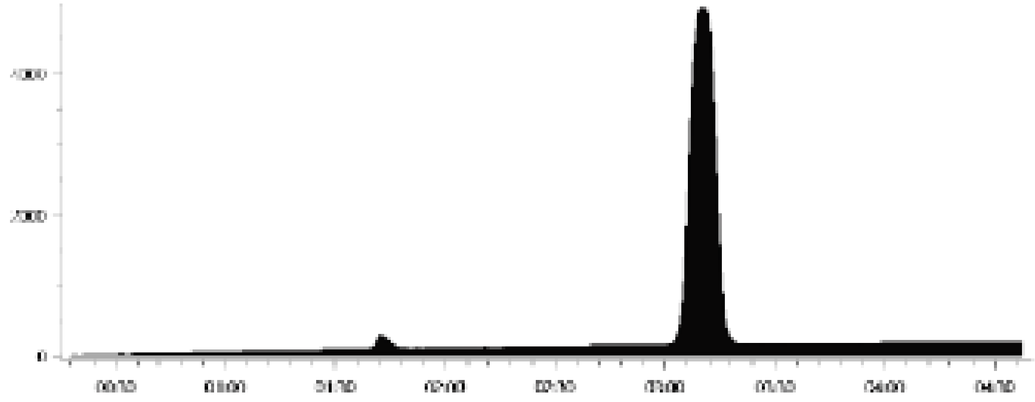

#	Compound	Retention Time	Area	% Area	Height
1		01:43.5	762.2	2.1	151.69
2		03:10.5	36349.9	97.9	4773.29

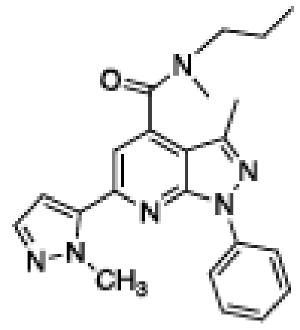

14j

miniLC

Method: 6 MINUTE ISOCRATIC 80% acn
Batch: MLG-SECOND
Sample: MSU-MLG-1-198
User: Administrator
S/N: 0118000000
Date: 11/17/2022 12:30 AM

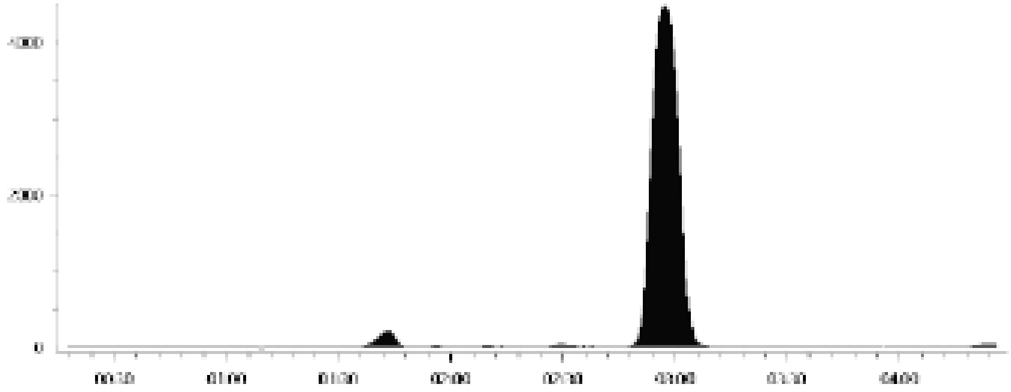

#	Compound	Retention Time	Area	% Area	Height
1		01:43.0	1083.7	2.9	200.37
2		02:57.5	36273.6	97.1	4460.43

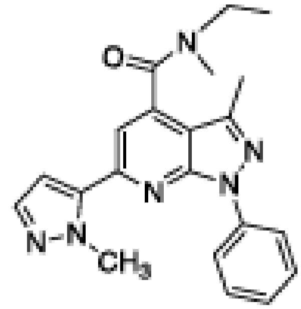

14k

miniLC

Method: 6 MINUTE ISOCRATIC 80% acn
Batch: LK
Sample: MSU-MLG-1-199
User: Guest
S/N: 0118000000
Date: 11/30/2022 4:54 AM

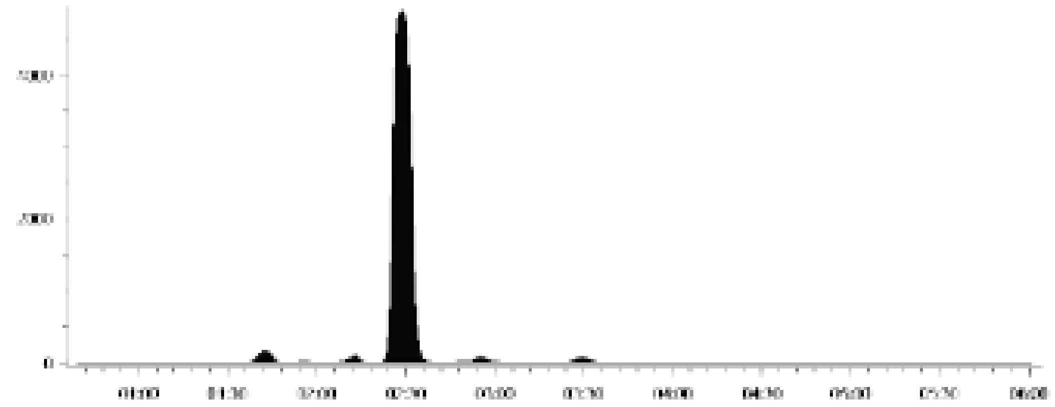

#	Compound	Retention Time	Area	% Area	Height
1		02:12.5	439.1	1.5	103.29
2		02:28.5	31401.4	95.9	4878.04
3		02:55.0	449.7	1.4	87.15
4		03:29.5	166.5	1.2	77.55

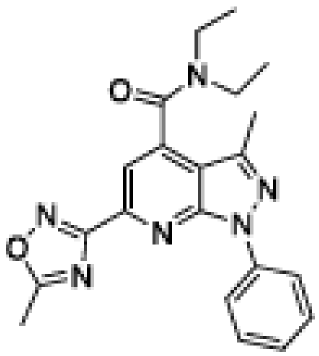

15

miniLC

Method: 6 MINUTE ISOCRATIC 80% acn
Batch: A
Sample: MSU-SMO-1-067AA
User: Administrator
S/N: 0118000000
Date: 09/16/2022 1:41 AM

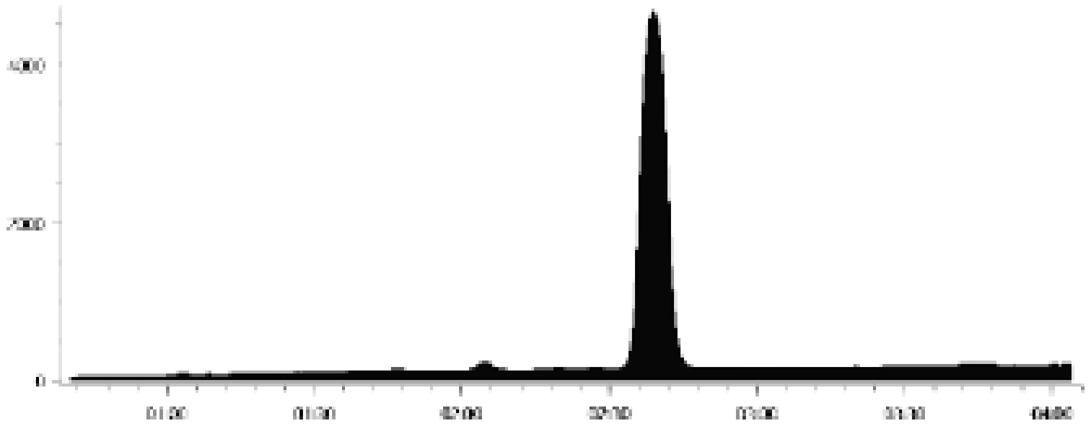

#	Compound	Retention Time	Area	% Area	Height
1		02:04.5	365.1	1.4	92.28
2		02:39.0	26196.2	98.6	4498.05

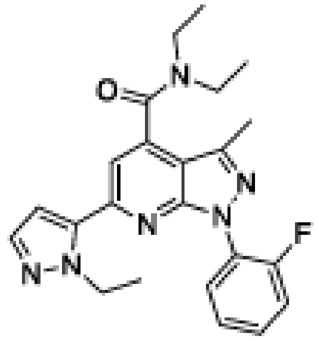

18a

miniLC

Method: 6 MINUTE ISOCRATIC 80% acn
Batch: ISOCRATIC ANALYSES
Sample: MSU-SMO-1-145AAA
User: Administrator
S/N: 0118000000
Date: 09/16/2022 1:23 AM

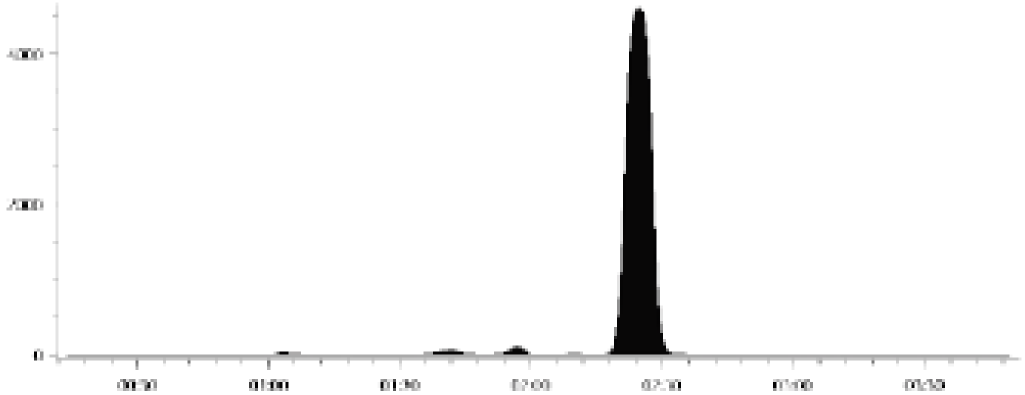

#	Compound	Retention Time	Area	% Area	Height
1		01:41.5	488.6	1.6	68.57
2		01:56.5	516.9	1.7	110.25
3		02:24.5	29706.3	96.7	4603.42

miniLC

Method: 6 MINUTE ISOCRATIC 80% acn
Batch: UY
Sample: MSU-FSH-1-49
User: Guest
S/N: 0118000000
Date: 11/30/2022 12:23 AM

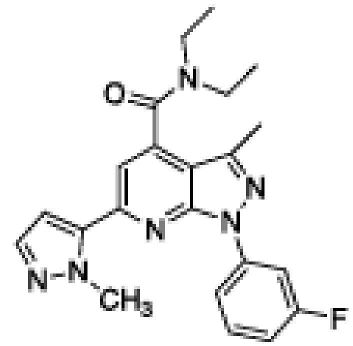

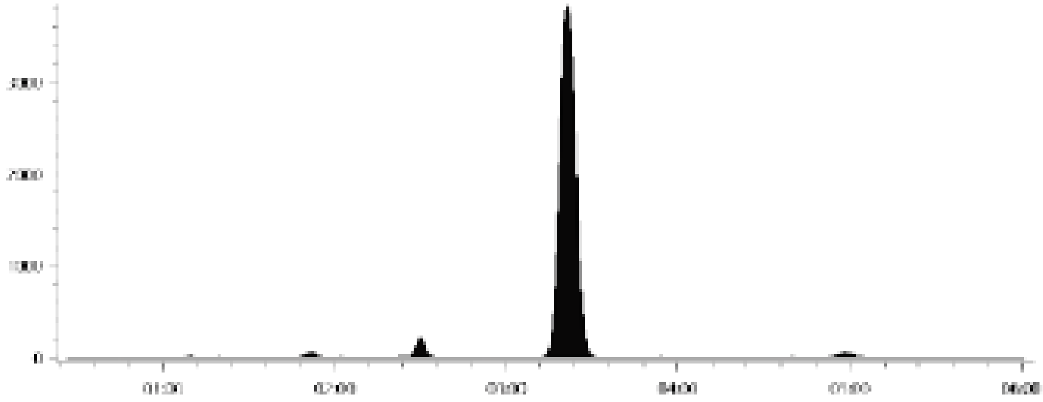

18b

#	Compound	Retention Time	Area	% Area	Height
1		01:52.0	239.3	0.9	58.27
2		02:30.0	634.7	3.3	159.78
3		03:21.5	24328.1	94.3	3822.70
4		04:59.0	407.5	1.6	56.85

miniLC

Method: 6 MINUTE ISOCRATIC 80% acn
Batch: NB
Sample: MSU-FSH-1-43
User: Administrator
S/N: 0118000000
Date: 11/29/2022 4:16 AM

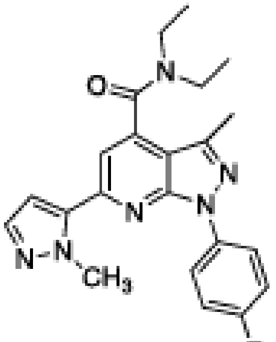

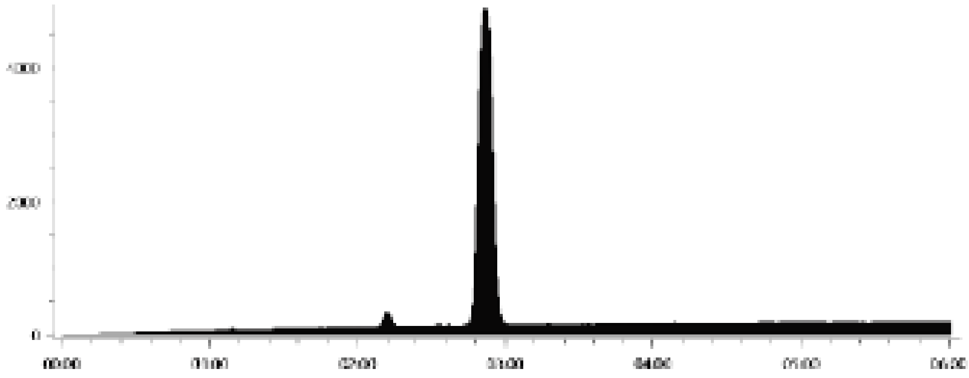

18c

#	Compound	Retention Time	Area	% Area	Height
1		02:12.0	1642.4	2.3	274.18
2		02:52.0	33036.2	97.7	4736.27

miniLC

Method: 6 MINUTE ISOCRATIC 80% acn
Batch: J
Sample: MSU-SMG-1-180
User: Guest
S/N: 0118000000
Date: 11/11/2022 11:36 PM

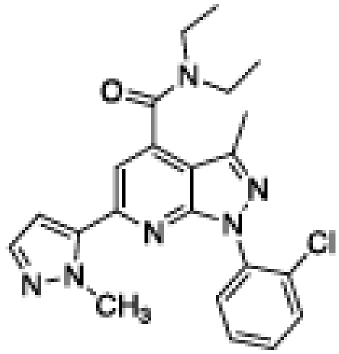

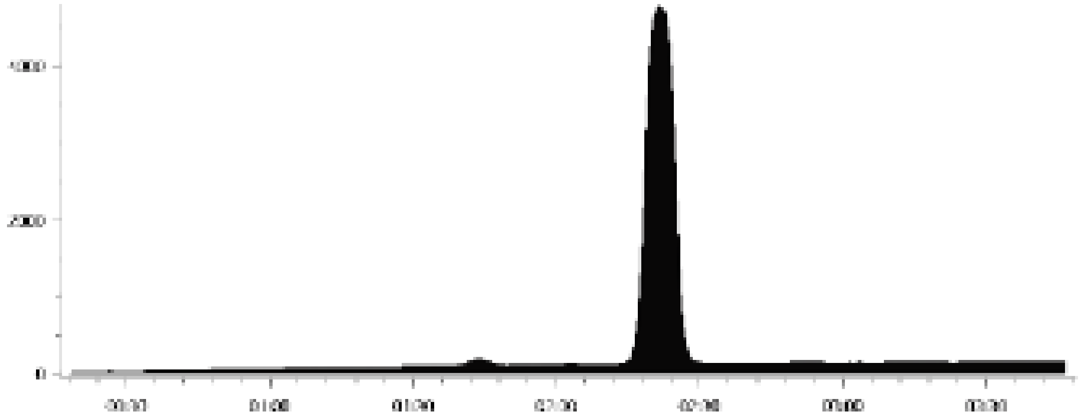

18d

#	Compound	Retention Time	Area	% Area	Height
1		01:44.0	436.7	1.4	90.47
2		02:21.5	30471.8	98.6	4627.16

miniLC

Method: 6 MINUTE ISOCRATIC 80% acn
Batch: kj
Sample: MSU-TSR-1-56
User: Guest
S/N: 0118000000
Date: 11/29/2022 11:36 PM

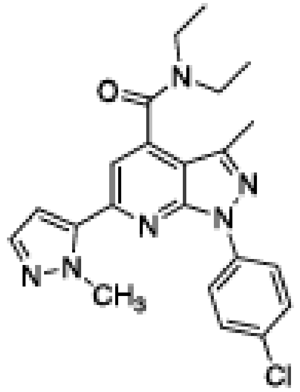

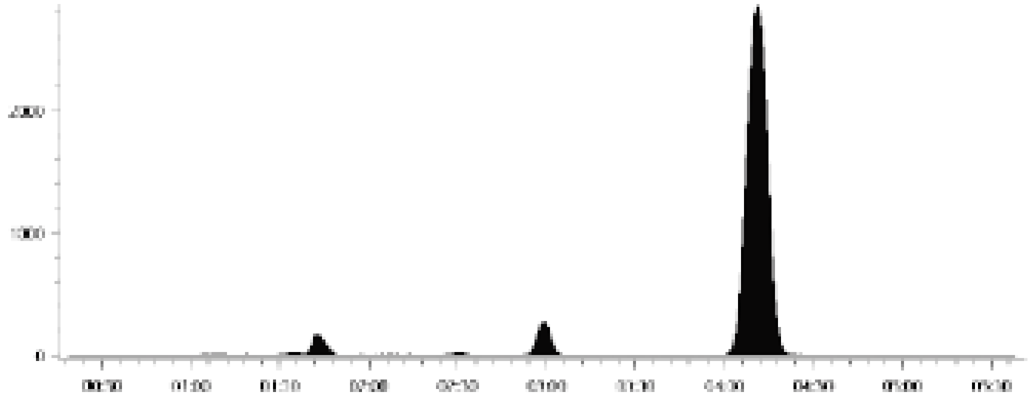

18e

#	Compound	Retention Time	Area	% Area	Height
1		01:43.0	750.4	3.0	147.89
2		02:59.0	1385.0	5.6	265.61
3		04:11.0	22693.3	91.4	2844.40

miniLC

Method: 6 MINUTE ISOCRATIC 80% acn
Batch: M
Sample: MSU-SMQ-1-80
User: Guest
S/N: 0118000000
Date: 11/09/2022 1:07 AM

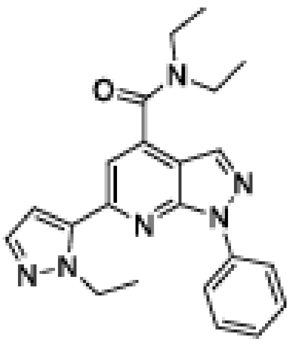

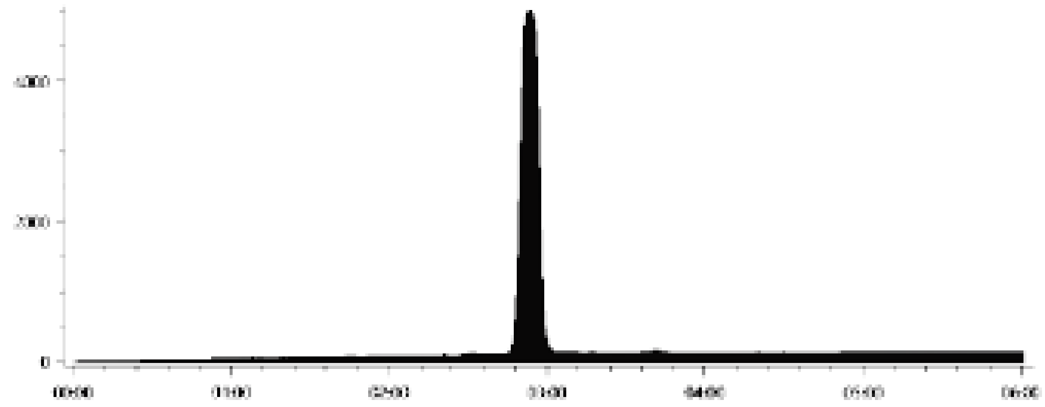

20a

#	Compound	Retention Time	Area	% Area	Height
1		02:53.5	47378.6	100.0	4940.43

miniLC

Method: 6 MINUTE ISOCRATIC 80% acn
Batch: U
Sample: MSU-SMQ-1-100
User: Guest
S/N: 0118000000
Date: 11/11/2022 11:06 PM

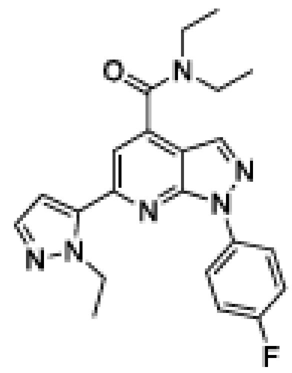

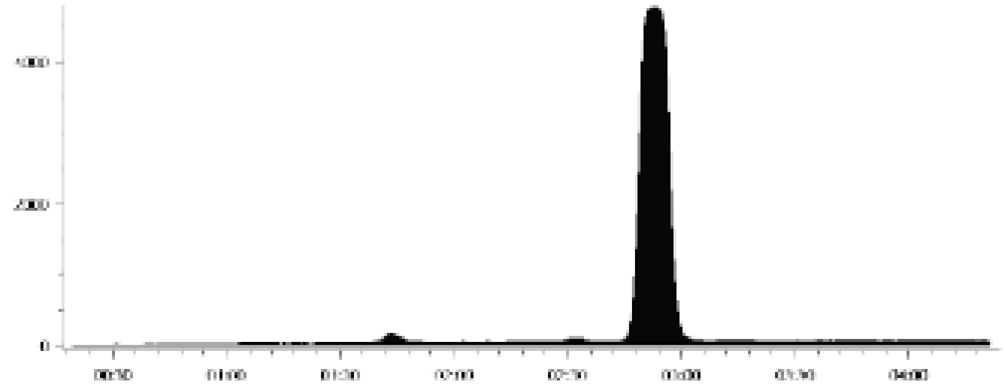

20b

#	Compound	Retention Time	Area	% Area	Height
1		01:43.5	690.9	1.7	126.81
2		02:32.0	221.5	0.5	50.06
3		02:53.0	40318.1	97.8	4694.40

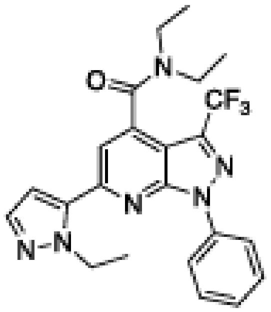

23a

miniLC

Method: 6 MINUTE ISOCRATIC 80% acn
Batch: J
Sample: MSU-SMQ-1-155
User: Guest
S/N: 0118000000
Date: 02/03/2023 10:15 AM

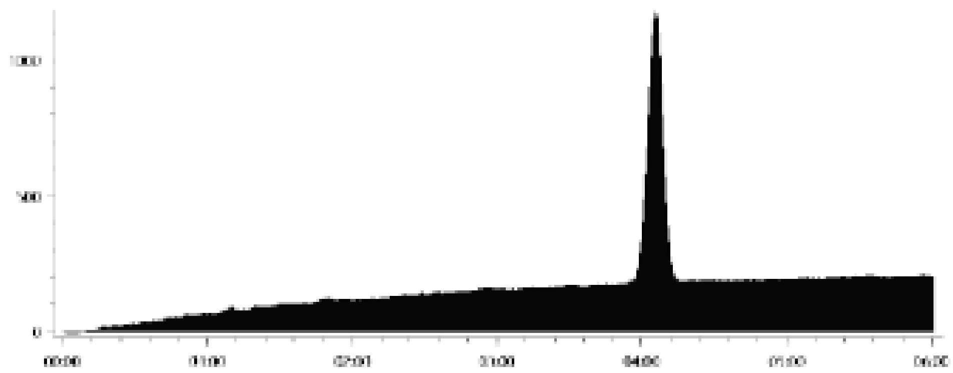

#	Compound	Retention Time	Area	% Area	Height
1		04:05.5	17695.4	100.0	1075.32

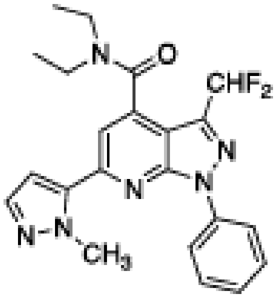

23b

miniLC

Method: 6 MINUTE ISOCRATIC 80% acn
Batch: M
Sample: MSU-SMQ-2-57
User: Guest
S/N: 0118000000
Date: 01/27/2023 10:49 PM

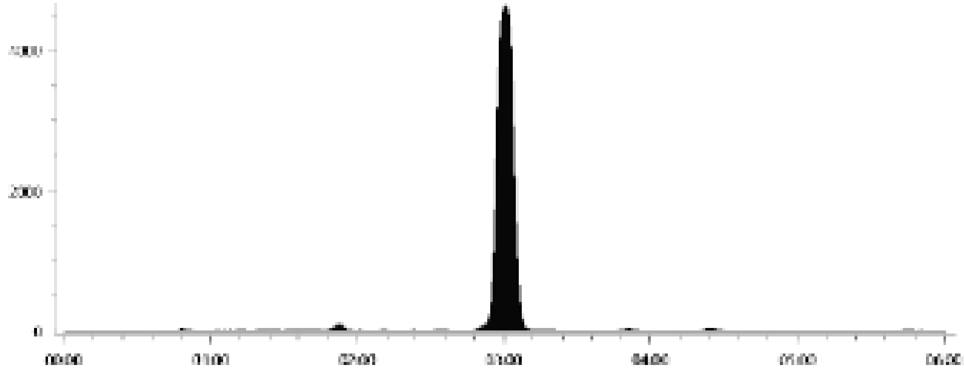

#	Compound	Retention Time	Area	% Area	Height
1		01:52.5	1121.2	1.0	89.76
2		03:00.5	36279.4	99.0	4638.15

miniLC

Method: 6 MINUTE ISOCRATIC 80% acn
Batch: NB
Sample: MSU-SMQ-2-154
User: Admin
S/N: 0118000000
Date: 05/02/2023 4:45 PM

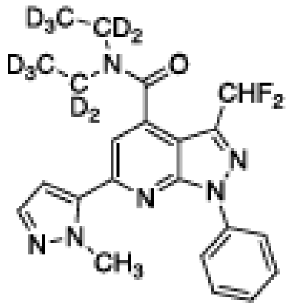

25

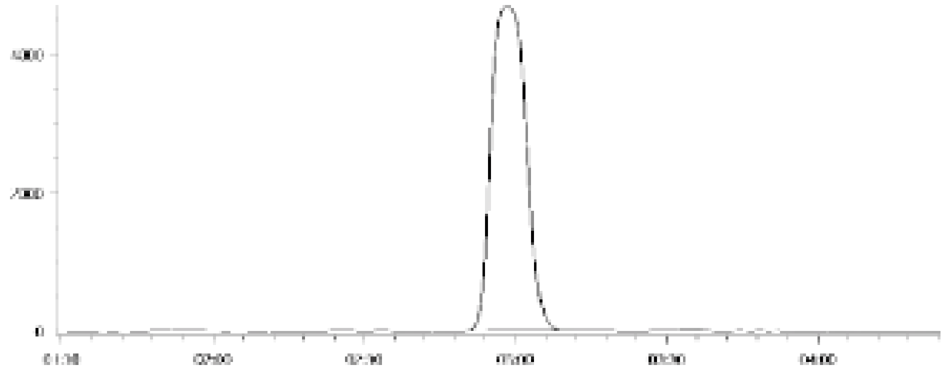

#	Compound	Retention Time	Area	% Area	Height
1		02:58.5	37354.3	100.0	4675.43

Mass spectrum of 25

==== Shimadzu LabSolutions Data Report ====

<Spectrum>

Line#:1 R.Time:11.817(Scan#:710)
MassPeaks:500
RawMode:Single 11.817(710) BasePeak:435(5905113)
BG Mode:None Segment 1 - Event 1

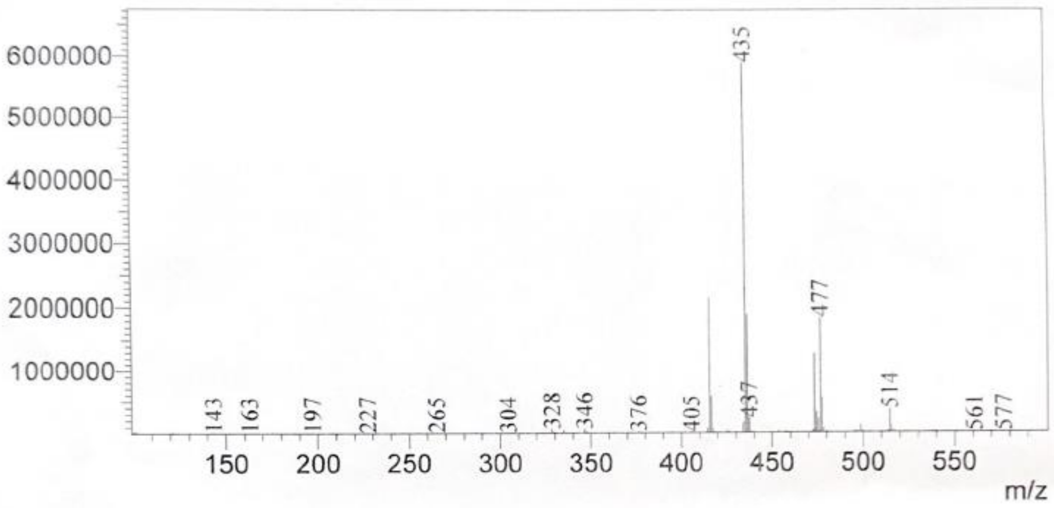

Expanded PDE Selectivity Screening 14b & 23b

Table S-1 Inhibitory Effects of the Compounds on PDE Activities

Inhibitors	% Inhibition									
	PDE4A1A	PDE4B1	PDE4C1	PDE4D7	PDE7A1	PDE7B	PDE8A1	PDE9A2	PDE10A1	PDE10A2
SMQ-2-057 (**23b**), 1 µM	1	5	15	16	2	1	1	1	4	2
SMQ-2-057 (**23b**), 10 µM	24	14	34	33	2	51	11	2	10	3
SMQ-1-148 (**14b**), 1 µM	5	2	14	1	0	0	1	2	3	2
SMQ-1-148 (**14b**), 10 µM	8	4	36	5	8	3	7	1	5	6
Apremilast ~ 0.1 x IC_{50}	9	10	8	8	-	-	-	-	-	-
Apremilast ~ 1 x IC_{50}	50	54	41	53	-	-	-	-	-	-
Apremilast ~ 10 x IC_{50}	93	94	87	93	-	-	-	-	-	-
BRL-50481 ~ 0.1 x IC_{50}	-	-	-	-	9	-	-	-	-	-
BRL-50481 ~ 1 x IC_{50}	-	-	-	-	51	-	-	-	-	-
BRL-50481 ~ 10 x IC_{50}	-	-	-	-	93	-	-	-	-	-
Dipyridamole ~ 0.1 x IC_{50}	-	-	-	-	-	10	10	-	-	-
Dipyridamole ~ 1 x IC_{50}	-	-	-	-	-	48	50	-	-	-
Dipyridamole ~ 10 x IC_{50}	-	-	-	-	-	86	89	-	-	-
Bay 73-6691 ~ 0.1 x IC_{50}	-	-	-	-	-	-	-	8	-	-
Bay 73-6691 ~ 1 x IC_{50}	-	-	-	-	-	-	-	52	-	-
Bay 73-6691 ~ 10 x IC_{50}	-	-	-	-	-	-	-	90	-	-
Papaverine ~ 0.1 x IC_{50}	-	-	-	-	-	-	-	-	11	9
Papaverine ~ 1 x IC_{50}	-	-	-	-	-	-	-	-	54	51
Papaverine ~ 10 x IC_{50}	-	-	-	-	-	-	-	-	91	95

Cell Viability and Protein Expression in Cell-Based Assay

Table S-2. PDE11A4 protein expression (normalized by Ponceau staining and expressed as a fold-change of DMSO) measured in drug-treated transiently-transfected HT-22 cell samples used in the PDE assay confirms that the decrease in PDE11A4 catalytic activity caused by these compounds is not explained by a reduction in expression of the protein itself.

	1	14b	25
**DMSO**	1.0±0	1.0±0.14	1±0
**0.1 μM**	1.32±0.27	1.02±0.10	1.16±0.12
**1 μM**	1.21±0.29	1.10±0.07	1.04±0.17
**10 μM**	1.12±0.17	0.819±0.14	1.25±0.19
**100 μM**	0.94±0.03	0.78±0.10	1.19±0.12

Neither 1 (failed normality; ANOVA on Ranks: H(4)=1.04, P=0.4), 14b (F(4,15)=1.46, P=0.263) nor 25b (F(4,15)=0.56, P=0.697) significantly changed PDE11A4 protein expression. Data expressed as mean ±SEM.

Table S-3. The number of intact transiently transfected cells counted in a separate study of drug-treated HT-22 cells confirms that the decrease in PDE11A4 catalytic activity caused by these compounds is not explained by a loss of PDE11A4-expressing cells.

	1	14b	25
**DMSO**	100±21.9	63.8±4.6	73±5
**0.1 μM**	92±6.2	51.2±4.3	77.5±7.2
**1 μM**	86.5±12.7	53±3.7	92±7.3
**10 μM**	88.3±12.3	61.8±4.0	78.5±9.1
**100 μM**	76.84±10.1	61.5±2.7	71.5±2.4

Sample images for quanitification were taken using an inverted Leica microscope with a 10x objective. All cells within the field of view showing expression of the GFP-PDE11A4 recombinant protein were counted by an experimenter blind to treatment. Neither 1 ($F(4,15)=0.38$, $P=0.817$), 14b ($F(5,18)=2.09$, $P=0.132$) nor 25b ($F(4,15)=1.50$, $P=0.251$) significantly changed the total number of cells expressing PDE11A4 protein. Data expressed as mean ±SEM.
